# Effects of rehabilitation and behavior change interventions on physical capacity and physical activity behavior following lumbar surgery for degenerative disease: A systematic review and meta-analysis

**DOI:** 10.1371/journal.pone.0347420

**Published:** 2026-04-20

**Authors:** José Manuel García-Moreno, Tyler Adams, Amber Beynon, Janine Vlaar Olthuis, Stephan U. Dombrowski, Richelle Witherspoon, Niels Wedderkopp, Jeffrey J. Hébert

**Affiliations:** 1 Faculty of Kinesiology, University of New Brunswick, Fredericton, New Brunswick, Canada; 2 School of Allied Health, Curtin University, Perth, Bentley WA, Australia; 3 Department of Psychology, University of New Brunswick, Fredericton, New Brunswick, Canada; 4 University of New Brunswick Libraries, Fredericton, New Brunswick, Canada; 5 Center for Research in Childhood Health, University of Southern Denmark, Odense, Denmark; 6 School of Allied Health, Murdoch University, Perth, Murdoch WA, Australia; Aichi Prefectural Mikawa Aoitori Medical and Rehabilitation Center for Developmental Disabilities, JAPAN

## Abstract

**Background:**

Rehabilitation and behavior change interventions are commonly used after lumbar surgery to improve recovery, but their effects on physical capacity and physical activity remain unclear. This study aimed to investigate the effectiveness of rehabilitation and behavior change interventions on physical capacity and physical activity behavior in patients following lumbar surgery for degenerative disease.

**Methods:**

EMBASE, MEDLINE, PsycINFO, and CENTRAL were searched from inception to September 2025 and reference lists were hand-searched. Randomized controlled trials assessing rehabilitation or behavior change interventions on physical capacity or physical activity behavior in adults with lumbar degenerative disc disease who underwent lumbar surgery were included. Review author pairs independently extracted data and assessed included studies. Risk of bias was assessed with the Cochrane tool, and study quality with the Grading of Recommendations Assessment, Development and Evaluation classification. Results were pooled using random-effects models and reported as standardized mean differences (SMD) with 95% confidence intervals (CI).

**Results:**

Exercise was more effective than minimal or usual care in improving trunk extension endurance in the immediate term (SMD, 1.54; 95% CI, 0.93–2.16). Supervised exercise outperformed self-directed exercise in improving trunk extension endurance in the immediate term (SMD, 1.28; 95% CI, 0.75–1.81). Psychologically informed rehabilitation was more effective than minimal or usual care in increasing physical activity levels in the intermediate term (SMD, 0.26; 95% CI, 0.02–0.49), but not in the immediate term (SMD, 0.17; 95% CI, −0.14 to 0.49). Physical activity advice did not increase physical activity levels compared to minimal or usual care in the immediate term (SMD, 0.21; 95% CI, −0.13 to 0.55). Prehabilitation was more effective than minimal or usual care in increasing physical activity levels in the intermediate term (SMD, 0.28; 95% CI, 0.03–0.53). Certainty of evidence ranged from low to moderate.

**Conclusions:**

For adults with lumbar degenerative disease who underwent lumbar surgery, exercise, especially supervised programs, improved trunk extension endurance in the immediate term. Psychologically informed rehabilitation and prehabilitation increased physical activity levels in the intermediate term, while physical activity advice showed no benefit. Findings are limited by low certainty of evidence and high risk of bias.

## Introduction

Lumbar degenerative diseases, such as disc herniation, and lumbar spinal stenosis, are widespread conditions with significant global impact. The prevalence of these conditions ranges from 55.2% to 84.5%, increasing with age [1]. Globally, the annual degenerative lumbar spine disease incidence is 3.6% [2]. These conditions often lead to pain, reduced mobility, and neurological deficits [3], and low back pain remains the leading cause of disability and work absenteeism worldwide [4].

The first treatment option for lumbar degenerative diseases is usually conservative management, which mainly includes rehabilitation and pain management [5]. When conservative treatment fails, surgery becomes the standard treatment [6]. There are different surgical techniques, such as decompression alone or decompression with fusion. While spinal surgery remains an effective option in select patients, it has some drawbacks, including muscle damage, an increased risk of infection, and potential mechanical instability [7].

To improve outcomes and reduce the negative consequences of lumbar surgery, rehabilitation is typically provided before or after the procedure [8]. Traditionally, rehabilitation has focused on recovering physical function and managing pain through specific exercises and education [8]. However, in recent years, behavior change interventions have also been increasingly integrated into rehabilitation programs to address psychological barriers such as kinesiophobia, low self-efficacy, and pain catastrophizing [9]. Despite these developments, rehabilitation practices still vary widely across centers and countries, with notable inconsistencies in patient restrictions, education, and the types of exercises prescribed [8]. This variability complicates the evaluation of rehabilitation outcomes. Although general recommendations for rehabilitation and behavior change interventions can be made based on the type of surgery [8], their effects on aspects such as physical capacity and physical activity behavior remain unclear.

Therefore, this systematic review aimed to investigate the effectiveness of rehabilitation and behavior change interventions on physical capacity and physical activity behavior in adult patients following lumbar surgery for lumbar degenerative disease.

## Methods

This systematic review was conducted in accordance with the Preferred Reporting Items for Systematic Reviews and Meta-Analyses (PRISMA) statement guidelines [10] and was registered with PROSPERO (CRD42020155495). The completed PRISMA checklist is available in S1 Table.

### Eligibility criteria

#### Type of patients.

Studies of adults (≥ 18 years) with lumbar degenerative conditions, such as lumbar spinal stenosis, lumbar foraminal stenosis, lumbar disc protrusion, prolapse or herniation, lumbar spondylosis, lumbar spondylolisthesis, and degenerative disc disease, who underwent lumbar surgery.

#### Type of interventions.

Studies had to focus on rehabilitation or behavior change interventions provided preoperatively to patients scheduled for lumbar spinal surgery, or within 12 months postoperative. Relevant rehabilitation interventions included physical treatments such as strengthening, stretching, and mobilization exercises, either self-directed or supervised. Behavior change interventions were defined as those using specific techniques (e.g., goal setting, exposure, feedback) to improve physical activity behavior by increasing physical activity levels, including, among others, time spent in moderate-to-vigorous physical activity (MVPA), daily step count, and reducing sedentary time. Since many interventions included multiple components (e.g., supervised exercise that also incorporated a cognitive behavioral intervention), a single trial could contribute to more than one comparison. Comparators included other rehabilitation or behavior change interventions, sham, placebo, or no treatment groups, which could be administered alone or in combination. Any eligible intervention could serve as either the experimental or comparator condition depending on the study design; however, only contrasts in which the intervention and comparator differed in intervention type were included in the systematic review comparisons and the meta-analysis. Detailed definitions of each intervention type are provided in S1 File.

#### Type of outcome measures.

The included studies had to report data on at least one of the following outcome measures for physical capacity: trunk flexion strength, trunk extension strength, trunk flexion endurance, trunk extension endurance, lower extremity strength, lower extremity endurance, walking capacity, walking speed, balance, or lumbar muscle function, or at least one of the following measures for physical activity behavior: self-reported or objectively measured time spent in MVPA, light physical activity, or sedentary behavior, or the number of daily steps. Reporting of adverse events or other harms was not required for inclusion, but such events were extracted when reported.

#### Type of study design.

We included peer-reviewed randomized controlled trials (RCTs) reported in English. Studies published in other languages would have required translation, which could lead to loss of meaning or contextual nuances and compromise the accuracy of data extraction.

### Search strategy

A three-step search strategy was implemented. Firstly, an initial exploratory search was performed in EMBASE, and an analysis of the text words contained in the titles, abstracts, and subject descriptors was performed. Second, a search using the keywords and subject terms obtained in step 1 was performed in EMBASE (Elsevier), MEDLINE (Ovid), PsycINFO (EBSCO), and Cochrane CENTRAL; RCTs were isolated using Cochrane’s *Highly Sensitive Filter for RCTs* and translations thereof [11]. Thirdly, the reference lists in all the selected articles were hand-searched to locate any additional research on this topic. The original search was conducted in November 2019 and subsequently updated in July 2024 and September 2025. A full search strategy for all databases is included in S2 File.

### Screening

A two-stage screening process was conducted independently by two review authors. In the first search, pairs of review authors from a panel of four (T.A., N.W., J.H., J.O.) independently performed the screening. For the second and third search, screening was conducted by two review authors (J.H., J.G.). Titles and abstracts were screened to identify studies potentially meeting eligibility criteria. Then, full-text articles were independently assessed for eligibility. Disagreements were resolved through discussion, and when necessary, a third review author (N.W.) was consulted for arbitration.

### Data extraction

In all three searches, data extraction was performed independently by two review authors using the same customized form. In the first search, data were extracted by one pair of review authors (T.A., A.B.), while the second and third data extraction was performed by another pair of review authors (J.G. J.O.). Disagreements were resolved through discussion, and a third review author (J.H.) was consulted for resolution. The review authors extracted information on the study population (e.g., age, sex, diagnosis), descriptions of the intervention and comparator, outcome measures, and the main findings of the included trials.

### Risk of bias

Risk of bias was assessed independently by two review authors. For the initial search, assessments were conducted by T.A. and A.B., and for the updated search by J.G. and A.B., using the Cochrane Risk of Bias tool [12]. Disagreements were resolved via discussion and arbitration with a third review author (J.H.) when necessary. A study was classified as high risk of bias if it was rated as unclear or high risk in any of the following domains: selection bias, attrition bias, reporting bias, or other bias. The remaining studies were classified as low risk of bias.

### Data synthesis and statistical analysis

Outcomes were categorized into four follow-up time points based on the end of the rehabilitation intervention. When this information was not reported, we used the time elapsed since surgery instead. The four categories included immediate (≤2 weeks), short-term (>2 weeks to ≤3 months), intermediate (>3 months to <12 months), and long-term (≥12 months) follow-up. When multiple time points fell within the same category, the time point closest to 1 week for immediate, 8 weeks for short-term, 6 months for intermediate, and 12 months for long-term was selected. Three independent review authors (J.G., J.H., N.W.), two physical therapists and one orthopaedic surgeon, all with expertise in clinical research, assessed the clinical diversity of the included trials, grouping studies only when they were similar across all major aspects, including participant characteristics, interventions, comparators, and outcomes, using clinical judgment. Disagreements were resolved through discussion, and if necessary, a third review author (A.B.), with a background in chiropractic and clinical research, acted as arbitrator.

Two review authors (J.G., J.H.) independently calculated the effect size of each trial using the standardized mean difference (SMD) with Hedges’ *g* [13], and resolved any discrepancies through discussion. Calculations were performed with Review Manager version 8.14.0 [14] with random-effects models. Statistical significance was defined as *p* < .05. The baseline-to-posttest differences were estimated for each study, where “posttest” refers to any follow-up time point after the intervention. A meta-analysis was performed when two or more studies shared the same intervention, comparator, outcome, and time frame, and a comparable sample population (e.g., diagnosis, surgical procedure). In trials where both objective and subjective measures were reported for the same outcome, the meta-analysis prioritized the objective data. For articles that did not provide sufficient data (e.g., mean or standard deviation), conversions were made to obtain the necessary information for the analysis, following the guidelines provided by Cochrane [15]. In studies with two intervention groups and one comparator, we applied Cochrane-recommended formulas to combine the intervention groups into a single group when they were sufficiently similar, allowing for appropriate pairwise comparison with the comparator [15]. When necessary, mean values were multiplied by −1 to ensure consistency in the direction of scales before standardization, as recommended by Cochrane [15]. A forest plot was created to visually and numerically represent the individual effects of each study and the average effect size. The magnitude of Hedges’ *g* was interpreted according to Hedges’ guidelines: a value of 0.2 indicating a small effect, 0.5 a medium effect, and 0.8 a large effect. Additionally, the 95% confidence intervals (CI) for the change scores were calculated, and the prediction intervals were also estimated. The CI was obtained using the Hartung and Knapp method [16] when (a) the between-study variance estimate was greater than 0 and (b) there were more than two study results. In all other cases, we used the Wald-type method, following the recommendations of Cochrane [17]. The tau^2^ statistic [18], obtained using the Restricted Maximum-Likelihood method [19], and the I^2^ statistic [20] were used to assess heterogeneity. Analyses of potential publication bias using Egger’s test and funnel plots [21] were planned but not performed due to an insufficient number of studies. Moderator analyses using analysis of variance for categorical variables and meta-regression for continuous variables were also not conducted for the same reason.

### Assessment of the certainty of the evidence

We assessed the certainty of the evidence using the Grading of Recommendations, Assessment, Development and Evaluation (GRADE) tool [22] and GRADEpro software [23]. Two review authors independently applied the tool and resolved any disagreements through discussion. The certainty of evidence was downgraded from “high” by one level for each of the following limitations: study design or execution (i.e., risk of bias), inconsistency, or imprecision. Specifically, downgrading occurred when (1) more than 50% of patients were from studies not assessed as low risk of bias; (2) I^2^ values exceeded 50%, indicating inconsistency; or (3) the total sample size was below 400, or when the 95% CI was wide enough that a clinical recommendation would differ depending on whether the upper or lower boundary represented the true effect, indicating imprecision [24]. We did not assess indirectness, as this review focused on a specific population, comparison, and outcome. Additionally, we did not consider publication bias due to the small number of trials included in each analysis [12].

## Results

### Search results

**Fig 1:**
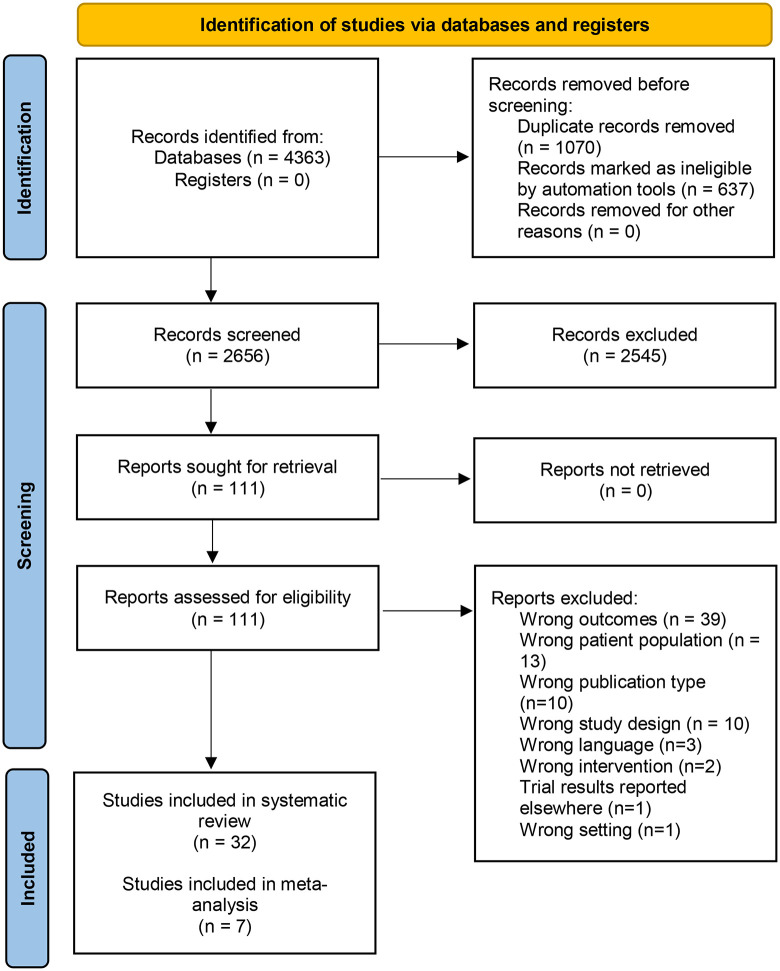
Flow diagram of study selection.

The search identified 4,363 records, of which 111 were retained after title and abstract screening. After full-text assessment, 32 reports from 30 unique trials involving 2,527 patients were included, with 1,307 patients in the intervention groups and 1,220 patients in the comparator groups. The meta-analysis incorporated 7 trials with a total of 510 patients, including 284 in the intervention groups and 226 in the comparator groups (Fig 1). Reasons for excluding studies at the full-text assessment stage are provided in S2 Table.

### Included studies

Among the 32 reports [25–56], two were follow-up reports derived from two original trials [36,40]. The reports were published between 1994 [38] and 2025 [46,49,52–54]. The mean age of the participants ranged from 36.0 [38] to 71.6 years [47]. All reports included both men and women. A total of 27 trials included one intervention group and one comparator group [26–29,31–55], while three trials included two intervention groups and one comparator group [25,30,56]. The diagnoses of the patients were lumbar disc protrusion/prolapse/herniation in 12 trials [25,28–30,32,33,37–39,42,43,56], lumbar degenerative disc disease in seven trials [26,31,34,48,50–52], a combination of degenerative diseases in eight trials [40,41,44–46,49,53–55], lumbar stenosis in two trials [27,47], and lumbar spondylolisthesis in one trial [35,36].

There were diverse rehabilitative interventions and comparators. Exercise, either supervised or self-directed, was the most frequently used intervention and was included in 22 trials [25,27–31,34–39,41,43,44,46,47,49,50,52–54,56]. Other interventions included psychologically-informed rehabilitation in five trials [26,40,44,45,48,55], physical activity advice in five trials [35,36,40,42,44,45,48], and prehabilitation in eight trials [27,40,44,45,47,49,50,54,55]. The most common comparators were minimal or usual care, reported in 18 trials [25–27,29,30,35–37,39–45,47,48,50,55,56], and exercise, either supervised or self-directed, in 16 trials [28,30–34,38,44,46,49,51–54,56].

We categorized intervention vs. comparator contrasts as: exercise versus minimal or usual care in 13 trials across 14 reports [25,27,29,30,35–37,39,41,43,44,47,50,56], supervised exercise versus self-directed exercise in eight trials across eight reports [28,30,31,34,38,44,53,56]; psychologically informed rehabilitation versus minimal or usual care in five trials across six reports [26,40,44,45,48,55]; physical activity advice versus minimal or usual care in five trials across seven reports [35,36,40,42,44,45,48]; and prehabilitation versus minimal or usual care in six trials across seven reports [27,40,44,45,47,50,55]. Seven trials were excluded from the contrasts because both the intervention and comparator groups received the same type of intervention, differing only in intensity or delivery parameters, and therefore did not constitute a conceptually distinct comparison [32,33,46,49,51,52,54]. Details of the contrast tables are included in S3 File.

Trials reported 14 different outcomes. Trunk flexion endurance was included in five trials [25,30,32,53,56]; trunk extension endurance in nine trials [25,29,30,32,36,43,47,53,56]; trunk flexion strength in seven trials [32,34,36,38,41,47,53]; trunk extension strength in nine trials [28,32,34,36,38,39,41,47,53]; lateral trunk flexion strength (right and left) in one trial [41]; lumbar multifidus muscle function in one trial [33]; transversus abdominis activation capacity in one trial [49]; lower extremity endurance in nine trials [26,27,31,32,37,41,43,47,50]; lower extremity strength in one trial [47]; walking capacity in seven trials [40,41,43,45,46,51,53,54]; walking speed in five trials [26,27,31,43,45]; balance in two trials [52,53]; physical activity levels in seven trials [32,35,40,42,44,45,48,55]; and the presence or absence of adverse events in eight trials [33,35,36,43–48]. Detailed information on sample populations, interventions, comparisons, outcomes, and main findings is summarized in Table 1.

**Table 1:** Characteristics of included studies.

Study	Sample population	Intervention treatment	Comparison treatment	Outcomes	Main findings*
Abdi (2023)	90 patients (G1 = 30, G2 = 30, G3 = 30) with lumbar disc herniation who had undergone a single-level lumbar laminectomy and discectomy; mean age (G1 = 44.2, G2 = 44.9, G3 = 42.9); female (G1 = 46.4%, G2 = 51.7%, G3 = 50%)	G1: 24 sessions over 8 weeks (3 times a week, 45 min each) of unsupervised home-based exercise based on pelvic tilt, single knee to chest, double knee to chest, partial sit-up, hamstring stretch, hip flexor stretch, and squat, starting 6 weeks after surgery G2: 24 sessions over 8 weeks (3 times a week, 45 min each) of unsupervised home-based exercise based on lying prone, prone on elbows, standing extension, lying flexion, sitting flexion, and standing flexion, starting 6 weeks after surgery	G3: conventional postoperative rehabilitation including early walking and physiotherapy	Trunk flexion endurance (isometric test), and trunk extension endurance (modified Sorensen test), immediately after the end of the intervention (3.5 months after surgery)	The G1 obtained better results in the trunk flexion endurance than G2 and G3 The G2 obtained better results in the trunk extension endurance than G1 and G3
Archer (2016)	86 patients (G1 = 43, G2 = 43) with lumbar degenerative condition undergoing a laminectomy with or without arthrodesis; mean age (G1 = 56.9, G2 = 58.4); female (G1 = 58%, G2 = 53%)	G1: 6 sessions (1 in-person and 5 remote) over 6 weeks of cognitive-behavioral-based physical therapy, starting 6 weeks after surgery	G2: 6 sessions (1 in-person and 5 remote) over 6 weeks of usual postoperative education, starting 6 weeks after surgery	Lower extremity endurance (5-times sit-to-stand test), and walking speed (10-meter walk test), immediately and 3 months after the end of the intervention (3 and 6 months after surgery)	Improved the lower extremity endurance immediately favoring the G1
Chen (2015)	60 patients (G1 = 29, G2 = 31) with lumbar stenosis scheduled for decompression surgery; mean age (G1 = 51.8, G2 = 52.1); female (G1 = 45%, G2 = 55%)	G1: Preoperative education and postoperative exercises including mobilization strategies, core stability exercises, and muscle strengthening. Additionally, 30 minutes of daily supervised exercise during hospitalization	G2: Usual care based on instructions concerning post-operative care provided by the neurosurgical team	Walking speed (15-meter walk test), and lower extremity endurance (5-times sit-to-stand test), at discharge, 1, 3, and 6 months after discharge	No between-group differences in any outcome
Choi (2005)	75 patients (G1 = 35, G2 = 40) with single-level disc herniation who had undergone discectomy; mean age (G1 = 51.1, G2 = 42.0); female (G1 = 43%, G2 = 55%)	G1: 4 weeks of self-directed lumbar conditioning exercises, starting 2 weeks after surgery. Additionally, 12 weeks of supervised lumbar extensor strengthening, aerobic, and limb-strengthening exercises, starting 6 weeks after surgery	G2: 16 weeks of self-directed lumbar conditioning exercises, starting 2 weeks after surgery	Trunk extension strength (isometric dynamometer), immediately after the end of the intervention (4.5 months after surgery)	Improved lumbar extension strength favoring the G1
Dolan (2000)	20 patients (G1 = 9, G2 = 11) with prolapsed lumbar disc who had undergone microdiscectomy; mean age (G1 = 39.2, G2 = 42.7), female (G1 = 0%, G2 = 27%)	G1: Usual postoperative advice regarding exercise and return to activity was provided during the first 6 weeks after surgery. Additionally, 8 sessions over 4 weeks of supervised aerobic, stretching, and strength and endurance exercises were conducted, starting 6 weeks after surgery	G2: Usual postoperative advice regarding exercise and return to activity was provided during the first 6 weeks after surgery	Trunk extension endurance (Sorensen test), immediately, 4 and 10.5 months after the end of intervention (2.5, 6.5, and 13 months after surgery)	No between-group comparisons reported
Filiz (2005)	60 patients (G1 = 20, G2 = 20, G3 = 20) with single-level lumbar disc herniation who had undergone single-level lumbar discectomy; mean age (G1 = 38.2, G2 = 41.3, G3 = 40.2); female (G1 = 50%, G2 = 40%, G3 = 55%)	G1: 24 sessions over 8 weeks (3 times a week, 1.5 hours each) of supervised ‘intensive’ dynamic lumbar stabilization exercises. Additionally, 4 sessions of postoperative back education involving basic body mechanics, starting 1 month after surgery. G2: 1 session learning ‘classical’ McKenzie and Williams exercises. 24 sessions over 8 weeks (3 times a week) of self-directed ‘classical’ McKenzie and Williams exercises. Additionally, 4 sessions of postoperative back education involving basic body mechanics, starting 1 month after surgery	G3: Advice to be as active as possible	Trunk flexion and extension endurance (Ito lumbar trunk muscle endurance test), immediately after the end of the intervention (3 months after surgery)	Improved trunk flexion and extension endurance favoring the G1 and G2 over G3, and the G1 over G2
Greenwood (2019)	52 patients (G1 = 25, G2 = 27) with degenerative/congenital conditions undergoing lumbar fusion surgery; mean age (G1 = 55.9, G2 = 52.6); female (G1 = 54%, G2 = 59%)	G1: 10 weekly sessions of supervised group education (< 20 min per session) and exercise program (<60 min per session) that included a low-tech cardiovascular, range of movement, limb and spine strengthening exercises, and peer support. Additionally, usual hospital-based postoperative care and advice, starting 3 months after surgery	G2: referral to local physiotherapist. Commonly comprised of 6 sessions (30 min per session) of physiotherapy including individualized exercise-based self-management postoperatively. Additionally, usual hospital-based postoperative care and advice	Walking speed (50-yard walk test), and lower extremity endurance (sit-to-stand test), 2 weeks and 6.5 months after the end of the intervention (6 and 12 months after surgery)	No between-group difference in any outcome
Häkkinen (2005)	126 patients (G1 = 65, G2 = 61) with lumbar disc prolapse undergoing lumbar disc surgery; mean age (G1 = 39, G2 = 39); female (G1 = 45%, G2 = 43%)	G1: 12 months of self-directed strengthening, stretching, aerobic, and stabilization exercise program, starting 2 months after surgery	G2: 12 months of self-directed stretching, aerobic, and stabilization exercise program, starting 2 months after surgery	Physical activity (self-reported leisure time), trunk flexion and extension strength (isometric dynamometer), trunk flexion endurance (sit-up test), trunk extension endurance (arch-up test), lower extremity endurance (number of squats), immediately after the end of the intervention (14 months after surgery)	No between-group comparisons were reported in the physical activity outcomes No between-group difference in any other outcome
Hebert (2015)	61 patients (G1 = 29, G2 = 32) with lumbar disc herniation scheduled to undergo single-level lumbar discectomy; mean age (G1 = 40.6, G2 = 40.2); female (G1 = 55%, G2 = 47%)	G1: 8 sessions over 8 weeks of supervised exercise with daily self-directed exercises comprising general trunk strengthening, aerobic, range of motion, and isometric contractions of the lumbar multifidus and transverse abdominis, starting 2 weeks after surgery	G2: 8 sessions over 8 weeks of supervised exercise with daily self-directed exercises comprising general trunk strengthening, aerobic, and range of motion, starting 2 weeks after surgery	Lumbar multifidus muscle function (real-time ultrasound imaging), immediately after the end of the treatment (2.5 months after surgery)	No between-group differences No adverse events related to the exercise interventions
Huang (2023)	28 patients (G1 = 14, G2 = 14) with lumbar degenerative disease who had undergone lumbar fusion; mean age (G1 = 49.6, G2 = 54.3); female (G1 = 42.8%, G2 = 50%)	G1: 2 sessions (60 min each) of supervised aquatic training, and 18 sessions over 6 weeks (3 days a week, 60 min each) of unsupervised home exercise, starting 4 weeks after surgery. The aquatic exercise consisted of 5 min of warm-up with stretching and walking, 50 minutes of main exercises, and 5 min of cooldown stretching exercises. The home exercise was based on general lumbar stability exercises	G2: 30 sessions over 6 weeks (5 sessions per week, 60 min each) of unsupervised home exercise, starting 4 weeks after surgery. The home exercise was based on general lumbar stability exercises	Trunk flexion and extension strength (isometric dynamometer), immediately after the end of the intervention (2.5 months after surgery)	The G1 obtained significant differences from G2 in trunk extension strength
Ilves (2017)	98 patients (G1 = 48, G2 = 50) with spondylolisthesis who had undergone lumbar spine fusion; mean age (G1 = 59, G2 = 58); female (G1 = 71%, G2 = 76%)	G1: 12 months of progressive self-directed aerobic and back-specific exercises with fear-avoidance counseling. The physiotherapist gave each patient instructions on exercises with booster sessions every second month (6 sessions in total). Advice to increase daily steps via walking 3 or more times per week for 25–30 mins per session, starting 3 months after surgery	G2: Usual care comprising 1 information session 3 months after surgery with instructions for self-directed exercise: light abdominal, back, and hip muscle endurance exercises, stretching, and balance training (1-leg standing) 3 times per week	Physical activity (International Physical Activity Questionnaire), immediately after the end of the intervention (15 months after surgery)	No between-group differences No adverse events related to the intervention
Ilves (2022)	98 patients (G1 = 48, G2 = 50) with spondylolisthesis who had undergone lumbar spine fusion; mean age (G1 = 59, G2 = 58); female (G1 = 71%, G2 = 76%)	G1: 12 months of progressive self-directed aerobic and back-specific exercises with fear-avoidance counseling. The physiotherapist gave each patient instructions on exercises with booster sessions every second month (6 sessions in total). Advice to increase daily steps via walking 3 or more times per week for 25–30 mins per session, starting 3 months after surgery	G2: Usual care comprising 1 information session 3 months after surgery with instructions for self-directed exercise: light abdominal, back, and hip muscle endurance exercises, stretching, and balance training (1-leg standing) 3 times per week	Trunk flexion and extension strength (isometric dynamometer), trunk extension endurance (Sorensen test), immediately after the end of the intervention (15 months from surgery)	No between-group differences in any outcomes No adverse events related to the intervention
Janssens (2016)	25 patients (G1 = 12, G2 = 13) with single-level disc herniation who had undergone lumbar microdiscectomy; mean age (G1 = 46, G2 = 46); female (G1 = 58%, G2 = 54%)	G1: 8–15 sessions over 12 weeks of supervised, individualized education, ergonomic advice, mobilization, and motor control and neurodynamic exercises, starting 2 weeks after surgery	G2: 12 weeks of usual care including basic ergonomic instruction and advice to stay active, starting 2 weeks after surgery	Lower extremity endurance (5-times sit-to-stand test), 3.5 months after the end of the intervention (6 months after surgery)	No between-group differences
Johannsen (1994)	27 patients (G1 = 11, G2 = 16) with lumbar disc herniation at L4 or L5 who had undergone lumbar discectomy; median age (G1 = 39, G2 = 36); female (G1 = 36%, G2 = 25%)	G1: 24 sessions over 3 months (2 days a week, 1 hour each) of supervised exercise based on the lower back, higher back, buttock, and abdominal strengthening, endurance, and stretching exercises, starting 4–6 weeks after surgery	G2: 1 session of instruction to perform a self-directed 10 minutes of jogging, back, abdomen, hip abductors and adductors, and quadriceps strengthening exercises and stretching exercises program twice per week, starting 4–6 weeks after surgery	Trunk flexion and extension strength (isokinetic dynamometer), immediately and 3 months after the end of the intervention (4 and 7 months after surgery)	The G2 obtained significant differences from G1 in trunk extension strength immediately
Ju (2012)	14 patients (G1 = 7, G2 = 7) with lumbar disc herniation who had undergone lumbar disc herniation surgery; mean age (G1 = 45.2, G2 = 46.2); females (N/A)	G1: 36 sessions over 12 weeks (3 times per week, 70 minutes each) of supervised lumbar extension strengthening and progressive resistance exercises, starting approximately 15 days after surgery	G2: 12 weeks of rest	Trunk extension strength (isometric dynamometer), immediately after the end of the intervention (3.5 months after surgery)	No between-group comparisons reported
Kemani (2024)	118 patients (G1 = 59, G2 = 59) with disc herniation, foraminal stenosis, or isthmic spondylolisthesis scheduled for lumbar fusion surgery; mean age (G1 = 44.8, G2 = 46.7), female (G1 = 56%, G2 = 51%)	G1: 4 sessions (1 hour each) of prehabilitation, starting 8–12 weeks before surgery. Additionally, there was a half-hour follow-up session via phone two weeks after the surgery. These sessions, based on cognitive-behavioral techniques, focused on encouraging physical activity and addressing psychological risk factors before the surgery	G2: conventional care based on a single session of information about the postoperative mobilization routine, an introduction to a core exercise program to be initiated the day after surgery, and encouragement to stay active and start the recommended exercises before surgery	Physical activity (steps per day, time spent in sedentary, MVPA and light PA using an accelerometer), walking capacity (5-min walk test), walking speed (50-foot walk test), 1 week before surgery, 3 and 8 weeks after surgery, and 3, 6, 12, 24 months after surgery	No between-group differences in any outcome No adverse events were reported
Kernc (2018)	27 patients (G1 = 14, G2 = 13) with one-level degenerative isthmic spondylolisthesis or degenerative disc disease (with or without spinal stenosis) who had undergone lumbar spine fusion; mean age (G1 = 60.3, G2 = 61.1); females (G1 = 64%, G2 = 31%)	G1: 18 sessions over 9 weeks (2 sessions a week), of supervised trunk muscle strength training, starting 3 weeks after surgery. The first 5 weeks were composed of isometric strengthening exercises focused on the muscles of extension, flexion, and lateral flexion of the trunk and interferential electrical therapy. Weeks 6–9 were composed of the same exercises on strength machines with longer duration, leg adduction and hip extension exercises with abdominal activation, and static stretching exercises were added	G2: usual postoperative care not including exercise or physiotherapy for the first 3 months after surgery	Walking capacity (6-min walk test), lower extremity endurance (30-second chair stand test), and trunk flexion, extension, right and left flexion strength (isometric dynamometer), immediately and 15 months after the end of the intervention (3 and 18 months after surgery)	The G1 obtained statistically better results than G2 in the walking capacity and trunk strength immediately
Kjellby-Wendt (2002)	52 patients (G1 = 26, G2 = 26) with lumbar disc herniation who had undergone lumbar discectomy; mean age (G1 = 41, G2 = 39); female (G1 = 31%, G2 = 23%)	G1: 4 supervised sessions and daily self-directed exercises for 12 weeks, starting 1 day after surgery. The first 6 weeks of the program comprised advice about coping and remaining active, lumbar and leg range of motion exercises. The second 6 weeks consisted of the above and strengthening trunk extensor exercises, spinal stabilization exercises, and more intensive cardiovascular exercises	G2: 3 supervised sessions and daily self-directed exercises for 12 weeks of less active rehabilitation, starting 1 day after surgery. The first 6 weeks of the program comprised abdominal and leg strengthening exercises. The second 6 weeks included the above and spine ROM exercises (trunk flexion and lateral flexion)	Physical activity (self-reported questionnaire), 5–7 years after the end of the intervention and after surgery	No between-group comparisons reported
Kulig (2009)	98 patients (G1 = 51, G2 = 47) with disc protrusion who have undergone a single-level lumbar microdiscectomy; mean age (G1 = 39.2, G2 = 41.4); female (G1 = 43%, G2 = 49%)	G1: 36 sessions over 12 weeks (3 times per week) of supervised back extension strength, back extensor strength exercises, resistance training exercises, therapeutic mat and upright exercises with a progressive increase in load, and endurance training and 1 session (1 hour) of back care education, starting 4–6 weeks after surgery	G2: 1 session (1 hour) of back care education 4–6 weeks after surgery	Lower extremity endurance (5-times sit-to-stand test), walking speed (50-foot walk test), walking capacity (5-minute walk test), and trunk extension endurance (modified Sorensen test), immediately after the end of intervention (4–4.5 months after surgery)	Improved walking capacity and trunk extension endurance favoring the G1 compared to G2 No adverse events related to interventions
Lindbäck (2018)	197 patients (G1 = 99, G2 = 98) with disc herniation, spinal stenosis, spondylolisthesis (grades 1–2), or degenerative disc disease scheduled for surgery; mean age (G1 = 58, G2 = 61); female (G1 = 54%, G2 = 52%)	G1: 18 sessions over 9 weeks of pre-surgery physiotherapy including specific exercises and mobilization or motor control exercises or traction, supervised exercise program, behavior approach to increased physical activity level, and decreased fear-avoidance behavior. Additionally, postoperative rehabilitation based on a self-directed exercise program including feedback on posture and walking, a home exercise program, and daily walking	G2: usual care including information about surgery, postoperative rehabilitation, and advice to continue physical activity. Additionally, postoperative rehabilitation based on a self-directed exercise program including feedback on posture and walking, a home exercise program, and daily walking	Physical activity (self-reported questionnaire), 3 months and 1 year after surgery	Patients in the G1 were more physically active at 3 months and 1 year than patients in G2 No adverse events were reported
Lotzke (2019)	118 patients (G1 = 59, G2 = 59) with disc herniation, foraminal stenosis, or isthmic spondylolisthesis scheduled for lumbar fusion surgery; mean age (G1 = 44.8, G2 = 46.7), female (G1 = 56%, G2 = 51%)	G1: 4 sessions (1 hour each) of person-centered prehabilitation, starting 8–12 weeks before surgery and 1 telephone session 2 weeks after surgery. Sessions included spinal health education and cognitive-behavioral approaches to improve activity-related behaviors, physical activity despite pain, psychological risk factors, improve patient self-efficacy, and avoidance of beliefs and fear after surgery	G2: 1 preoperative session of usual care including information about post-operative mobilization, core exercises, and advice to stay active after surgery	Physical activity (MVPA, light PA, and sedentary time using an accelerometer), walking capacity (5-minute walk test), walking speed (50-foot walk test), 1 week before and 3 and 6 months after surgery	No between-group differences in any outcome No adverse events were reported
Lu (2025)	52 patients (G1 = 26, G2 = 26) with lumbar spinal stenosis, lumbar spondylolisthesis, or lumbar disc herniation who had undergone lumbar fusion surgery; mean age (G1 = 56.5, G2 = 55.5); female (G1 = 61.5%, G2 = 53.8%)	G1: 40 sessions over 10 weeks with progression in intensity. During the first 2 weeks after surgery patients performed lower extremity active mobilization and muscle strengthening. From weeks 2–4 they added hip muscle activation. From weeks 4–8 trunk and hip muscle activation. From weeks 8–12 hip abduction, extension and standing squat exercises with the supervision of family members and therapist-guided progression by phone	G2: Patient education and strengthening exercises in bed after surgery, and after discharge patients were advised to continue strengthening independently. In addition, they also received the same intervention as G1 from 12 to 24 weeks after surgery	Walking capacity (6-minute walk test) immediately and 3 months after the end of the intervention (3 and 6 months after surgery)	G1 had greater walking capacity compared to G2 immediately after the intervention, with no differences at 6 months. Adverse events were reported in 19.2% of patients in G1 and 15.4% in G2, including fixation loosening, hospital readmissions, fatigue, back pain, wound infections and palpitations
Marchand (2021)	68 patients (G1 = 35, G2 = 33) with lumbar spinal stenosis awaiting lumbar surgery; mean age (G1 = 66.2, G2 = 71.6); female (G1 = 40%, G2 = 42%)	G1: 18 sessions over 6 weeks, (3 times per week, 30 minutes each) of supervised warm-up (cycling or walking), trunk, hip, and lower extremity muscle strengthening exercises with progression in difficulty delivered before surgery. In addition, standardized written information on postural recommendations one day before surgery	G2: Usual preoperative management and advice. Standardized written information on postural recommendations one day before surgery	Trunk extension endurance (modified Sorensen test), trunk flexion and extension strength (isometric dynamometer), lower extremity endurance (30-seconds sit-to-stand test), lower extremity strength (isometric dynamometer), completed pre-surgery (after intervention), and 1.5 months after surgery	G1 had greater trunk flexion strength, trunk extension endurance, and lower extremity endurance after the intervention than G2 No adverse events were reported
Master (2024)	16 patients (G1 = 8, G2 = 8) with lumbar degenerative disease who had undergone lumbar laminectomy with or without fusion; mean age (G1 = 65.4, G2 = 63); female (G1 = 50%, G2 = 50%)	G1: 8 weekly sessions based on motivational interviewing and the use of a wearable watch with supervised daily step goal tracking, starting 2 weeks after surgery	G2: postoperative usual care based on lifting restrictions, advice to stay active, and oral analgesics as needed	Physical activity (steps per day, and MVPA using an accelerometer), 2 weeks and 3.5 months after the end of the intervention (3 and 6 months after surgery)	No between-group comparisons reported No adverse events were reported
Nie (2025)	395 patients (G1 = 214, G2 = 181) with lumbar spinal stenosis, disc herniation, spondylolisthesis, degenerative lumbar scoliosis, or adjacent segment disease who had undergone lumbar fusion; mean age (G1 = 60.9, G2 = 61.3), female (G1 = 44.9%, G2 = 49.7%)	G1: Before surgery patients received perioperative information and 9 sessions over 3 days (3 times per day, 20 minutes each) of supervised exercise including core stabilization and motor control training. From the first day after surgery they continued with supervised motor control training (3 times per day, 20 minutes each) for 4 weeks with progression in difficulty, and from week 5 they underwent an additional 8 weeks of core stabilization similar to G2 (3 times per day, 20 minutes each)	G2: Before surgery patients received perioperative information and 9 sessions over 3 days (3 times per day, 20 minutes each) of supervised exercise including core stabilization and motor control training. During the first 4 weeks after surgery they followed conventional postoperative care, and from week 5 they performed home-based core stabilization and motor control training for 8 weeks under therapist supervision by videocall, focused on isometric co-contraction of the abdominal and back muscles	Transversus abdominis activation capacity (Stabilizer Pressure Biofeedback Unit), pre-surgery, immediately after the end of the intervention, and 9 months after the end of intervention (3 and 12 months after surgery)	Patients in G1 obtained significantly better results than G2 in transversus abdomnis activation capacity immediately. There were no between-group differences 9 months after the end of the intervention
Nielsen (2010)	73 patients (G1 = 35, G2 = 38) with degenerative lumbar disease scheduled for surgery; median age (G1 = 48, G2 = 52); female (G1 = 61%, G2 = 59%)	G1: 6–8 weeks of self-directed (30 min each session) preoperative back and abdominal muscle strengthening and aerobic exercises with patient-controlled epidural analgesia, and protein supplementation, followed by 10 sessions over 5 days (twice per day, 30 min each) of immediate postoperative rehabilitation and mobilization	G2: Usual preoperative advice, pain management, and inpatient postoperative rehabilitation. The postoperative program was based on mobilizations once daily (mobilizations on the day of the operation) and performed the general routine rehabilitation for inpatients for 8 days	Lower extremity endurance (5-times sit-to-stand test), the day before surgery, and 1, 3, and 6 months after the end of the intervention and after surgery	No between-group comparisons reported
Oestergaard (2013)	82 patients (G1 = 41, G2 = 41) with degenerative disc disease undergoing lumbar spinal fusion; mean age (G1 = 52.0, G2 = 51.3), female (G1 = 49%, G2 = 59%)	G1: 4 sessions of supervised group rehabilitation including discussion of pain experiences, problems and solutions, doubts about rehabilitation, psychological support, and daily self-directed stability exercises. Additionally, exercise on a bike for warm-up and home exercises including trunk and large muscle group strengthening and stretching exercises, starting 6 weeks after surgery	G2: 4 sessions of supervised group rehabilitation comprising the same components as G1, starting 12 weeks after surgery	Walking capacity (6-minute walk test and Astrand Fitness test), 3, 6, and 12 months after surgery	No between-group differences in any outcome
Sobanski (2025)	60 patients (G1 = 30, G2 = 30) with intervertebral disc injury who had undergone lumbar discectomy; mean age (G1 = 47.0, G2 = 48.0); female (G1 = 60.0%, G2 = 60.0%)	G1: Home-based exercise including breathing exercises, upper and lower limb exercises, and training to stand up and maintain upright posture. From day 15 after surgery and for 3 months, patients received 2 sessions per week (40 minutes each) of kinesitherapy, physical therapy, home exercises, and manual therapy	G2: Home-based exercise including breathing exercises, upper and lower limb exercises, and training to stand up and maintain upright posture. From day 15 after surgery and for 3 months, patients received 2 sessions per week (40 minutes each) of kinesitherapy, physical therapy, and home exercises	Static balance (force plate) immediately after the end of the intervention (3 months after surgery)	G1 obtained significantly better results in some balance outcomes but not others compared to G2
Son (2025)	40 patients (G1 = 20, G2 = 17) with lumbar disc hernation, stenosis, or spondylolisthesis who had undergone lumbar discectomy, laminectomy, or fusion; mean age (G1 = 59.5, G2 = 66.4); female (G1 = 65.0%, G2 = 70.6%).	G1: 24 sessions over 12 weeks (2 sessions per week, 1 hour each) of supervised exercise including stretching, lower limb strength exercises, balance exercises, cardiorespiratory endurance exercise. In addition, one initial education session	G2: 24 sessions over 12 weeks (2 sessions per week, 1 hour each) of self-directed exercise including stretching, lower limb strength exercises, balance exercises, cardiorespiratory endurance exercise. In addition, one initial education session	Trunk flexion and extension strength (dynamometer), trunk flexion and extension endurance (sitting isometric endurance test), walking capacity (6-min walk test), balance (one-leg stance test), immediately after the end of the intervention	G1 obtained significantly better results in walking capacity and balance than G2 No between-group differences were found in trunk flexion and extension strength or endurance
Takenaka (2025)	32 patients (G1 = 13, G2 = 15) with degenerative lumbar spinal stenosis with or without degenerative spondylolisthesis, who had undergone lumbar decompression or decompression and posterior fusion; mean age (G1 = 69.7, G2 = 69.3); female (G1 = 61.5%, G2 = 46.7%)	G1: 1 preoperative in-person educational session (20–30 min, approximately 1 month before surgery) covering lumbar stenosis pathophysiology, recovery expectations, use of walking aids, aerobic and core exercises, health and weight control, and postoperative daily activities plus home exercises with self-tracking	G2: Received the same information as G1 via pamphlet only	Walking capacity (6-min walk test), 1 day before surgery, and 1, 3 and 6 months after surgery	G1 obtained significantly better results in walking capacity than G2 at 3 months after surgery There were no between-group differences 1 day before, 1 month after, or 6 months after surgery
Tegner (2024)	144 patients (G1 = 74, G2 = 70) with lumbar disc degeneration, spondylosis, or spondylolisthesis who had undergone anterior or posterolateral fusion of 1–2 levels; mean age (G1 = 54.9, G2 = 59.4); female (G1 = 52.1%, G2 = 64.7%)	G1: Usual care starting 1–2 weeks before surgery and continued postoperative. Additionally, 9 sessions over 10 weeks of a cognitive treatment approach that included reinforcement of healthy behaviors, identifying negative emotions and beliefs related to pain, and creating and using more adaptive ones, starting 2 days after surgery	G2: usual care starting 1–2 weeks before surgery and continued after surgery. Preoperative usual care was based on information concerning anesthesia, surgery, medication, and an introduction to mobilization techniques. Postoperative usual care was based on information, mobilization, and instructions to gradually increase movement	Physical activity (sedentary time using an accelerometer), 2 weeks and 9.5 months after the end of the intervention (3 and 12 months after surgery)	G1 showed significantly better results in sedentary time compared to G2 at 9.5 months
Yílmaz (2003)	42 patients (G1 = 14, G2 = 14, G3 = 14) with lumbar disc herniation who had undergone microdiscectomy; mean age G1 = 46, G2 = 41, G3 = 43); female (G1 = 43%, G2 = 57%, G3 = 43%)	G1: 24 sessions over 8 weeks (3 sessions a week), of supervised dynamic lumbar stabilization exercise consisting of 3 sets of 5 repetitions of each exercise with an increase in the number of up to 15 repetitions. The treatment was based on flexibility and range of motion exercises and individual dynamic lumbar stabilization exercises, starting approximately 1 month after surgery G2: 24 sessions over 8 weeks (3 sessions a week), of self-directed exercises including lumbar flexion and extension, pelvic tilt, and abdominal strengthening exercises 5 repetitions the first week, 10 the second week, and 15 the third week of each exercise, starting approximately 1 month after surgery	G3: No exercise	Trunk flexion and extension endurance (Ito lumbar trunk muscle endurance test), immediately after the end of intervention (3 months after surgery)	Improved trunk flexion and extension endurance favoring the G1 over G2 and G3 immediately Improved flexion and extension endurance, favoring the G2 over G3 immediately

Abbreviations: G1, group 1; G2, group 2; G3, group 3; min, minutes; PA, physical activity; MVPA, moderate to vigorous physical activity; N/A, not available.

^a^The time specified in the main findings refers to the period following the end of the intervention. If this information is not reported, it refers to the time elapsed since surgery.

### Risk of bias and certainty of evidence

**Fig 2:**
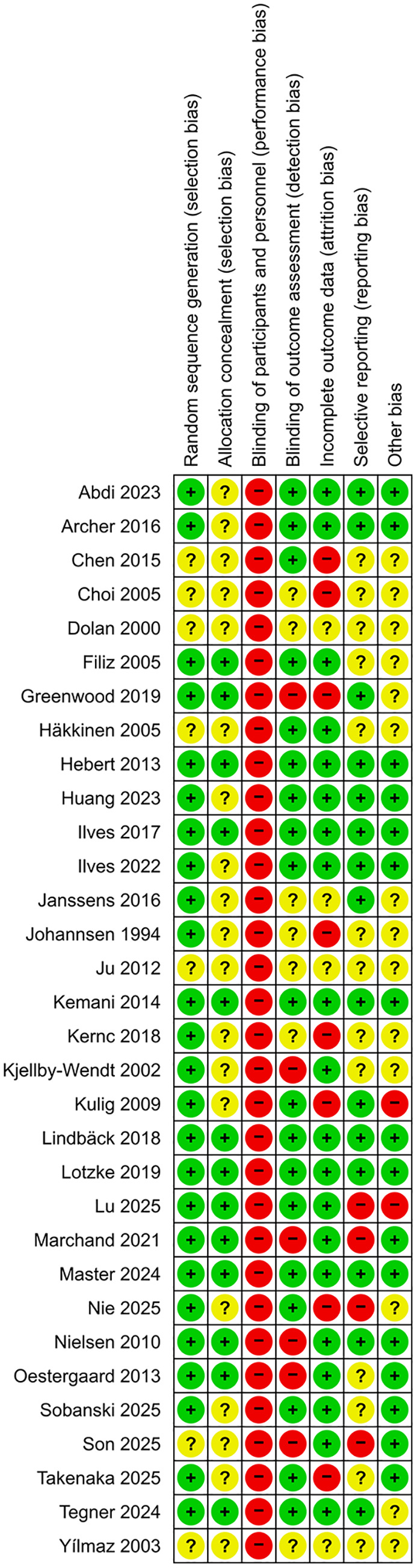
Risk of bias assessment.

**Fig 3:**
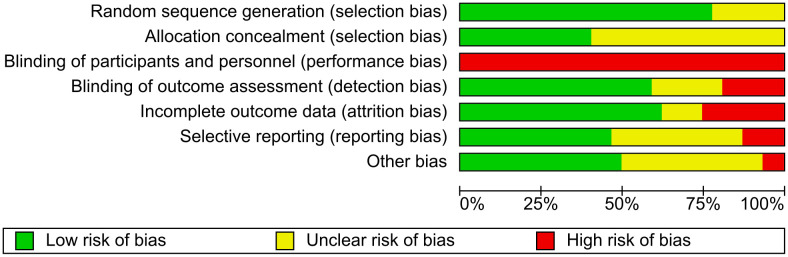
Review authors’ judgements about each risk of bias item, presented as percentages across all included studies.

Six trials were found to have a low risk of bias [33,35,40,44,45,48,50]. All trials were judge to have performance bias, which was expected given the nature of the interventions evaluated in this review, as blinding of the personnel was not feasible. The selection bias (19 trials) and the selective reporting bias (13 trials) domains were both frequently rated as unclear, mainly due to insufficient reporting of these processes in the articles. The ‘other bias’ domain was also commonly rated as unclear (14 trials), mainly owing to a lack of reporting of sample size justification, small sample sizes, or a lack of a conflict-of-interest statement. Detailed information on risk of bias is available in Figs 2 and 3 and S4 File. The overall certainty of evidence ranged from low to moderate, as presented in S5 File.

### Narrative synthesis of study findings

Across the included studies, a wide range of exercise-based, educational, and multimodal interventions were implemented before and after lumbar spine surgery, showing heterogeneous effects on physical capacity and physical activity behavior. Supervised exercise tended to produce greater short-term improvements in trunk strength, endurance, walking capacity, and balance compared to self-directed or usual care, although these effects were not consistent across studies. Psychologically informed rehabilitation and physical activity advice programs occasionally led to improvements in physical activity levels, while prehabilitation generally enhanced pre- and postoperative physical capacity but with mixed results. Overall, the most consistent benefits were observed for supervised, progressive exercise programs. Postoperative interventions were initiated between the first day and three months after surgery, and those started earlier tended to yield better outcomes, although this was not consistent across studies.

### Effectiveness of interventions

#### Exercise versus minimal or usual care.

A total of 12 trials across 13 reports [25,27,29,30,35–37,39,41,43,44,50,56], of which three had a low risk of bias [35,44,50], compared exercise versus minimal or usual care. Several outcomes were reported, including trunk flexion and extension endurance, trunk flexion and extension strength, lateral trunk strength (right and left), walking capacity, walking speed, lower extremity endurance, physical activity levels, and adverse events.

**Fig 4:**
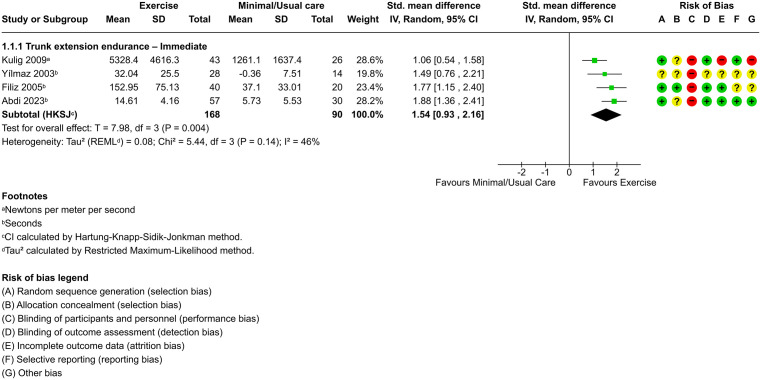
Forest plot of exercise versus minimal/usual care for trunk extension endurance in the immediate term.

Pooled effects from four trials (n = 258), all rated at high risk of bias [25,30,43,56], provided low-certainty evidence that exercise significantly improves trunk extension endurance compared to minimal or usual care immediately (SMD, 1.54; 95% CI, 0.93–2.16; *P* = .004) (Fig 4), with between-study heterogeneity estimated at τ² = 0.08. The prediction interval (95% CI, 0.47–2.62) suggested that future studies would also likely demonstrate a benefit, although the magnitude of effect may vary.

#### Supervised exercise versus self-directed exercise.

A total of eight trials across seven reports [28,30,31,34,38,44,53,56], one with low risk of bias [44], compared supervised exercise versus self-directed exercise on trunk flexion and extension endurance, trunk flexion and extension strength, walking capacity, walking speed, lower extremity endurance, balance, physical activity levels, and adverse events.

**Fig 5:**
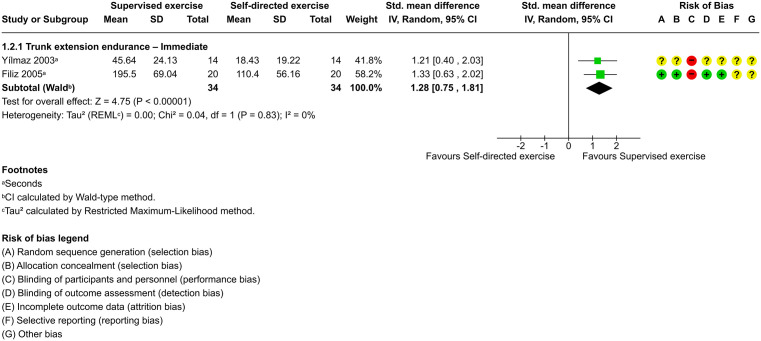
Forest plot of supervised exercise versus self-directed exercise for trunk extension endurance in the immediate term.

Pooled effects from two trials (n = 68) [30,56], both exhibiting high risk of bias, provided low-certainty evidence that supervised exercise improves trunk extension endurance compared to self-directed exercise in the immediate term (SMD, 1.28; 95% CI, 0.75–1.81; *P* < .00001) (Fig 5) with no heterogeneity (τ² = 0.00). Although the prediction interval (95% CI, 0.75–1.81) suggests statistical consistency across the included studies, this estimate should be interpreted with caution given the small number of trials and their high risk of bias, and future studies may yield different effect sizes.

#### Psychologically informed rehabilitation versus minimal or usual care.

Five trials across six reports [26,40,44,45,48,55], of which four had a low risk of bias [40,44,45,48,55], compared psychologically informed rehabilitation versus minimal or usual care on walking capacity, walking speed, lower extremity endurance, physical activity levels, and adverse events.

**Fig 6:**
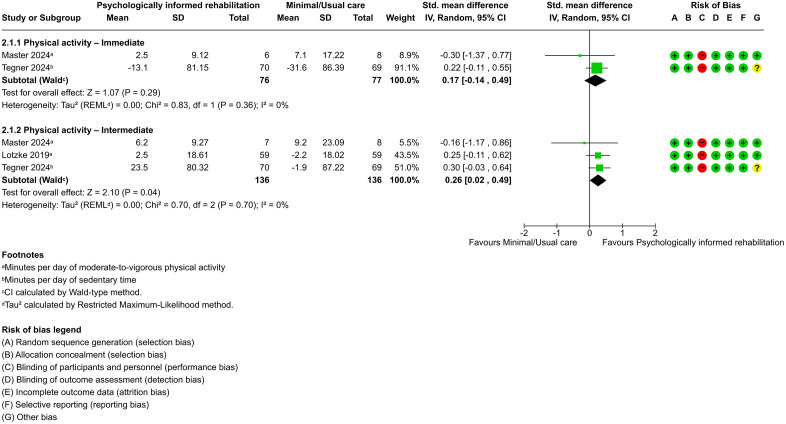
Forest plot of psychologically informed rehabilitation versus minimal/usual care for physical activity at immediate and intermediate terms.

Pooled effects from two trials (n = 157) [48,55], with one trial exhibiting low risk of bias [48], provided low-certainty evidence that psychologically informed rehabilitation does not significantly increase physical activity levels in comparison to minimal or usual care immediately (SMD, 0.17; 95% CI, −0.14 to 0.49; *P* = .29) (Fig 6) with no heterogeneity (τ² = 0.00). The prediction interval suggested that the effect in future studies would likely fall within the same range as the confidence interval (95% CI, −0.14 to 0.49). At the intermediate time point, pooled effects from three trials (n = 272) [45,48,55], two of which had low risk of bias [45,48], provided low-certainty evidence that psychologically informed rehabilitation significantly increases physical activity levels in comparison to minimal or usual care (SMD, 0.26; 95% CI, 0.02–0.49; *P* = .04) (Fig 6) with no heterogeneity (τ² = 0.00). The prediction interval (95% CI, 0.02–0.49) suggested that the effect in future studies would likely remain within the same range, although it could be small and of uncertain clinical relevance.

#### Physical activity advice versus minimal or usual care.

Five trials across seven reports [35,36,40,42,44,45,48], of which five reports were found to have a low risk of bias [35,40,44,45,48], compared physical activity advice versus minimal or usual care on trunk extension endurance, trunk flexion and extension strength, walking capacity, walking speed, physical activity levels, and adverse events.

**Fig 7:**
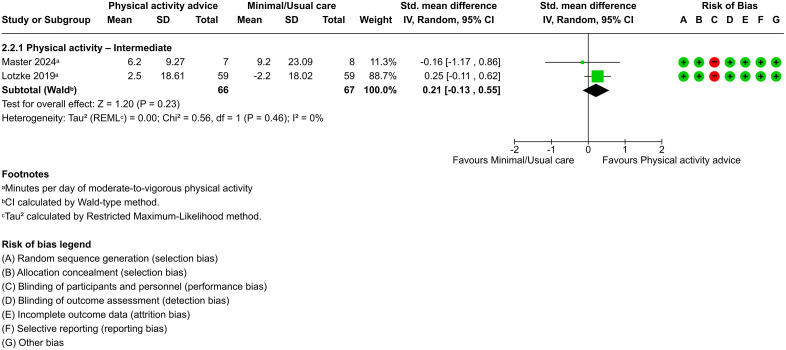
Forest plot of physical activity advice versus minimal/usual care for physical activity at the intermediate term.

Pooled effects from two trials (n = 133) [45,48] both with low risk of bias, provided moderate-certainty evidence that physical activity advice does not significantly increase physical activity levels compared to minimal or usual care at the intermediate time point (SMD, 0.21; 95% CI, −0.13 to 0.55; *P* = .23) (Fig 7), with no heterogeneity (τ² = 0.00). The prediction interval (95% CI, −0.13 to 0.55) suggested that future studies would likely report effects within a similar range.

#### Prehabilitation versus minimal or usual care.

Six trials across seven reports [27,40,44,45,47,50,55], of which four were found to have a low risk of bias [40,44,45,50,55], compared prehabilitation versus minimal or usual care in trunk extension endurance, trunk flexion and extension strength, walking capacity, walking speed, lower extremity endurance, lower extremity strength, physical activity levels, and adverse events.

**Fig 8:**
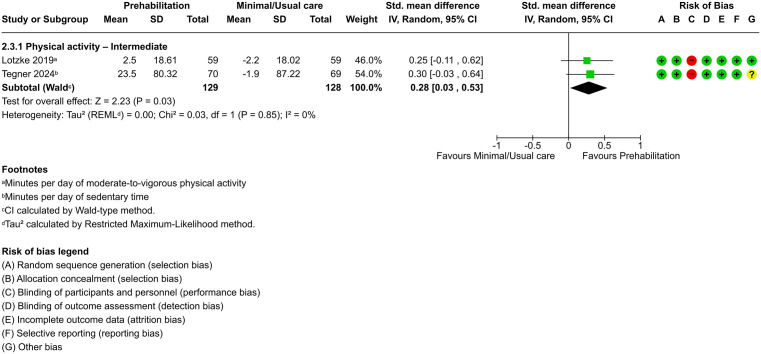
Forest plot of prehabilitation versus minimal/usual care for physical activity at the intermediate term.

Pooled effects from two trials (n = 257) [45,55], with one trial exhibiting low risk of bias [45], provided low-certainty evidence that prehabilitation significantly increases physical activity levels compared to minimal or usual care at the intermediate time point (SMD, 0.28; 95% CI, 0.03–0.53; *P* = .03) (Fig 8), with no heterogeneity (τ² = 0.00). The prediction interval (95% CI, 0.03–0.53) suggested that the effect in future studies would likely remain within the same range, although it could be small and of uncertain clinical relevance.

## Discussion

The findings of this systematic review provide low-to-moderate-certainty evidence that rehabilitation and behavior change interventions may positively affect physical capacity in the immediate term and physical activity behavior in the intermediate term among patients with lumbar degenerative disc disease who have undergone lumbar surgery.

Across the 32 reports included in this review, rehabilitation and behavior change interventions varied substantially in content, timing, and delivery, likely reflecting the absence of international recommendations for structured rehabilitation before or after lumbar spine surgery. Exercise-based programs were the most frequently studied and generally improved physical capacity, particularly trunk strength and endurance, consistent with their emphasis on these components. These findings support the preference for progressive and supervised exercise, which may enhance adherence, ensure adequate intensity, and facilitate safe progression. Psychologically informed rehabilitation interventions showed mixed effects on physical activity behavior. The variability across studies suggests that the duration and intensity of these programs may be critical for achieving meaningful behavioral changes, highlighting the importance of sustained and structured approaches to promote long-term engagement and obtain intermediate-term benefits. Physical activity advice interventions did not demonstrate consistent improvements in physical activity levels, with considerable heterogeneity between studies. Finally, prehabilitation appeared to play a positive role in improving both pre- and postoperative physical capacity, as well as postoperative physical activity levels. These findings suggest that optimizing patients’ functional status before surgery may facilitate recovery and promote earlier return to activity.

In the comparison between exercise and minimal or usual care for improving physical capacity and physical activity behavior, there was only enough research to pool results for trunk extension endurance in the immediate term. In this outcome, exercise interventions consistently resulted in more meaningful benefits than minimal or usual care. The three trials with the largest effect sizes implemented 24-session programs initiated 4–6 weeks postoperative [25,30,56], which may represent a minimum threshold for intensity, frequency, and timing. These programs typically included lumbar and abdominal strengthening, stretching, range of motion, and pelvic tilt exercises. However, the certainty of this evidence remains low due to methodological limitations, including high risk of bias and small sample sizes in the included studies. Despite these issues, the results reinforce the role of structured exercise in rehabilitation. Previous reviews support the superiority of exercise compared to usual care for improving pain and function after disc herniation surgery, with certainty of evidence ranging from very low to moderate [57,58]. In patients with lumbar degenerative conditions, low-certainty evidence supports that exercise improves disability [59]. Similarly, in individuals undergoing lumbar discectomy, one review suggests that exercise may improve lumbar extension strength [60].

In the comparison between supervised exercise and self-directed exercise for improving physical capacity and physical activity behavior, there was only enough research to pool results for trunk extension endurance in the immediate term. For this specific outcome, supervised exercise appeared to result in significantly greater improvements than self-directed exercise. Both trials demonstrated improved results with supervised exercise, which involved 24 sessions over 8 weeks, starting 1 month postoperative [30,56]. This structured approach may contribute to the standardization of intervention intensity, duration, and timing. The supervised exercise programs differed from the self-directed exercise programs in two key aspects: the presence of supervision and the inclusion of lumbar stabilization exercises. Both factors may potentially explain the greater improvements in trunk extension endurance observed with supervised exercise. However, the certainty of this evidence was low due to methodological limitations, such as a high risk of bias and small sample sizes in these studies. Other reviews comparing supervised and self-directed exercise for pain and function yielded mixed results. One review provided very low-certainty evidence suggesting no difference between the interventions in patients undergoing lumbar disc surgery [57], while another study offered low-certainty evidence indicating that supervised exercise reduces pain and disability after lumbar surgery [61]. A further review concluded that the role of supervision in postoperative rehabilitation remains unclear, with conflicting results across studies [62].

In the comparison between psychologically informed rehabilitation and minimal or usual care for improving physical capacity and physical activity behavior, only data on physical activity levels were sufficient to allow pooling of results in the immediate and intermediate term. In the immediate term, no clear differences between interventions were found, as the two included trials reported inconsistent findings [48,55]. Given that the available evidence is limited and inconsistent, with both trials reporting conflicting results, and the prediction interval overlapping the null effect, there is uncertainty about the true effect in future studies. Therefore, additional well-designed trials are needed to clarify the immediate effects of psychologically informed rehabilitation on physical activity levels. However, in the intermediate term, significant effects favored psychologically informed rehabilitation. This delayed benefit may reflect the time needed for psychologically informed strategies to influence physical activity levels, or it may result from insufficient statistical power in the immediate term analysis. The trial reporting non-significant results favoring minimal or usual care implemented a less comprehensive intervention [48], potentially underestimating the true effect. In contrast, the two trials with more favorable results for psychologically informed rehabilitation incorporated cognitive behavioral strategies, encouraged physical activity despite pain, addressed pain-related beliefs, and provided spinal health education, initiated before surgery [45,55]. The consistency of these components across the effective interventions may help establish a standard for such interventions. However, these conclusions are limited by the low-certainty evidence and the small number of trials. Additionally, a meta-analysis showed that cognitive behavioral therapy improves quality of life in patients with lumbar spinal surgery [63].

In the comparison between physical activity advice and minimal or usual care for improving physical capacity and physical activity behavior, only data on physical activity levels were sufficient to allow pooling of results in the intermediate term. In this outcome, no significant differences were found between the interventions. The two included trials reported contradictory results, which may be partly explained by differences in intervention quality and intensity [45,48]. The trial favoring minimal or usual care implemented a less intensive or incomplete intervention [48], whereas the trial favoring physical activity advice provided a more comprehensive approach, initiated before surgery and including cognitive behavioral strategies [45], which may have contributed to the observed benefits. Overall, these findings suggest that physical activity advice may have limited effects on postoperative physical activity levels in this population during the intermediate term, based on moderate-certainty evidence. Given that the available evidence is limited and inconsistent, with both trials reporting conflicting results, and the prediction interval including the null effect, there is uncertainty about the true effect in future studies. Consequently, further well-designed trials are needed to clarify the intermediate effects of physical activity advice on physical activity levels. Evidence from other populations indicates that improvements in physical activity levels may require longer follow-up periods or more intensive interventions. For example, a separate meta-analysis found that wearable activity trackers can promote physical activity engagement and reduce sedentary behavior in hospitalized patients, although it did not report the timing or duration of follow-up [64]. Additionally, a systematic review involving patients with various health conditions reported long-term improvements in physical activity levels after 24 months of physical activity advice interventions, suggesting that longer follow-up may be necessary to detect meaningful changes [65].

In the comparison between prehabilitation and minimal or usual care for improving physical capacity and physical activity behavior, only data on physical activity levels were sufficient to allow pooling of results in the intermediate term. In this analysis, prehabilitation interventions showed better results. The consistency between trials suggests that prehabilitation could play an important role when it incorporates components such as cognitive behavioral strategies, encouragement of physical activity despite pain, addressing pain-related beliefs, and spinal health education in improving physical activity levels [45,55]. However, caution is warranted due to the low-certainty evidence due to the high risk of bias and small number of patients. Evidence from other surgical contexts has shown inconsistent findings. A meta-analysis including only two trials in non-spine surgery populations found no significant benefits of physical activity interventions on physical activity levels when initiated before surgery [66]. Similarly, a small systematic review did not identify clear effects of prehabilitation on physical activity levels in the pre- or postoperative period among patients undergoing various musculoskeletal and visceral surgeries [67].

To our knowledge, no previous systematic reviews or meta-analyses in this population have included the same comparisons between interventions and control groups as those presented in this study, nor have they evaluated the specific outcomes examined in the current study.

This systematic review provides relevant insights for clinicians by synthesizing the current evidence on rehabilitation interventions surrounding lumbar spine surgery. However, the substantial heterogeneity in intervention types, components, timing, and outcomes limits the ability to establish standardized clinical protocols. Rather than prescribing specific interventions, the findings may help inform clinical decision-making by highlighting areas where evidence appears more consistent, such as the potential short-term benefits of supervised and progressive exercise programs for physical capacity outcomes.

Importantly, the findings of this review should be interpreted in the context of individual patient characteristics, preferences, and rehabilitation needs. Given the diversity of interventions and outcomes, different approaches may be appropriate for different patient profiles. For example, psychologically informed rehabilitation may be particularly relevant for patients presenting with maladaptive pain beliefs or fear-avoidance behaviors, whereas structured physical rehabilitation programs may be more suitable for individuals with marked physical deconditioning but more adaptive pain-related beliefs. The differentiated time-point analyses further suggest that improvements in physical activity levels may require longer follow-up periods, whereas changes in physical capacity outcomes, such as trunk extension endurance, may be observed earlier.

At a broader level, while this review may help identify areas where evidence is more consistent, its findings should be interpreted cautiously by policymakers. Important factors such as intervention costs, required professional competencies, availability of specialized personnel, organizational constraints, and feasibility of early postoperative implementation were not assessed and may substantially influence real-world applicability. These considerations highlight the need for future studies that integrate effectiveness outcomes with economic evaluations and implementation-related factors to better inform policy and health system decision-making.

From a patient-centered perspective, the clinical relevance of the outcomes assessed in the included studies should be interpreted with caution. Several commonly reported physical capacity outcomes, such as trunk strength and endurance, are not included in established core outcome sets for low back pain, including those proposed by the International Consortium for Health Outcomes Measurement [68]. Although these measures may reflect improvements in specific physiological capacities targeted by rehabilitation programs, their direct translation into meaningful functional recovery or daily-life participation is uncertain. In contrast, outcomes such as physical activity levels and walking capacity may better reflect patient-relevant improvements, as they are more closely linked to everyday functioning and overall health. The limited and heterogeneous evidence for these outcomes highlights an important gap in the literature and underscores the need for future trials to prioritize patient-relevant outcomes alongside traditional measures of physical capacity.

Future studies should prioritize the design and implementation of high-quality RCTs with minimized risk of bias, following the CONSORT guidelines [69]. Emphasis should be placed on ensuring transparent reporting of randomization and allocation processes, as well as proper blinding of outcome. These trials should also include larger sample sizes to enhance statistical power and improve generalizability. Standardizing outcome measures, including reliable and valid instruments specific to the target population, and objective assessments when possible, would enhance comparability and reduce heterogeneity. For example, trunk extension endurance can be reliably and precisely evaluated using an isokinetic dynamometer [70], while physical activity behavior is best measured objectively through accelerometers rather than relying on subjective questionnaires [71]. Additionally, incorporating multiple time points for outcome assessment is also essential to accurately capture the immediate, short, intermediate, and long-term effects of interventions.

### Strengths and limitations

This study has several notable strengths and limitations. Comprehensive manual and electronic searches were conducted, and a priori study protocol registration was completed. Two review authors independently screened the studies, performed data extraction, data synthesis, data analysis, and risk of bias assessment, and evaluated the certainty of the evidence. Furthermore, standard statistical methods were applied, and the most current guidelines for conducting and reporting systematic reviews were followed. Notably, this is the first meta-analysis to include trunk extension endurance and physical activity levels in this patient population, offering novel insights into previously unexamined outcomes. However, there are several limitations to consider. The ability to perform a comprehensive meta-analysis was restricted due to the limited number of available clinical trials. Additionally, the high risk of bias in most of the studies and the low certainty of the evidence reduce the robustness of the study’s conclusions. The considerable heterogeneity observed among the studies and differences in the tools used to measure outcomes hindered the ability to effectively pool the data for meaningful comparisons.

Furthermore, the comparator described as “usual or minimal care” is often not clearly defined in many trials. Additionally, the content of usual care varies considerably across clinics and rehabilitation centers [72], introducing unmeasured confounding and increasing heterogeneity among trials that used “usual or minimal care” as the control group in randomized studies.

## Conclusions

In adults with lumbar degenerative disease who have undergone lumbar surgery, exercise appears to be effective in improving lumbar extension endurance in the immediate term, with supervised programs outperforming self-directed approaches. Psychologically informed rehabilitation and prehabilitation interventions increased physical activity levels in the intermediate term, whereas physical activity advice did not. However, study findings should be interpreted with caution due to the low certainty of the evidence and the high risk of bias in many of the included studies. Future research should prioritize the identification of patient-relevant outcomes and clinically meaningful subgroups to better align rehabilitation strategies with patient needs, before advancing to larger, high-quality trials.

## Supporting information

S1 Table PRISMA checklist. (DOCX)

S1 File Definitions of interventions. (DOCX)

S2 File Search strategies applied to EMBASE, MEDLINE, PsycINFO and CENTRAL. (DOCX)

S2 Table Reasons for study exclusion. (DOCX)

S3 File Contrast tables. (DOCX)

S4 File Risk of bias tables. (DOCX)

S5 File GRADE tables. (DOCX)

## Abbreviations

CI: confidence interval
GRADE: Grading of Recommendations, Assessment, Development and Evaluation
MVPA: moderate-to-vigorous physical activity
PRISMA: Preferred Reporting Items for Systematic Reviews and Meta-Analyses
RCT: randomized controlled trial
SMD: standardized mean difference

## References

[1] Teraguchi M, Yoshimura N, Hashizume H, Muraki S, Yamada H, Minamide A, et al. Prevalence and distribution of intervertebral disc degeneration over the entire spine in a population-based cohort: the Wakayama Spine Study. Osteoarthritis Cartilage. 2014;22(1):104–10. doi: 10.1016/j.joca.2013.10.019 24239943

[2] Ravindra VM, Senglaub SS, Rattani A, Dewan MC, Härtl R, Bisson E. Degenerative lumbar spine disease: estimating global incidence and worldwide volume. Glob Spine J. 2018;8:784–94. doi: 10.1177/2192568218770769 PMC6293435 30560029

[3] Parenteau CS, Lau EC, Campbell IC, Courtney A. Prevalence of spine degeneration diagnosis by type, age, gender, and obesity using Medicare data. Sci Rep. 2021;11(1):5389. doi: 10.1038/s41598-021-84724-6 33686128 PMC7940625

[4] Ferreira ML, de Luca K, Haile LM, Steinmetz JD, Culbreth GT, Cross M, et al. Global, regional, and national burden of low back pain, 1990–2020, its attributable risk factors, and projections to 2050: a systematic analysis of the Global Burden of Disease Study 2021. Lancet Rheumatol. 2023;5:e316–29. doi: 10.1016/S2665-9913(23)00098-X PMC10234592 37273833

[5] Zigler J, Ferko N, Cameron C, Patel L. Comparison of therapies in lumbar degenerative disc disease: a network meta-analysis of randomized controlled trials. J Comp Eff Res. 2018;7(3):233–46. doi: 10.2217/cer-2017-0047 29542364

[6] Van Isseldyk F, Padilla-Lichtenberger F, Guiroy A, Asghar J, Quillo-Olvera J, Quillo-Reséndiz J, et al. Endoscopic Treatment of Lumbar Degenerative Disc Disease: A Narrative Review of Full-Endoscopic and Unilateral Biportal Endoscopic Spine Surgery. World Neurosurg. 2024;188:e93–107. doi: 10.1016/j.wneu.2024.05.047 38754549

[7] Lee YC, Zotti MGT, Osti OL. Operative management of lumbar degenerative disc disease. Asian Spine J. 2016;10:801–19. doi: 10.4184/asj.2016.10.4.801 27559465 PMC4995268

[8] Dupeyron A, Ribinik P, Rannou F, Kabani S, Demoulin C, Dufour X, et al. Rehabilitation and lumbar surgery: the French recommendations for clinical practice. Ann Phys Rehabil Med. 2021;64(6):101548. doi: 10.1016/j.rehab.2021.101548 34192564

[9] Skarsgard M, Almojuela A, Gagliardi M, Swamy G, Nicholls F, Jacobs WB, et al. Interventions to Modify Psychological Processes in Patients Undergoing Spine Surgery: A Systematic Review. Global Spine J. 2025;15(5):2798–809. doi: 10.1177/21925682251318958 39918081 PMC11806454

[10] Page MJ, McKenzie JE, Bossuyt PM, Boutron I, Hoffmann TC, Mulrow CD, et al. The PRISMA 2020 statement: an updated guideline for reporting systematic reviews. BMJ. 2021;372:n71. doi: 10.1136/bmj.n71 33782057 PMC8005924

[11] Lefebvre C, Glanville J, Briscoe S, Featherstone R, Littlewood A, Metzendorf MI. Searching for and selecting studies. Cochrane Handbook for Systematic Reviews of Interventions. 2024.

[12] Higgins JPT, Altman DG, Gøtzsche PC, Jüni P, Moher D, Oxman AD, et al. The Cochrane Collaboration’s tool for assessing risk of bias in randomised trials. BMJ. 2011;343:d5928. doi: 10.1136/bmj.d5928 22008217 PMC3196245

[13] Borenstein M, Hedges L, Higgins JPT, Rothstein H. Introduction to Meta-Analysis. Wiley. 2009.

[14] Review Manager (RevMan). The Cochrane Collaboration. 2024.

[15] Higgins JP, Li T, Deeks JJ. Choosing effect measures and computing estimates of effect. Cochrane Handbook for Systematic Reviews of Interventions. Wiley. 2019. 143–76. doi: 10.1002/9781119536604.ch6

[16] Hartung J, Knapp G. A refined method for the meta-analysis of controlled clinical trials with binary outcome. Stat Med. 2001;20(24):3875–89. doi: 10.1002/sim.1009 11782040

[17] Deeks J, Higgins J, Altman DG, McKenzie J, Veroniki A. Analysing data and undertaking meta-analyses. Cochrane Handbook for Systematic Reviews of Interventions. Cochrane. 2024.

[18] Higgins JPT, Thompson SG, Spiegelhalter DJ. A re-evaluation of random-effects meta-analysis. J R Stat Soc Ser A Stat Soc. 2009;172(1):137–59. doi: 10.1111/j.1467-985X.2008.00552.x 19381330 PMC2667312

[19] Harville DA. Maximum likelihood approaches to variance component estimation and to related problems. J Am Stat Assoc. 1977;72:320–38. doi: doi/abs/10.1080/01621459.1977.10480998

[20] Higgins JPT, Thompson SG, Deeks JJ, Altman DG. Measuring inconsistency in meta-analyses. BMJ. 2003;327(7414):557–60. doi: 10.1136/bmj.327.7414.557 12958120 PMC192859

[21] Egger M, Davey Smith G, Schneider M, Minder C. Bias in meta-analysis detected by a simple, graphical test. BMJ. 1997;315(7109):629–34. doi: 10.1136/bmj.315.7109.629 9310563 PMC2127453

[22] Guyatt GH, Oxman AD, Vist GE, Kunz R, Falck-Ytter Y, Alonso-Coello P, et al. GRADE: an emerging consensus on rating quality of evidence and strength of recommendations. BMJ. 2008;336(7650):924–6. doi: 10.1136/bmj.39489.470347.AD 18436948 PMC2335261

[23] Evidence P. GRADEpro GDT. Hamilton (ON): McMaster University.

[24] Guyatt GH, Oxman AD, Kunz R, Brozek J, Alonso-Coello P, Rind D, et al. GRADE guidelines 6. Rating the quality of evidence--imprecision. J Clin Epidemiol. 2011;64(12):1283–93. doi: 10.1016/j.jclinepi.2011.01.012 21839614

[25] Abdi A, Bagheri SR, Shekarbeigi Z, Usefvand S, Alimohammadi E. The effect of repeated flexion-based exercises versus extension-based exercises on the clinical outcomes of patients with lumbar disk herniation surgery: a randomized clinical trial. Neurol Res. 2023;45(1):28–40. doi: 10.1080/01616412.2022.2116686 36039973

[26] Archer KR, Devin CJ, Vanston SW, Koyama T, Phillips SE, Mathis SL, et al. Cognitive-Behavioral-Based Physical Therapy for Patients With Chronic Pain Undergoing Lumbar Spine Surgery: A Randomized Controlled Trial. J Pain. 2016;17(1):76–89. doi: 10.1016/j.jpain.2015.09.013 26476267 PMC4709178

[27] Chen C-Y, Chang C-W, Lee S-T, Chen Y-C, Tang SF-T, Cheng C-H, et al. Is rehabilitation intervention during hospitalization enough for functional improvements in patients undergoing lumbar decompression surgery? A prospective randomized controlled study. Clin Neurol Neurosurg. 2015;129 Suppl 1:S41-6. doi: 10.1016/S0303-8467(15)30011-1 25683312

[28] Choi G, Raiturker PP, Kim M-J, Chung DJ, Chae Y-S, Lee S-H. The effect of early isolated lumbar extension exercise program for patients with herniated disc undergoing lumbar discectomy. Neurosurgery. 2005;57(4):764–72; discussion 764-72. doi: 10.1093/neurosurgery/57.4.764 16239890

[29] Dolan P, Greenfield K, Nelson RJ, Nelson IW. Can exercise therapy improve the outcome of microdiscectomy?. Spine (Phila Pa 1976). 2000;25(12):1523–32. doi: 10.1097/00007632-200006150-00011 10851101

[30] Filiz M, Cakmak A, Ozcan E. The effectiveness of exercise programmes after lumbar disc surgery: a randomized controlled study. Clin Rehabil. 2005;19(1):4–11. doi: 10.1191/0269215505cr836oa 15704503

[31] Greenwood J, McGregor A, Jones F, Hurley M. Rehabilitation following lumbar fusion surgery (REFS) a randomised controlled feasibility study. Eur Spine J. 2019;28(4):735–44. doi: 10.1007/s00586-019-05913-6 30788599

[32] Häkkinen A, Ylinen J, Kautiainen H, Tarvainen U, Kiviranta I. Effects of home strength training and stretching versus stretching alone after lumbar disk surgery: a randomized study with a 1-year follow-up. Arch Phys Med Rehabil. 2005;86(5):865–70. doi: 10.1016/j.apmr.2004.11.012 15895329

[33] Hebert JJ, Fritz JM, Thackeray A, Koppenhaver SL, Teyhen D. Early multimodal rehabilitation following lumbar disc surgery: a randomised clinical trial comparing the effects of two exercise programmes on clinical outcome and lumbar multifidus muscle function. Br J Sports Med. 2015;49(2):100–6. doi: 10.1136/bjsports-2013-092402 24029724

[34] Huang A-H, Chou W-H, Wang WT-J, Chen W-Y, Shih Y-F. Effects of early aquatic exercise intervention on trunk strength and functional recovery of patients with lumbar fusion: a randomized controlled trial. Sci Rep. 2023;13(1):10716. doi: 10.1038/s41598-023-37237-3 37400496 PMC10317955

[35] Ilves O, Häkkinen A, Dekker J, Wahlman M, Tarnanen S, Pekkanen L, et al. Effectiveness of postoperative home-exercise compared with usual care on kinesiophobia and physical activity in spondylolisthesis: A randomized controlled trial. J Rehabil Med. 2017;49(9):751–7. doi: 10.2340/16501977-2268 28862315

[36] Ilves O, Neva MH, Häkkinen K, Dekker J, Järvenpää S, Kyrölä K, et al. Effectiveness of a 12-month home-based exercise program on trunk muscle strength and spine function after lumbar spine fusion surgery: a randomized controlled trial. Disabil Rehabil. 2022;44(4):549–57. doi: 10.1080/09638288.2020.1772383 32525413

[37] Janssens L, Brumagne S, Claeys K, Pijnenburg M, Goossens N, Rummens S, et al. Proprioceptive use and sit-to-stand-to-sit after lumbar microdiscectomy: The effect of surgical approach and early physiotherapy. Clin Biomech (Bristol). 2016;32:40–8. doi: 10.1016/j.clinbiomech.2015.12.011 26795132

[38] Johannsen F, Remvig L, Kryger P, Beck P, Lybeck K, Larsen LH, et al. Supervised endurance exercise training compared to home training after first lumbar diskectomy: a clinical trial. Clin Exp Rheumatol. 1994;12(6):609–14. 7895394

[39] Ju S, Park G, Kim E. Effects of an exercise treatment program on lumbar extensor muscle strength and pain of rehabilitation patients recovering from lumbar disc herniation surgery. J Phys Ther Sci. 2012;24:515–8. doi: 10.1589/jpts.24.515

[40] Kemani MK, Hanafi R, Brisby H, Lotzke H, Lundberg M. Long-Term Follow-Up of a Person-Centered Prehabilitation Program Based on Cognitive-Behavioral Physical Therapy for Patients Scheduled for Lumbar Fusion. Phys Ther. 2024;104(8):pzae069. doi: 10.1093/ptj/pzae069 38753831 PMC11913609

[41] Kernc D, Strojnik V, Vengust R. Early initiation of a strength training based rehabilitation after lumbar spine fusion improves core muscle strength: a randomized controlled trial. J Orthop Surg Res. 2018;13(1):151. doi: 10.1186/s13018-018-0853-7 29914580 PMC6006840

[42] Kjellby-Wendt G, Carlsson SG, Styf J. Results of early active rehabilitation 5-7 years after surgical treatment for lumbar disc herniation. J Spinal Disord Tech. 2002;15(5):404–9. doi: 10.1097/00024720-200210000-00010 12394665

[43] Kulig K, Beneck GJ, Selkowitz DM, Popovich JM Jr, Ge TT, Flanagan SP, et al. An intensive, progressive exercise program reduces disability and improves functional performance in patients after single-level lumbar microdiskectomy. Phys Ther. 2009;89(11):1145–57. doi: 10.2522/ptj.20080052 19778981

[44] Lindbäck Y, Tropp H, Enthoven P, Abbott A, Öberg B. PREPARE: presurgery physiotherapy for patients with degenerative lumbar spine disorder: a randomized controlled trial. Spine J. 2018;18(8):1347–55. doi: 10.1016/j.spinee.2017.12.009 29253630

[45] Lotzke H, Brisby H, Gutke A, Hägg O, Jakobsson M, Smeets R, et al. A Person-Centered Prehabilitation Program Based on Cognitive-Behavioral Physical Therapy for Patients Scheduled for Lumbar Fusion Surgery: A Randomized Controlled Trial. Phys Ther. 2019;99(8):1069–88. doi: 10.1093/ptj/pzz020 30951604 PMC6665875

[46] Lu H, Shen Y, Shao Q, Huang Z, Cao Y, Su J, et al. Early functional training is not superior to routine rehabilitation in improving walking distance and multifidus atrophy after lumbar fusion: a randomized controlled trial with 6-month follow-up. Eur Spine J. 2025;34(6):2453–66. doi: 10.1007/s00586-025-08771-7 40249395

[47] Marchand A-A, Houle M, O’Shaughnessy J, Châtillon C-É, Cantin V, Descarreaux M. Effectiveness of an exercise-based prehabilitation program for patients awaiting surgery for lumbar spinal stenosis: a randomized clinical trial. Sci Rep. 2021;11(1):11080. doi: 10.1038/s41598-021-90537-4 34040109 PMC8155114

[48] Master H, Coronado RA, Whitaker S, Block S, Vanston SW, Pennings JS, et al. Combining Wearable Technology and Telehealth Counseling for Rehabilitation After Lumbar Spine Surgery: Feasibility and Acceptability of a Physical Activity Intervention. Phys Ther. 2024;104(2):pzad096. doi: 10.1093/ptj/pzad096 37478463 PMC10851843

[49] Nie C, Chen K, Huang M, Zhu Y, Jiang J, Xia X, et al. Postoperative early initiation of sequential exercise program in preventing persistent spinal pain syndrome type-2 after modified transforaminal lumbar interbody fusion: a prospective randomized controlled trial. Eur Spine J. 2025;34(1):191–203. doi: 10.1007/s00586-024-08541-x 39453543

[50] Nielsen PR, Jørgensen LD, Dahl B, Pedersen T, Tønnesen H. Prehabilitation and early rehabilitation after spinal surgery: randomized clinical trial. Clin Rehabil. 2010;24(2):137–48. doi: 10.1177/0269215509347432 20103575

[51] Oestergaard LG, Nielsen CV, Bünger CE, Svidt K, Christensen FB. The effect of timing of rehabilitation on physical performance after lumbar spinal fusion: a randomized clinical study. Eur Spine J. 2013;22(8):1884–90. doi: 10.1007/s00586-013-2717-5 23563500 PMC3731483

[52] Sobański G, Wolan-Nieroda A, Guzik A, Maciejczak A. Change in patients’ psychophysical performance following lumbar discectomy relative to the postoperative rehabilitation programme. Acta Bioeng Biomech. 2025;27(1):119–30. doi: 10.37190/abb-02558-2024-03 40544463

[53] Son S, Park HB, Kong KS, Yoo BR, Kim WK, Sim JA. Comparison of Guided Exercise and Self-Paced Exercise After Lumbar Spine Surgery: A Randomized Controlled Trial. Life (Basel). 2025;15(7):1070. doi: 10.3390/life15071070 40724572 PMC12298251

[54] Takenaka H, Kamiya M, Suzuki J. Prehabilitation Improves Early Outcomes in Lumbar Spinal Stenosis Surgery: A Pilot Randomized Controlled Trial. Clin Spine Surg. 2025;38(10):E480–7. doi: 10.1097/BSD.0000000000001779 40035543

[55] Tegner H, Rolving N, Henriksen M, Bech-Azeddine R, Lundberg M, Esbensen BA. The Effect of Graded Activity and Pain Education After Lumbar Spinal Fusion on Sedentary Behavior 3 and 12 Months Postsurgery: A Randomized Controlled Trial. Arch Phys Med Rehabil. 2024;105(8):1480–9. doi: 10.1016/j.apmr.2024.04.005 38685291

[56] Yílmaz F, Yílmaz A, Merdol F, Parlar D, Sahin F, Kuran B. Efficacy of dynamic lumbar stabilization exercise in lumbar microdiscectomy. J Rehabil Med. 2003;35(4):163–7. doi: 10.1080/16501970306125 12892241

[57] Oosterhuis T, Costa LOP, Maher CG, de Vet HCW, van Tulder MW, Ostelo RWJG. Rehabilitation after lumbar disc surgery. Cochrane Database Syst Rev. 2014;2014(3):CD003007. doi: 10.1002/14651858.CD003007.pub3 24627325 PMC7138272

[58] Yu H, Cancelliere C, Mior S, Pereira P, Nordin M, Brunton G, et al. Effectiveness of postsurgical rehabilitation following lumbar disc herniation surgery: A systematic review. Brain Spine. 2024;4:102806. doi: 10.1016/j.bas.2024.102806 38690091 PMC11059472

[59] Bogaert L, Thys T, Depreitere B, Dankaerts W, Amerijckx C, Van Wambeke P, et al. Rehabilitation to improve outcomes of lumbar fusion surgery: a systematic review with meta-analysis. Eur Spine J. 2022;31(6):1525–45. doi: 10.1007/s00586-022-07158-2 35258644

[60] Atsidakou N, Matsi AE, Christakou A. The effectiveness of exercise program after lumbar discectomy surgery. J Clin Orthop Trauma. 2021;16:99–105. doi: 10.1016/j.jcot.2020.12.030 33680831 PMC7919938

[61] Manni T, Ferri N, Vanti C, Ferrari S, Cuoghi I, Gaeta C, et al. Rehabilitation after lumbar spine surgery in adults: a systematic review with meta-analysis. Arch Physiother. 2023;13(1):21. doi: 10.1186/s40945-023-00175-4 37845718 PMC10578022

[62] Brotis AG, Kalogeras A, Spiliotopoulos T, Fountas KN, Demetriades AK. Physical therapies after surgery for lumbar disc herniation- evidence synthesis from 55 randomized controlled trials (RCTs) and a total of 4,311 patients. Brain Spine. 2025;5:104238. doi: 10.1016/j.bas.2025.104238 40165991 PMC11957587

[63] Parrish JM, Jenkins NW, Parrish MS, Cha EDK, Lynch CP, Massel DH, et al. The influence of cognitive behavioral therapy on lumbar spine surgery outcomes: a systematic review and meta-analysis. Eur Spine J. 2021;30(5):1365–79. doi: 10.1007/s00586-021-06747-x 33566172

[64] Szeto K, Arnold J, Singh B, Gower B, Simpson CEM, Maher C. Interventions Using Wearable Activity Trackers to Improve Patient Physical Activity and Other Outcomes in Adults Who Are Hospitalized: A Systematic Review and Meta-analysis. JAMA Netw Open. 2023;6(6):e2318478. doi: 10.1001/jamanetworkopen.2023.18478 37318806 PMC10273021

[65] Gasana J, O’Keeffe T, Withers TM, Greaves CJ. A systematic review and meta-analysis of the long-term effects of physical activity interventions on objectively measured outcomes. BMC Public Health. 2023;23(1):1697. doi: 10.1186/s12889-023-16541-7 37660119 PMC10474717

[66] Pritchard MW, Lewis SR, Robinson A, Gibson SV, Chuter A, Copeland RJ, et al. Effectiveness of the perioperative encounter in promoting regular exercise and physical activity: a systematic review and meta-analysis. EClinicalMedicine. 2023;57:101806. doi: 10.1016/j.eclinm.2022.101806 36816345 PMC9929685

[67] Wagnild JM, Akowuah E, Maier RH, Hancock HC, Kasim A. Impact of prehabilitation on objectively measured physical activity levels in elective surgery patients: a systematic review. BMJ Open. 2021;11(9):e049202. doi: 10.1136/bmjopen-2021-049202 34493516 PMC8424868

[68] Clement RC, Welander A, Stowell C, Cha TD, Chen JL, Davies M, et al. A proposed set of metrics for standardized outcome reporting in the management of low back pain. Acta Orthop. 2015;86(5):523–33. doi: 10.3109/17453674.2015.1036696 25828191 PMC4564773

[69] Hopewell S, Chan A-W, Collins GS, Hróbjartsson A, Moher D, Schulz KF, et al. CONSORT 2025 statement: updated guideline for reporting randomised trials. BMJ. 2025;389:e081123. doi: 10.1136/bmj-2024-081123 40228833 PMC11995449

[70] Reyes-Ferrada W, Chirosa-Rios L, Martinez-Garcia D, Rodríguez-Perea Á, Jerez-Mayorga D. Reliability of trunk strength measurements with an isokinetic dynamometer in non-specific low back pain patients: A systematic review. J Back Musculoskelet Rehabil. 2022;35(5):937–48. doi: 10.3233/BMR-210261 35213350

[71] Mobbs RJ, Phan K, Maharaj M, Rao PJ. Physical Activity Measured with Accelerometer and Self-Rated Disability in Lumbar Spine Surgery: A Prospective Study. Global Spine J. 2016;6(5):459–64. doi: 10.1055/s-0035-1565259 27433430 PMC4947409

[72] Paulsen RT, Bergholdt E, Carreon L, Rousing R, Hansen KH, Andersen M. No differences in post-operative rehabilitation across municipalities in patients with lumbar disc herniation. Dan Med J. 2015;62(7):A5104. 26183045

