# Supplementary material for: Effects of rehabilitation and behavior change interventions on physical capacity and physical activity behavior following lumbar surgery for degenerative disease: A systematic review and meta-analysis
Source: PLoS One. 2026 Apr 20;21(4):e0347420. doi: 10.1371/journal.pone.0347420 (PMC13094952; doi:10.1371/journal.pone.0347420)
Supplement: S1 File — (DOCX) [file pone.0347420.s002.docx]

S1 File. Definitions of interventions

1. Exercise: A regimen or plan of physical activities designed and prescribed for specific therapeutic goals. Its purpose is to restore normal musculoskeletal function or to reduce pain caused by diseases or injuries. ^1^
   1. Supervised exercise: A structured exercise intervention in which participants receive synchronous supervision from a professional (e.g., an initial instructional session to ensure correct technique, or individual/group sessions conducted during the intervention period), either face-to-face or via videocall. ^2^
   2. Self-directed exercise: An exercise intervention that does not include synchronous supervision by a healthcare or exercise professional. This may involve remote monitoring methods (e.g., follow-up phone calls or questionnaires) to assess adherence or frequency, but the exercises are performed independently by the patient. ^2^
2. Physical activity advice: A flexible health promotion strategy delivered by any health professional, tailored to the patient and clinical context. It involves providing guidance on physical activity based on available time, healthcare setting, and the level of specificity required. ^3^
3. Psychologically informed rehabilitation: An approach that combines physical, behavioral, and psychological treatments to reduce pain-related disability and improve overall health. It addresses how patients respond to pain through education, behavior change, cognitive-behavioral, psychophysiological, and contextual cognitive-behavioral interventions, integrating these into rehabilitation programs. ^4^
4. Prehabilitation: A process from diagnosis to surgery, consisting of one or more preoperative interventions of exercise, nutrition, psychological strategies and respiratory training, that aims to enhance functional capacity and physiological reserve to allow patients to withstand surgical stressors, improve postoperative outcomes, and facilitate recovery. ^5^
5. Minimal/Usual care: The standard care that the targeted patient population would typically receive as part of routine clinical practice. ^6^

References

1. National Library of Medicine (US). Exercise Therapy [MeSH term]. Bethesda (MD): National Library of Medicine; [updated 2018 Jun 29; cited 2025 Jun 2]. Available from: <https://meshb.nlm.nih.gov/record/ui?ui=D005081>
2. Gómez-Redondo P, Valenzuela PL, Morales JS, Ara I, Mañas A. Supervised Versus Unsupervised Exercise for the Improvement of Physical Function and Well-Being Outcomes in Older Adults: A Systematic Review and Meta-analysis of Randomized Controlled Trials. Sports Med Auckl Nz. 2024;54(7):1877–906
3. Freene N, Barrett S, Cox ER, Hill J, Lay R, Seymour J, et al. The Physical Activity Advice Continuum-A Guide for Physical Activity Promotion in Health Care. J Phys Act Health. 2024;21(4):311–5.
4. Ballengee LA, Zullig LL, George SZ. Implementation of Psychologically Informed Physical Therapy for Low Back Pain: Where Do We Stand, Where Do We Go? J Pain Res. 2021;14:3747–57.
5. Fleurent-Grégoire C, Burgess N, McIsaac DI, Chevalier S, Fiore JF, Carli F, et al. Towards a common definition of surgical prehabilitation: a scoping review of randomised trials. Br J Anaesth. 2024;133(2):305–15
6. Yorganci E, Evans CJ, Johnson H, Barclay S, Murtagh FE, Yi D, et al. Understanding usual care in randomised controlled trials of complex interventions: A multi-method approach. Palliat Med. 2020;34(5):667–79
