# Supplementary material for: Effects of rehabilitation and behavior change interventions on physical capacity and physical activity behavior following lumbar surgery for degenerative disease: A systematic review and meta-analysis
Source: PLoS One. 2026 Apr 20;21(4):e0347420. doi: 10.1371/journal.pone.0347420 (PMC13094952; doi:10.1371/journal.pone.0347420)
Supplement: S2 File — (DOCX) [file pone.0347420.s003.docx]

S2 File. Search strategies applied to EMBASE, MEDLINE, PsycINFO and CENTRAL

Embase @ Elsevier

November 13, 2019

| No. | Query | Results |
| --- | --- | --- |
| #40 | #5 AND #38 AND #39 | **988** |
| #39 | ('randomized controlled trial' OR randomized:ab OR randomised:ab OR placebo:ab OR randomly:ab OR trial:ab OR groups:ab) NOT 'nonhuman'/exp | **3,379,343** |
| #38 | #6 OR #7 OR #8 OR #9 OR #10 OR #11 OR #12 OR #13 OR #14 OR #15 OR #16 OR #17 OR #18 OR #19 OR #20 OR #21 OR #22 OR #23 OR #24 OR #25 OR #26 OR #27 OR #28 OR #29 OR #30 OR #31 OR #32 OR #33 OR #34 OR #35 OR #36 OR #37 | **1,708,419** |
| #37 | 'magnetotherapy'/exp | **1,324** |
| #36 | 'kinesiophobia'/exp | **187** |
| #35 | 'flexibility'/exp | **165** |
| #34 | 'prehabilitation'/exp | **51** |
| #33 | 'pedometer'/exp | **2,125** |
| #32 | 'rehabilitation'/exp | **385,255** |
| #31 | 'massage'/exp | **14,394** |
| #30 | 'exercise'/exp | **335,309** |
| #29 | 'chiropractic'/exp | **4,655** |
| #28 | 'chiropractor'/exp | **609** |
| #27 | 'transcutaneous electrical nerve stimulation'/exp | **7,949** |
| #26 | 'physiotherapy'/de | **86,240** |
| #25 | 'lifestyle modification'/exp | **36,212** |
| #24 | 'behavior change'/exp | **33,342** |
| #23 | 'pedomet*':ti,ab | **3,537** |
| #22 | 'aerobic activit*':ti,ab | **934** |
| #21 | 'lifestyle modif*':ti,ab | **9,471** |
| #20 | 'lifestyle change*':ti,ab | **11,149** |
| #19 | 'massage therap*':ti,ab | **1,680** |
| #18 | hydrotherap*:ti,ab | **1,493** |
| #17 | exerci*:ti,ab | **380,271** |
| #16 | 'chiropract*':ti,ab | **5,717** |
| #15 | 'transcutaneous electrical nerve stimulat*':ti,ab | **2,509** |
| #14 | tens:ti,ab | **15,266** |
| #13 | 'physiotherap*':ti,ab | **43,790** |
| #12 | 'physical therap*':ti,ab | **32,222** |
| #11 | 'magnetic field therapy':ti,ab | **119** |
| #10 | kinesiophobi*:ti,ab | **1,048** |
| #9 | flexib*:ti,ab | **187,534** |
| #8 | prehabilitat*:ti,ab | **701** |
| #7 | walk*:ti,ab | **155,882** |
| #6 | ((foster* OR promot* OR enhanc* OR engag* OR intervention* OR counselling OR motivat* OR reinforc* OR support* OR 'cognitive behavio*' OR therap* OR program* OR psych*) NEAR/3 (behavio* OR health* OR physical OR lifestyle OR change OR recover*)):ti,ab | **532,613** |
| #5 | #4 NOT ('lung surger*' OR 'lung transplant*' OR 'lung cancer') | **44,146** |
| #4 | #1 OR #2 OR #3 | **49,413** |
| #3 | 'lumbar surgery'/exp | **13** |
| #2 | ((posterior NEAR/2 (surger* OR fusion* OR pedicle* OR discectom* OR procedure* OR revision* OR 'post operative' OR 'postoperative' OR correction*)):ab,ti) AND (spine:ab,ti OR spinal:ab,ti) | **6,596** |
| #1 | ((thoracolumbar OR lumbar OR thoracic) NEAR/2 (surger* OR fusion* OR pedicle* OR discectom* OR procedure* OR revision* OR 'post operative' OR 'postoperative' OR correction*)):ab,ti | **44,285** |

Medline @ Ovid

November 13, 2019

| No. | Search String | Results |
| --- | --- | --- |
| 1 | ((thoracolumbar or lumbar or thoracic) adj2 (surger* or fusion* or pedicle* or discectom* or procedure* or revision* or "post operative" or postoperative or correction*)).ab,ti. | 26110 |
| 2 | (posterior adj2 (surger* or fusion* or pedicle* or discectom* or procedure* or revision* or "post operative" or postoperative or correction*)).mp. and (spine or spinal).ab,ti. [mp=title, abstract, original title, name of substance word, subject heading word, floating sub-heading word, keyword heading word, organism supplementary concept word, protocol supplementary concept word, rare disease supplementary concept word, unique identifier, synonyms] | 5247 |
| 3 | 1 or 2 | 30245 |
| 4 | (((thoracolumbar or lumbar or thoracic) adj2 (surger* or fusion* or pedicle* or discectom* or procedure* or revision* or "post operative" or postoperative or correction*)).ab,ti. or ((posterior adj2 (surger* or fusion* or pedicle* or discectom* or procedure* or revision* or "post operative" or postoperative or correction*)).mp. and (spine or spinal).ab,ti.)) not ("lung surgery" or "lung transplant*" or "lung cancer").mp. [mp=title, abstract, original title, name of substance word, subject heading word, floating sub-heading word, keyword heading word, organism supplementary concept word, protocol supplementary concept word, rare disease supplementary concept word, unique identifier, synonyms] | 28214 |
| 5 | ((foster* or promot* or enhanc* or engag* or intervention* or counselling or motivat* or reinforc* or support* or "cognitive behavio*" or therap* or program* or psych*) adj3 (behavio* or health* or physical or lifestyle or change or recover*)).ab,ti. | 406430 |
| 6 | "walk*".ab,ti. | 111285 |
| 7 | "prehabilitat*".ab,ti. | 442 |
| 8 | "flexib*".ab,ti. | 164751 |
| 9 | "kinesiophob*".ab,ti. | 730 |
| 10 | "magnetic field therapy".ab,ti. | 84 |
| 11 | "physical therap* ".ab,ti. | 21186 |
| 12 | "physiotherap*".ab,ti. | 24162 |
| 13 | tens.ab,ti. | 14917 |
| 14 | "transcutaneous electrical nerve stimulation".ab,ti. | 1847 |
| 15 | "chiropract*".ab,ti. | 5624 |
| 16 | "exerci*".ab,ti. | 281274 |
| 17 | "hydrotherap*".ab,ti. | 964 |
| 18 | "massage therap* ".ab,ti. | 1250 |
| 19 | "lifestyle modif* ".ab,ti. | 6270 |
| 20 | "lifestyle change* ".ab,ti. | 7528 |
| 21 | "aerobic activit* ".ab,ti. | 665 |
| 22 | "pedomet*".ab,ti. | 2581 |
| 23 | exp Physical Therapy Modalities/ | 147306 |
| 24 | exp Exercise/ | 185225 |
| 25 | rehabilitation/ or dance therapy/ or early ambulation/ or exercise therapy/ or neurological rehabilitation/ or occupational therapy/ or recreation therapy/ or rehabilitation, vocational/ or telerehabilitation/ | 80282 |
| 26 | 5 or 6 or 7 or 8 or 9 or 10 or 11 or 12 or 13 or 14 or 15 or 16 or 17 or 18 or 19 or 20 or 21 or 22 or 23 or 24 or 25 | 1135067 |
| 27 | ((randomized controlled trial or controlled clinical trial).pt. or randomized.ab. or randomised.ab. or placebo.ab. or drug therapy.fs. or randomly.ab. or trial.ab. or groups.ab.) not (exp animals/ not humans.sh.) | 3970745 |
| 28 | 4 and 26 and 27 | 581 |

PsycINFO @ EBSCO

November 13, 2019

| **#** | **Query** | **Results** |
| --- | --- | --- |
| S47 | S6 AND S45 AND S46 | 34 |
| S46 | ( PT("randomized controlled trial") OR AB(randomized OR randomised OR placebo OR randomly OR trial OR groups) ) NOT (MH "Animals+") | 1,004,246 |
| S45 | S7 OR S8 OR S9 OR S10 OR S11 OR S12 OR S13 OR S14 OR S15 OR S16 OR S17 OR S18 OR S19 OR S20 OR S21 OR S22 OR S23 OR S24 OR S25 OR S26 OR S27 OR S28 OR S29 OR S30 OR S31 OR S32 OR S33 OR S34 OR S35 OR S36 OR S37 OR S38 OR S39 OR S40 OR S41 OR S42 OR S43 OR S44 | 738,993 |
| S44 | DE "Rehabilitation" OR DE "Cognitive Rehabilitation" OR DE "Criminal Rehabilitation" OR DE "Neuropsychological Rehabilitation" OR DE "Neurorehabilitation" OR DE "Occupational Therapy" OR DE "Physical Therapy" OR DE "Psychosocial Rehabilitation" OR DE "Rehabilitation Centers" OR DE "Telerehabilitation" | 40,527 |
| S43 | DE "Behavior Modification" OR DE "Behavior Contracting" OR DE "Behavior Therapy" OR DE "Biofeedback Training" OR DE "Classroom Behavior Modification" OR DE "Contingency Management" OR DE "Fading (Conditioning)" OR DE "Omission Training" OR DE "Overcorrection" OR DE "Self-Management" OR DE "Time Out" OR DE "Behavior Change" OR DE "Readiness to Change" OR DE "Stages of Change" OR DE "Cognitive Behavior Therapy" OR DE "Acceptance and Commitment Therapy" OR DE "Cognitive Processing Therapy" OR DE "Prolonged Exposure Therapy" OR DE "Cognitive Therapy" | 92,946 |
| S42 | AB pedomet* | 824 |
| S41 | TI pedomet* | 162 |
| S40 | AB "aerobic activit*" | 154 |
| S39 | TI "aerobic activit*" | 22 |
| S38 | AB "lifestyle change*" | 1,947 |
| S37 | TI "lifestyle change*" | 246 |
| S36 | AB "lifestyle modif*" | 648 |
| S35 | TI "lifestyle modif*" | 104 |
| S34 | AB "massage therap*" | 367 |
| S33 | TI "massage therap*" | 137 |
| S32 | AB hydrotherap* | 119 |
| S31 | TI hydrotherap* | 25 |
| S30 | AB exerci* | 62,563 |
| S29 | TI exerci* | 15,428 |
| S28 | AB chiropract* | 401 |
| S27 | TI chiropract* | 103 |
| S26 | AB "transcutaneous electrical nerve stimulation" | 269 |
| S25 | TI "transcutaneous electrical nerve stimulation" | 142 |
| S24 | AB tens | 214,619 |
| S23 | TI tens | 5,544 |
| S22 | AB physiotherap* | 2,907 |
| S21 | TI physiotherap* | 529 |
| S20 | AB "physical therap*" | 3,248 |
| S19 | TI "physical therap*" | 781 |
| S18 | AB "magnetic field therapy" | 3 |
| S17 | TI "magnetic field therapy" | 2 |
| S16 | AB kinesiophob* | 189 |
| S15 | TI kinesiophob* | 37 |
| S14 | AB flexib* | 46,456 |
| S13 | TI flexib* | 5,290 |
| S12 | AB prehabilitat* | 16 |
| S11 | TI prehabilitat* | 8 |
| S10 | AB walk* | 24,373 |
| S9 | TI walk* | 4,831 |
| S8 | AB ((foster* or promot* or enhanc* or engag* or intervention* or counselling or motivat* or reinforc* or support* or "cognitive behavio*" or therap* or program* or psych*) N3 (behavio* or health* or physical or lifestyle or change or recover*)) | 339,758 |
| S7 | TI ((foster* or promot* or enhanc* or engag* or intervention* or counselling or motivat* or reinforc* or support* or "cognitive behavio*" or therap* or program* or psych*) N3 (behavio* or health* or physical or lifestyle or change or recover*)) | 74,393 |
| S6 | S5 NOT ("lung surgery" OR "lung transplant*" OR "lung cancer") | 325 |
| S5 | S1 OR S2 OR S3 OR S4 | 338 |
| S4 | AB ((posterior N2 (surger* or fusion* or pedicle* or discectom* or procedure* or revision* or "post operative" or postoperative or correction*))) AND (spine OR spinal) | 13 |
| S3 | TI ((posterior N2 (surger* or fusion* or pedicle* or discectom* or procedure* or revision* or "post operative" or postoperative or correction*))) AND (spine OR spinal) | 0 |
| S2 | AB ((thoracolumbar or lumbar or thoracic) N2 (surger* or fusion* or pedicle* or discectom* or procedure* or revision* or "post operative" or postoperative or correction*)) | 299 |
| S1 | TI ((thoracolumbar or lumbar or thoracic) N2 (surger* or fusion* or pedicle* or discectom* or procedure* or revision* or "post operative" or postoperative or correction*)) | 115 |

CENTRAL @ Cochrane Library

November 13, 2019

| ID | Search | Hits |
| --- | --- | --- |
| #1 | ((thoracolumbar or lumbar or thoracic) NEAR/2 (surger* or fusion* or pedicle* or discectom* or procedure* or revision* or "post operative" or postoperative or correction*)) | 8234 |
| #2 | (posterior NEAR/2 (surger* or fusion* or pedicle* or discectom* or procedure* or revision* or "post operative" or postoperative or correction*)) and (spine or spinal) | 656 |
| #3 | #1 OR #2 | 8615 |
| #4 | #3 NOT ("lung surgery" OR "lung transplant*" OR "lung cancer") | 7822 |
| #5 | ((foster* or promot* or enhanc* or engag* or intervention* or counselling or motivat* or reinforc* or support* or "cognitive behavio*" or therap* or program* or psych*) NEAR/3 (behavio* or health* or physical or lifestyle or change or recover*)) | 125638 |
| #6 | walk* | 33257 |
| #7 | prehabilitat* | 255 |
| #8 | flexib* | 11741 |
| #9 | kinesiophob* | 439 |
| #10 | magnetic field therap* | 0 |
| #11 | physical therap* | 64 |
| #12 | physiotherap* | 17512 |
| #13 | tens | 2021 |
| #14 | transcutaneous electrical nerve stimulation | 1801 |
| #15 | chiropract* | 1273 |
| #16 | exerci* | 94570 |
| #17 | hydrotherap* | 542 |
| #18 | massage therap* | 0 |
| #19 | lifestyle modif* | 3 |
| #20 | lifestyle change* | 680 |
| #21 | aerobic activit* | 1 |
| #22 | pedomet* | 1580 |
| #23 | MeSH descriptor: [Physical Therapy Modalities] explode all trees | 23520 |
| #24 | MeSH descriptor: [Exercise] explode all trees | 22647 |
| #25 | MeSH descriptor: [Rehabilitation] explode all trees | 33047 |
| #26 | #5 OR #6 OR #7 OR #8 OR #9 OR #10 OR #11 OR #12 OR #13 OR #14 OR #15 OR #16 OR #17 OR #18 OR #19 OR #20 OR #21 OR #22 OR #23 OR #24 OR #25 | 238535 |
| #27 | #4 AND #26 in Trials | 747 |

Embase @ Elsevier

July 3, 2024

| Search String | Results |
| --- | --- |
| ((((thoracolumbar OR lumbar OR thoracic) NEAR/2 (surger* OR fusion* OR pedicle* OR discectom* OR diskectom* OR procedure* OR revision* OR 'post operative' OR 'postoperative' OR correction* OR endoscop* OR microsurg* OR microendoscop* OR microdiscectom* OR microdiskectom* OR fixation* OR replacement* OR arthroplast* OR arthrodesis OR 'minimally invasive')):ab,ti) OR (((posterior NEAR/2 (surger* OR fusion* OR pedicle* OR discectom* OR diskectom* OR procedure* OR revision* OR 'post operative' OR 'postoperative' OR correction* OR endoscop* OR microsurg* OR microendoscop* OR microdiscectom* OR microdiskectom* OR fixation* OR replacement* OR arthroplast* OR arthrodesis OR 'minimally invasive')):ab,ti) AND (spine:ab,ti OR spinal:ab,ti)) OR (((((spin* OR lumb* OR thoracic) NEAR/3 (stenosis OR spondyl*)):ti,ab) OR lss:ti,ab OR (((disc OR disk OR vertebra* OR intervertebra*) NEAR/2 (herniat* OR protrusion* OR lesion* OR degenerat* OR displace*)):ti,ab) OR 'spondylosis'/exp OR 'spondylolisthesis'/exp OR 'intervertebral disk disease'/exp OR 'vertebral canal stenosis'/exp) AND (surg*:ti,ab OR fusion*:ti,ab OR pedicle*:ti,ab OR discectom*:ti,ab OR diskectom*:ti,ab OR revision*:ti,ab OR 'post operative':ti,ab OR postoperative:ti,ab OR preoperative:ti,ab OR correction*:ti,ab OR decompression*:ti,ab OR presurg*:ti,ab OR microendoscop*:ti,ab OR microsurg*:ti,ab OR microdiscectom*:ti,ab OR microdiskectom*:ti,ab OR replacement*:ti,ab OR arthroplast*:ti,ab OR arthrodesis:ti,ab OR 'minimally invasive':ti,ab OR 'microsurgery'/exp OR 'endoscopic surgery'/exp OR 'spine surgery'/exp))) AND ((((foster* OR promot* OR enhanc* OR engag* OR intervention* OR counselling OR motivat* OR reinforc* OR support* OR 'cognitive behavio*' OR therap* OR program* OR psych*) NEAR/3 (behavio* OR health* OR physical OR lifestyle OR change OR recover*)):ti,ab) OR walk*:ti,ab OR rehabilitat*:ti,ab OR prehabilitat*:ti,ab OR flexib*:ti,ab OR kinesiophob*:ti,ab OR 'physical therap*':ti,ab OR 'physiotherap*':ti,ab OR 'magnetic field therapy':ti,ab OR 'magnetotherapy':ti,ab OR 'transcutaneous electrical nerve stimulation':ti,ab OR tens:ti,ab OR chiropract*:ti,ab OR exerci*:ti,ab OR hydrotherap*:ti,ab OR 'massage therap*':ti,ab OR 'lifestyle change*':ti,ab OR 'lifestyle modif*':ti,ab OR 'aerobic activit*':ti,ab OR pedomet*:ti,ab OR 'behaviour change'/exp OR 'lifestyle modification'/exp OR 'physiotherapy'/de OR 'transcutaneous electrical nerve stimulation'/exp OR 'chiropractor'/exp OR 'chiropractic'/exp OR 'exercise'/exp OR 'massage'/exp OR 'rehabilitation'/exp OR 'pedometer'/exp OR 'prehabilitation'/exp OR 'flexibility'/exp OR 'kinesiophobia'/exp OR 'magnetotherapy'/exp) AND ('randomized controlled trial' OR randomized:ab OR randomised:ab OR placebo:ab OR randomly:ab OR trial:ab OR groups:ab) NOT 'nonhuman'/exp AND [21-11-2019]/sd NOT [04-07-2024]/sd 1140 | 1140 |

Medline @ Ovid

July 3, 2024

| No. | Search String | Results |
| --- | --- | --- |
| 1 | ((thoracolumbar or lumbar or thoracic) adj2 (surger* or fusion* or pedicle* or discectom* or procedure* or revision* or "post operative" or postoperative or correction*)).ab,ti. | 26110 |
| 2 | (posterior adj2 (surger* or fusion* or pedicle* or discectom* or procedure* or revision* or "post operative" or postoperative or correction*)).mp. and (spine or spinal).ab,ti. [mp=title, abstract, original title, name of substance word, subject heading word, floating sub-heading word, keyword heading word, organism supplementary concept word, protocol supplementary concept word, rare disease supplementary concept word, unique identifier, synonyms] | 5247 |
| 3 | 1 or 2 | 30245 |
| 4 | (((thoracolumbar or lumbar or thoracic) adj2 (surger* or fusion* or pedicle* or discectom* or procedure* or revision* or "post operative" or postoperative or correction*)).ab,ti. or ((posterior adj2 (surger* or fusion* or pedicle* or discectom* or procedure* or revision* or "post operative" or postoperative or correction*)).mp. and (spine or spinal).ab,ti.)) not ("lung surgery" or "lung transplant*" or "lung cancer").mp. [mp=title, abstract, original title, name of substance word, subject heading word, floating sub-heading word, keyword heading word, organism supplementary concept word, protocol supplementary concept word, rare disease supplementary concept word, unique identifier, synonyms] | 28214 |
| 5 | ((foster* or promot* or enhanc* or engag* or intervention* or counselling or motivat* or reinforc* or support* or "cognitive behavio*" or therap* or program* or psych*) adj3 (behavio* or health* or physical or lifestyle or change or recover*)).ab,ti. | 406430 |
| 6 | "walk*".ab,ti. | 111285 |
| 7 | "prehabilitat*".ab,ti. | 442 |
| 8 | "flexib*".ab,ti. | 164751 |
| 9 | "kinesiophob*".ab,ti. | 730 |
| 10 | "magnetic field therapy".ab,ti. | 84 |
| 11 | "physical therap* ".ab,ti. | 21186 |
| 12 | "physiotherap*".ab,ti. | 24162 |
| 13 | tens.ab,ti. | 14917 |
| 14 | "transcutaneous electrical nerve stimulation".ab,ti. | 1847 |
| 15 | "chiropract*".ab,ti. | 5624 |
| 16 | "exerci*".ab,ti. | 281274 |
| 17 | "hydrotherap*".ab,ti. | 964 |
| 18 | "massage therap* ".ab,ti. | 1250 |
| 19 | "lifestyle modif* ".ab,ti. | 6270 |
| 20 | "lifestyle change* ".ab,ti. | 7528 |
| 21 | "aerobic activit* ".ab,ti. | 665 |
| 22 | "pedomet*".ab,ti. | 2581 |
| 23 | exp Physical Therapy Modalities/ | 147306 |
| 24 | exp Exercise/ | 185225 |
| 25 | rehabilitation/ or dance therapy/ or early ambulation/ or exercise therapy/ or neurological rehabilitation/ or occupational therapy/ or recreation therapy/ or rehabilitation, vocational/ or telerehabilitation/ | 80282 |
| 26 | 5 or 6 or 7 or 8 or 9 or 10 or 11 or 12 or 13 or 14 or 15 or 16 or 17 or 18 or 19 or 20 or 21 or 22 or 23 or 24 or 25 | 1135067 |
| 27 | ((randomized controlled trial or controlled clinical trial).pt. or randomized.ab. or randomised.ab. or placebo.ab. or drug therapy.fs. or randomly.ab. or trial.ab. or groups.ab.) not (exp animals/ not humans.sh.) | 3970745 |
| 28 | 4 and 26 and 27 | 581 |

PsycINFO @ EBSCO

July 3, 2024

| # | Query | Results |
| --- | --- | --- |
| S101 | ( S55 AND S98 AND S99 ) AND ( ZD (201911* OR 2020* OR 2021* OR 2022* OR 2023* OR 2024*) ) | 25 |
| S100 | S55 AND S98 AND S99 | 80 |
| S99 | ( PT("randomized controlled trial") OR AB(randomized OR randomised OR placebo OR randomly OR trial OR groups) ) NOT (MH "Animals+") | 1,232,134 |
| S98 | S56 OR S57 OR S58 OR S59 OR S60 OR S61 OR S62 OR S63 OR S64 OR S65 OR S66 OR S67 OR S68 OR S69 OR S70 OR S71 OR S72 OR S73 OR S74 OR S75 OR S76 OR S77 OR S78 OR S79 OR S80 OR S81 OR S82 OR S83 OR S84 OR S85 OR S86 OR S87 OR S88 OR S89 OR S90 OR S91 OR S92 OR S93 OR S94 OR S95 OR S96 OR S97 | 965,972 |
| S97 | DE "Rehabilitation" OR DE "Cognitive Rehabilitation" OR DE "Criminal Rehabilitation" OR DE "Neuropsychological Rehabilitation" OR DE "Neurorehabilitation" OR DE "Occupational Therapy" OR DE "Physical Therapy" OR DE "Psychosocial Rehabilitation" OR DE "Rehabilitation Centers" OR DE "Telerehabilitation" | 50,392 |
| S96 | DE "Behavior Modification" OR DE "Behavior Contracting" OR DE "Behavior Therapy" OR DE "Biofeedback Training" OR DE "Classroom Behavior Modification" OR DE "Contingency Management" OR DE "Fading (Conditioning)" OR DE "Omission Training" OR DE "Overcorrection" OR DE "Self-Management" OR DE "Time Out" OR DE "Behavior Change" | 65,834 |
| S95 | AB pedomet* | 977 |
| S94 | TI pedomet* | 182 |
| S93 | AB "aerobic activit*" | 202 |
| S92 | TI "aerobic activit*" | 26 |
| S91 | AB "lifestyle modif*" | 917 |
| S90 | TI "lifestyle modif*" | 151 |
| S89 | AB "lifestyle change*" | 2,619 |
| S88 | TI "lifestyle change*" | 304 |
| S87 | AB "massage therap*" | 432 |
| S86 | TI "massage therap*" | 155 |
| S85 | AB hydrotherap* | 136 |
| S84 | TI hydrotherap* | 29 |
| S83 | AB exerci* | 78,961 |
| S82 | TI exerci* | 20,131 |
| S81 | AB chiropract* | 480 |
| S80 | TI chiropract* | 127 |
| S79 | AB tens | 260,909 |
| S78 | TI tens | 6,449 |
| S77 | AB "transcutaneous electrical nerve stimulation" | 344 |
| S76 | TI "transcutaneous electrical nerve stimulation" | 171 |
| S75 | AB "magnetotherapy" | 14 |
| S74 | TI "magnetotherapy" | 7 |
| S73 | AB "magnetic field therapy" | 4 |
| S72 | TI "magnetic field therapy" | 2 |
| S71 | AB "physiotherap*" | 4,138 |
| S70 | TI "physiotherap*" | 781 |
| S69 | AB "physical therap*" | 4,341 |
| S68 | TI "physical therap*" | 1,111 |
| S67 | AB kinesiophob* | 362 |
| S66 | TI kinesiophob* | 80 |
| S65 | AB flexib* | 62,771 |
| S64 | TI flexib* | 7,526 |
| S63 | AB prehabilitat* | 55 |
| S62 | TI prehabilitat* | 31 |
| S61 | AB rehabilitat* | 63,798 |
| S60 | TI rehabilitat* | 20,802 |
| S59 | AB walk* | 31,196 |
| S58 | TI walk* | 6,342 |
| S57 | AB ((foster* OR promot* OR enhanc* OR engag* OR intervention* OR counselling OR motivat* OR reinforc* OR support* OR 'cognitive behavio*' OR therap* OR program* OR psych*) N3 (behavio* OR health* OR physical OR lifestyle OR change OR recover*)) | 467,486 |
| S56 | TI ((foster* OR promot* OR enhanc* OR engag* OR intervention* OR counselling OR motivat* OR reinforc* OR support* OR 'cognitive behavio*' OR therap* OR program* OR psych*) N3 (behavio* OR health* OR physical OR lifestyle OR change OR recover*)) | 97,718 |
| S55 | S1 OR S2 OR S3 OR S4 OR S54 | 684 |
| S54 | S11 AND S53 | 288 |
| S53 | S12 OR S13 OR S14 OR S15 OR S16 OR S17 OR S18 OR S19 OR S20 OR S21 OR S22 OR S23 OR S24 OR S25 OR S26 OR S27 OR S28 OR S29 OR S30 OR S31 OR S32 OR S33 OR S34 OR S35 OR S36 OR S37 OR S38 OR S39 OR S40 OR S41 OR S42 OR S43 OR S44 OR S45 OR S46 OR S47 OR S48 OR S49 OR S50 OR S51 OR S52 | 187,857 |
| S52 | DE "Surgery" OR DE "Postsurgical Complications" OR DE "Sterilization (Sex)" OR DE "Transection" OR DE "Amputation" OR DE "Bariatric Surgery" OR DE "Circumcision" OR DE "Cochlear Implants" OR DE "Colostomy" OR DE "Dental Surgery" OR DE "Endocrine Gland Surgery" OR DE "Gender Reassignment" OR DE "Heart Surgery" OR DE "Hysterectomy" OR DE "Induced Abortion" OR DE "Neurosurgery" OR DE "Organ Transplantation" OR DE "Plastic Surgery" OR DE "Stereotaxic Techniques" | 39,828 |
| S51 | AB "minimally invasive" | 766 |
| S50 | TI "minimally invasive" | 109 |
| S49 | AB arthrodesis | 17 |
| S48 | TI arthrodesis | 4 |
| S47 | AB arthroplast* | 612 |
| S46 | TI arthroplast* | 415 |
| S45 | AB replacement* | 13,884 |
| S44 | TI replacement* | 2,354 |
| S43 | AB microdiskectom* | 1 |
| S42 | TI microdiskectom* | 1 |
| S41 | AB microdiscectom* | 15 |
| S40 | TI microdiscectom* | 8 |
| S39 | AB microsurg* | 232 |
| S38 | TI microsurg* | 39 |
| S37 | AB microendoscop* | 25 |
| S36 | TI microendoscop* | 3 |
| S35 | AB presurg* | 1,697 |
| S34 | TI presurg* | 247 |
| S33 | AB decompression* | 728 |
| S32 | TI decompression* | 176 |
| S31 | AB correction* | 52,582 |
| S30 | TI correction* | 14,013 |
| S29 | AB preoperative | 4,427 |
| S28 | TI preoperative | 842 |
| S27 | AB postoperative | 8,629 |
| S26 | TI postoperative | 2,076 |
| S25 | AB "post operative" | 1,854 |
| S24 | TI "post operative" | 326 |
| S23 | AB revision* | 22,104 |
| S22 | TI revision* | 3,663 |
| S21 | AB diskectom* | 5 |
| S20 | TI diskectom* | 2 |
| S19 | AB discectom* | 72 |
| S18 | TI discectom* | 28 |
| S17 | AB pedicle* | 107 |
| S16 | TI pedicle* | 20 |
| S15 | AB fusion* | 9,459 |
| S14 | TI fusion* | 2,409 |
| S13 | AB surg* | 56,443 |
| S12 | TI surg* | 12,375 |
| S11 | S5 OR S6 OR S7 OR S8 OR S9 OR S10 | 997 |
| S10 | AB ((disc OR disk OR vertebra* OR intervertebra*) N2 (herniat* OR protrusion* OR lesion* OR degenerat* OR displace*)) | 406 |
| S9 | TI ((disc OR disk OR vertebra* OR intervertebra*) N2 (herniat* OR protrusion* OR lesion* OR degenerat* OR displace*)) | 115 |
| S8 | AB lss | 401 |
| S7 | TI lss | 17 |
| S6 | AB ((spin* OR lumb* OR thoracic) N3 (stenosis OR spondyl*)) | 234 |
| S5 | TI ((spin* OR lumb* OR thoracic) N3 (stenosis OR spondyl*)) | 74 |
| S4 | AB ((posterior N2 (surger* OR fusion* OR pedicle* OR discectom* OR diskectom* OR procedure* OR revision* OR 'post operative' OR 'postoperative' OR correction* OR endoscop* OR microsurg* OR microendoscop* OR microdiscectom* OR microdiskectom* OR fixation* OR replacement* OR arthroplast* OR arthrodesis OR 'minimally invasive'))) AND (spine OR spinal) | 40 |
| S3 | TI ((posterior N2 (surger* OR fusion* OR pedicle* OR discectom* OR diskectom* OR procedure* OR revision* OR 'post operative' OR 'postoperative' OR correction* OR endoscop* OR microsurg* OR microendoscop* OR microdiscectom* OR microdiskectom* OR fixation* OR replacement* OR arthroplast* OR arthrodesis OR 'minimally invasive'))) AND (spine OR spinal) | 6 |
| S2 | AB ((thoracolumbar OR lumbar OR thoracic) N2 (surger* OR fusion* OR pedicle* OR discectom* OR diskectom* OR procedure* OR revision* OR 'post operative' OR 'postoperative' OR correction* OR endoscop* OR microsurg* OR microendoscop* OR microdiscectom* OR microdiskectom* OR fixation* OR replacement* OR arthroplast* OR arthrodesis OR 'minimally invasive')) | 410 |
| S1 | TI ((thoracolumbar OR lumbar OR thoracic) N2 (surger* OR fusion* OR pedicle* OR discectom* OR diskectom* OR procedure* OR revision* OR 'post operative' OR 'postoperative' OR correction* OR endoscop* OR microsurg* OR microendoscop* OR microdiscectom* OR microdiskectom* OR fixation* OR replacement* OR arthroplast* OR arthrodesis OR 'minimally invasive')) | 170 |

CENTRAL @ Cochrane Library

July 3, 2024

| ID | Search | Hits |
| --- | --- | --- |
| #1 | ((thoracolumbar or lumbar or thoracic) NEAR/2 (surger* or fusion* or pedicle* or discectom* or diskectom* or procedure* or revision* or 'post operative' or 'postoperative' or correction* or endoscop* or microsurg* or microendoscop* or microdiscectom* or microdiskectom* or fixation* or replacement* or arthroplast* or arthrodesis or 'minimally invasive')):ab,ti | 7114 |
| #2 | ((posterior NEAR/2 (surger* or fusion* or pedicle* or discectom* or diskectom* or procedure* or revision* or 'post operative' or 'postoperative' or correction* or endoscop* or microsurg* or microendoscop* or microdiscectom* or microdiskectom* or fixation* or replacement* or arthroplast* or arthrodesis or 'minimally invasive')) and (spine or spinal)):ab,ti | 788 |
| #3 | ((spin* or lumb* or thoracic) NEAR/3 (stenosis or spondyl*)):ab,ti | 2056 |
| #4 | lss:ab,ti | 417 |
| #5 | ((disc or disk or vertebra* or intervertebra*) NEAR/2 (herniat* or protrusion* or lesion* or degenerat* or displace*)):ab,ti | 3748 |
| #6 | MeSH descriptor: [Spinal Diseases] this term only | 623 |
| #7 | MeSH descriptor: [Intervertebral Disc Degeneration] explode all trees | 625 |
| #8 | MeSH descriptor: [Intervertebral Disc Displacement] explode all trees | 1396 |
| #9 | MeSH descriptor: [Spinal Stenosis] explode all trees | 687 |
| #10 | MeSH descriptor: [Spondylitis] explode all trees | 2036 |
| #11 | MeSH descriptor: [undefined] explode all trees | 0 |
| #12 | #3 OR #4 OR #5 OR #6 OR #7 OR #8 OR #9 OR #10 OR #11 | 8824 |
| #13 | surg*:ab,ti | 276049 |
| #14 | fusion*:ab,ti | 8117 |
| #15 | pedicle*:ab,ti | 1579 |
| #16 | discectom*:ab,ti | 1626 |
| #17 | diskectom*:ab,ti | 72 |
| #18 | revision*:ab,ti | 5413 |
| #19 | post operative:ab,ti | 31889 |
| #20 | preoperative:ab,ti | 43330 |
| #21 | postoperative:ab,ti | 136323 |
| #22 | correction*:ab,ti | 18859 |
| #23 | decompression*:ab,ti | 3491 |
| #24 | microendoscop*:ab,ti | 80 |
| #25 | microsurg*:ab,ti | 1129 |
| #26 | microdiscectom*:ab,ti | 300 |
| #27 | microdiskectom*:ab,ti | 21 |
| #28 | replacement*:ab,ti | 31420 |
| #29 | arthroplast*:ab,ti | 13831 |
| #30 | arthrodesis:ab,ti | 518 |
| #31 | minimally invasive:ab,ti | 8315 |
| #32 | MeSH descriptor: [Surgical Procedures, Operative] explode all trees | 174359 |
| #33 | #13 OR #14 OR #15 OR #16 OR #17 OR #18 OR #19 OR #20 OR #21 OR #22 OR #23 OR #24 OR #25 OR #26 OR #27 OR #28 OR #29 OR #30 OR #31 OR #32 | 447457 |
| #34 | #12 AND #33 | 4461 |
| #35 | #1 OR #2 OR #34 | 10451 |
| #36 | ((foster* or promot* or enhanc* or engag* or intervention* or counselling or motivat* or reinforc* or support* or 'cognitive behavio*' or therap* or program* or psych*) NEAR/3 (behavio* or health* or physical or lifestyle or change or recover*)):ab,ti | 215550 |
| #37 | walk*:ab,ti | 42492 |
| #38 | rehabilitat*:ab,ti | 50218 |
| #39 | prehabilitat*:ab,ti | 782 |
| #40 | flexib*:ab,ti | 17876 |
| #41 | kinesiophob*:ab,ti | 1193 |
| #42 | physical therap*:ab,ti | 48669 |
| #43 | physiotherap*:ab,ti | 18673 |
| #44 | magnetic field therapy:ab,ti | 953 |
| #45 | magnetotherapy:ab,ti | 102 |
| #46 | transcutaneous electrical nerve stimulation:ab,ti | 3300 |
| #47 | tens:ab,ti | 3045 |
| #48 | chiropract*:ab,ti | 1192 |
| #49 | exerci*:ab,ti | 128740 |
| #50 | hydrotherap*:ab,ti | 452 |
| #51 | massage therap*:ab,ti | 3557 |
| #52 | lifestyle change*:ab,ti | 14230 |
| #53 | lifestyle modif*:ab,ti | 6214 |
| #54 | aerobic activit*:ab,ti | 7321 |
| #55 | pedomet*:ab,ti | 2131 |
| #56 | MeSH descriptor: [Physical Therapy Modalities] explode all trees | 40000 |
| #57 | MeSH descriptor: [Exercise] explode all trees | 39051 |
| #58 | MeSH descriptor: [Rehabilitation] explode all trees | 55035 |
| #59 | #36 OR #37 OR #38 OR #39 OR #40 OR #41 OR #42 OR #43 OR #44 OR #45 OR #46 OR #47 OR #48 OR #49 OR #50 OR #51 OR #52 OR #53 OR #54 OR #55 OR #56 OR #57 OR #58 | 421541 |
| #60 | #35 AND #59 with Cochrane Library publication date Between Nov 2019 and Jul 2024, in Trials | 844 |

Embase @ Elsevier

August 29, 2025

| Search String | Results |
| --- | --- |
| ((((thoracolumbar OR lumbar OR thoracic) NEAR/2 (surger* OR fusion* OR pedicle* OR discectom* OR diskectom* OR procedure* OR revision* OR 'post operative' OR 'postoperative' OR correction* OR endoscop* OR microsurg* OR microendoscop* OR microdiscectom* OR microdiskectom* OR fixation* OR replacement* OR arthroplast* OR arthrodesis OR 'minimally invasive')):ab,ti) OR (((posterior NEAR/2 (surger* OR fusion* OR pedicle* OR discectom* OR diskectom* OR procedure* OR revision* OR 'post operative' OR 'postoperative' OR correction* OR endoscop* OR microsurg* OR microendoscop* OR microdiscectom* OR microdiskectom* OR fixation* OR replacement* OR arthroplast* OR arthrodesis OR 'minimally invasive')):ab,ti) AND (spine:ab,ti OR spinal:ab,ti)) OR (((((spin* OR lumb* OR thoracic) NEAR/3 (stenosis OR spondyl*)):ti,ab) OR lss:ti,ab OR (((disc OR disk OR vertebra* OR intervertebra*) NEAR/2 (herniat* OR protrusion* OR lesion* OR degenerat* OR displace*)):ti,ab) OR 'spondylosis'/exp OR 'spondylolisthesis'/exp OR 'intervertebral disk disease'/exp OR 'vertebral canal stenosis'/exp) AND (surg*:ti,ab OR fusion*:ti,ab OR pedicle*:ti,ab OR discectom*:ti,ab OR diskectom*:ti,ab OR revision*:ti,ab OR 'post operative':ti,ab OR postoperative:ti,ab OR preoperative:ti,ab OR correction*:ti,ab OR decompression*:ti,ab OR presurg*:ti,ab OR microendoscop*:ti,ab OR microsurg*:ti,ab OR microdiscectom*:ti,ab OR microdiskectom*:ti,ab OR replacement*:ti,ab OR arthroplast*:ti,ab OR arthrodesis:ti,ab OR 'minimally invasive':ti,ab OR 'microsurgery'/exp OR 'endoscopic surgery'/exp OR 'spine surgery'/exp))) AND ((((foster* OR promot* OR enhanc* OR engag* OR intervention* OR counselling OR motivat* OR reinforc* OR support* OR 'cognitive behavio*' OR therap* OR program* OR psych*) NEAR/3 (behavio* OR health* OR physical OR lifestyle OR change OR recover*)):ti,ab) OR walk*:ti,ab OR rehabilitat*:ti,ab OR prehabilitat*:ti,ab OR flexib*:ti,ab OR kinesiophob*:ti,ab OR 'physical therap*':ti,ab OR 'physiotherap*':ti,ab OR 'magnetic field therapy':ti,ab OR 'magnetotherapy':ti,ab OR 'transcutaneous electrical nerve stimulation':ti,ab OR tens:ti,ab OR chiropract*:ti,ab OR exerci*:ti,ab OR hydrotherap*:ti,ab OR 'massage therap*':ti,ab OR 'lifestyle change*':ti,ab OR 'lifestyle modif*':ti,ab OR 'aerobic activit*':ti,ab OR pedomet*:ti,ab OR 'behaviour change'/exp OR 'lifestyle modification'/exp OR 'physiotherapy'/de OR 'transcutaneous electrical nerve stimulation'/exp OR 'chiropractor'/exp OR 'chiropractic'/exp OR 'exercise'/exp OR 'massage'/exp OR 'rehabilitation'/exp OR 'pedometer'/exp OR 'prehabilitation'/exp OR 'flexibility'/exp OR 'kinesiophobia'/exp OR 'magnetotherapy'/exp) AND ('randomized controlled trial' OR randomized:ab OR randomised:ab OR placebo:ab OR randomly:ab OR trial:ab OR groups:ab) NOT 'nonhuman'/exp AND [04-07-2024]/sd NOT [29-08-2025]/sd | 997 |

Medline @ Ovid

July 3, 2024

| No. | Search String | Results |
| --- | --- | --- |
| 1 | ((thoracolumbar or lumbar or thoracic) adj2 (surger* or fusion* or pedicle* or discectom* or diskectom* or procedure* or revision* or 'post operative' or 'postoperative' or correction* or endoscop* or microsurg* or microendoscop* or microdiscectom* or microdiskectom* or fixation* or replacement* or arthroplast* or arthrodesis or 'minimally invasive')).ab,ti. | 44419 |
| 2 | ((posterior adj2 (surger* or fusion* or pedicle* or discectom* or diskectom* or procedure* or revision* or 'post operative' or 'postoperative' or correction* or endoscop* or microsurg* or microendoscop* or microdiscectom* or microdiskectom* or fixation* or replacement* or arthroplast* or arthrodesis or 'minimally invasive')) and (spine or spinal)).ab,ti. | 9776 |
| 3 | ((spin* or lumb* or thoracic) adj3 (stenosis or spondyl*)).ab,ti. | 15414 |
| 4 | lss.ab,ti. | 3574 |
| 5 | ((disc or disk or vertebra* or intervertebra*) adj2 (herniat* or protrusion* or lesion* or degenerat* or displace*)).ab,ti. | 30796 |
| 6 | spinal diseases/ or exp intervertebral disc degeneration/ or exp intervertebral disc displacement/ or exp spinal stenosis/ or exp spondylitis/ or exp spondylosis/ | 103769 |
| 7 | 3 or 4 or 5 or 6 | 123517 |
| 8 | surg*.ab,ti. | 2569028 |
| 9 | fusion*.ab,ti. | 282189 |
| 10 | pedicle*.ab,ti. | 39661 |
| 11 | discectom*.ab,ti. | 10102 |
| 12 | diskectom*.ab,ti. | 849 |
| 13 | revision*.ab,ti. | 130880 |
| 14 | post operative.ab,ti. | 87331 |
| 15 | postoperative.ab,ti. | 664563 |
| 16 | preoperative.ab,ti. | 356733 |
| 17 | correction*.ab,ti. | 324923 |
| 18 | decompression*.ab,ti. | 51552 |
| 19 | presurg*.ab,ti. | 13376 |
| 20 | microendoscop*.ab,ti. | 1065 |
| 21 | microdiscectom*.ab,ti. | 1308 |
| 22 | microsurg*.ab,ti. | 32572 |
| 23 | microdiskectom*.ab,ti. | 101 |
| 24 | replacement*.ab,ti. | 332608 |
| 25 | arthroplast*.ab,ti. | 98729 |
| 26 | arthrodesis.ab,ti. | 14683 |
| 27 | minimally invasive.ab,ti. | 106573 |
| 28 | exp Surgical Procedures, Operative/ | 3751614 |
| 29 | 8 or 9 or 10 or 11 or 12 or 13 or 14 or 15 or 16 or 17 or 18 or 19 or 20 or 21 or 22 or 23 or 24 or 25 or 26 or 27 or 28 | 5993192 |
| 30 | 7 and 29 | 50158 |
| 31 | 1 or 2 or 30 | 90389 |
| 32 | ((foster* or promot* or enhanc* or engag* or intervention* or counselling or motivat* or reinforc* or support* or 'cognitive behavio*' or therap* or program* or psych*) adj3 (behavio* or health* or physical or lifestyle or change or recover*)).ab,ti. | 709137 |
| 33 | walk*.ab,ti. | 167021 |
| 34 | rehabilitat*.ab,ti. | 239683 |
| 35 | prehabilitat*.ab,ti. | 2239 |
| 36 | flexib*.ab,ti. | 276025 |
| 37 | kinesiophob*.ab,ti. | 2191 |
| 38 | physical therap*.ab,ti. | 33310 |
| 39 | physiotherap*.ab,ti. | 39366 |
| 40 | magnetic field therapy.ab,ti. | 123 |
| 41 | magnetotherapy.ab,ti. | 327 |
| 42 | transcutaneous electrical nerve stimulation.ab,ti. | 2751 |
| 43 | tens.ab,ti. | 22352 |
| 44 | chiropract*.ab,ti. | 7063 |
| 45 | exerci*.ab,ti. | 398735 |
| 46 | hydrotherap*.ab,ti. | 1263 |
| 47 | massage therap*.ab,ti. | 1785 |
| 48 | lifestyle change*.ab,ti. | 13029 |
| 49 | lifestyle modif*.ab,ti. | 11551 |
| 50 | aerobic activit*.ab,ti. | 993 |
| 51 | pedomet*.ab,ti. | 3273 |
| 52 | exp Physical Therapy Modalities/ | 195305 |
| 53 | exp Exercise/ | 275676 |
| 54 | rehabilitation/ or dance therapy/ or early ambulation/ or exercise therapy/ or neurological rehabilitation/ or occupational therapy/ or recreation therapy/ or rehabilitation, vocational/ or telerehabilitation/ | 104350 |
| 55 | 32 or 33 or 34 or 35 or 36 or 37 or 38 or 39 or 40 or 41 or 42 or 43 or 44 or 45 or 46 or 47 or 48 or 49 or 50 or 51 or 52 or 53 or 54 | 1897128 |
| 56 | ((randomized controlled trial or controlled clinical trial).pt. or randomized.ab. or randomised.ab. or placebo.ab. or drug therapy.fs. or randomly.ab. or trial.ab. or groups.ab.) not (exp animals/ not humans.sh.) | 5621038 |
| 57 | 31 and 55 and 56 | 2397 |
| 58 | limit 57 to ed=20240703-20250829 | 199 |

PsycINFO @ EBSCO

September 2, 2025

| # | Query | Results |
| --- | --- | --- |
| S101 | S55 AND S99 | 25 |
| S100 | S98 AND S99 | 80 |
| S99 | ( PT("randomized controlled trial") OR AB(randomized OR randomised OR placebo OR randomly OR trial OR groups) ) NOT (MH "Animals+") | 1,232,134 |
| S98 | S56 OR S57 OR S58 OR S59 OR S60 OR S61 OR S62 OR S63 OR S64 OR S65 OR S66 OR S67 OR S68 OR S69 OR S70 OR S71 OR S72 OR S73 OR S74 OR S75 OR S76 OR S77 OR S78 OR S79 OR S80 OR S81 OR S82 OR S83 OR S84 OR S85 OR S86 OR S87 OR S88 OR S89 OR S90 OR S91 OR S92 OR S93 OR S94 OR S95 OR S96 OR S97 | 965,972 |
| S97 | DE "Rehabilitation" OR DE "Cognitive Rehabilitation" OR DE "Criminal Rehabilitation" OR DE "Neuropsychological Rehabilitation" OR DE "Neurorehabilitation" OR DE "Occupational Therapy" OR DE "Physical Therapy" OR DE "Psychosocial Rehabilitation" OR DE "Rehabilitation Centers" OR DE "Telerehabilitation" | 50,392 |
| S96 | DE "Behavior Modification" OR DE "Behavior Contracting" OR DE "Behavior Therapy" OR DE "Biofeedback Training" OR DE "Classroom Behavior Modification" OR DE "Contingency Management" OR DE "Fading (Conditioning)" OR DE "Omission Training" OR DE "Overcorrection" OR DE "Self-Management" OR DE "Time Out" OR DE "Behavior Change" | 65,834 |
| S95 | AB pedomet* | 977 |
| S94 | TI pedomet* | 182 |
| S93 | AB "aerobic activit*" | 202 |
| S92 | TI "aerobic activit*" | 26 |
| S91 | AB "lifestyle modif*" | 917 |
| S90 | TI "lifestyle modif*" | 151 |
| S89 | AB "lifestyle change*" | 2,619 |
| S88 | TI "lifestyle change*" | 304 |
| S87 | AB "massage therap*" | 432 |
| S86 | TI "massage therap*" | 155 |
| S85 | AB hydrotherap* | 136 |
| S84 | TI hydrotherap* | 29 |
| S83 | AB exerci* | 78,961 |
| S82 | TI exerci* | 20,131 |
| S81 | AB chiropract* | 480 |
| S80 | TI chiropract* | 127 |
| S79 | AB tens | 260,909 |
| S78 | TI tens | 6,449 |
| S77 | AB "transcutaneous electrical nerve stimulation" | 344 |
| S76 | TI "transcutaneous electrical nerve stimulation" | 171 |
| S75 | AB "magnetotherapy" | 14 |
| S74 | TI "magnetotherapy" | 7 |
| S73 | AB "magnetic field therapy" | 4 |
| S72 | TI "magnetic field therapy" | 2 |
| S71 | AB "physiotherap*" | 4,138 |
| S70 | TI "physiotherap*" | 781 |
| S69 | AB "physical therap*" | 4,341 |
| S68 | TI "physical therap*" | 1,111 |
| S67 | AB kinesiophob* | 362 |
| S66 | TI kinesiophob* | 80 |
| S65 | AB flexib* | 62,771 |
| S64 | TI flexib* | 7,526 |
| S63 | AB prehabilitat* | 55 |
| S62 | TI prehabilitat* | 31 |
| S61 | AB rehabilitat* | 63,798 |
| S60 | TI rehabilitat* | 20,802 |
| S59 | AB walk* | 31,196 |
| S58 | TI walk* | 6,342 |
| S57 | AB ((foster* OR promot* OR enhanc* OR engag* OR intervention* OR counselling OR motivat* OR reinforc* OR support* OR 'cognitive behavio*' OR therap* OR program* OR psych*) N3 (behavio* OR health* OR physical OR lifestyle OR change OR recover*)) | 467,486 |
| S56 | TI ((foster* OR promot* OR enhanc* OR engag* OR intervention* OR counselling OR motivat* OR reinforc* OR support* OR 'cognitive behavio*' OR therap* OR program* OR psych*) N3 (behavio* OR health* OR physical OR lifestyle OR change OR recover*)) | 97,718 |
| S55 | S1 OR S2 OR S3 OR S4 OR S54 | 684 |
| S54 | S11 AND S53 | 288 |
| S53 | S12 OR S13 OR S14 OR S15 OR S16 OR S17 OR S18 OR S19 OR S20 OR S21 OR S22 OR S23 OR S24 OR S25 OR S26 OR S27 OR S28 OR S29 OR S30 OR S31 OR S32 OR S33 OR S34 OR S35 OR S36 OR S37 OR S38 OR S39 OR S40 OR S41 OR S42 OR S43 OR S44 OR S45 OR S46 OR S47 OR S48 OR S49 OR S50 OR S51 OR S52 | 187,857 |
| S52 | DE "Surgery" OR DE "Postsurgical Complications" OR DE "Sterilization (Sex)" OR DE "Transection" OR DE "Amputation" OR DE "Bariatric Surgery" OR DE "Circumcision" OR DE "Cochlear Implants" OR DE "Colostomy" OR DE "Dental Surgery" OR DE "Endocrine Gland Surgery" OR DE "Gender Reassignment" OR DE "Heart Surgery" OR DE "Hysterectomy" OR DE "Induced Abortion" OR DE "Neurosurgery" OR DE "Organ Transplantation" OR DE "Plastic Surgery" OR DE "Stereotaxic Techniques" | 39,828 |
| S51 | AB "minimally invasive" | 766 |
| S50 | TI "minimally invasive" | 109 |
| S49 | AB arthrodesis | 17 |
| S48 | TI arthrodesis | 4 |
| S47 | AB arthroplast* | 612 |
| S46 | TI arthroplast* | 415 |
| S45 | AB replacement* | 13,884 |
| S44 | TI replacement* | 2,354 |
| S43 | AB microdiskectom* | 1 |
| S42 | TI microdiskectom* | 1 |
| S41 | AB microdiscectom* | 15 |
| S40 | TI microdiscectom* | 8 |
| S39 | AB microsurg* | 232 |
| S38 | TI microsurg* | 39 |
| S37 | AB microendoscop* | 25 |
| S36 | TI microendoscop* | 3 |
| S35 | AB presurg* | 1,697 |
| S34 | TI presurg* | 247 |
| S33 | AB decompression* | 728 |
| S32 | TI decompression* | 176 |
| S31 | AB correction* | 52,582 |
| S30 | TI correction* | 14,013 |
| S29 | AB preoperative | 4,427 |
| S28 | TI preoperative | 842 |
| S27 | AB postoperative | 8,629 |
| S26 | TI postoperative | 2,076 |
| S25 | AB "post operative" | 1,854 |
| S24 | TI "post operative" | 326 |
| S23 | AB revision* | 22,104 |
| S22 | TI revision* | 3,663 |
| S21 | AB diskectom* | 5 |
| S20 | TI diskectom* | 2 |
| S19 | AB discectom* | 72 |
| S18 | TI discectom* | 28 |
| S17 | AB pedicle* | 107 |
| S16 | TI pedicle* | 20 |
| S15 | AB fusion* | 9,459 |
| S14 | TI fusion* | 2,409 |
| S13 | AB surg* | 56,443 |
| S12 | TI surg* | 12,375 |
| S11 | (S10 OR S9 OR S8 OR S7 OR S6 OR S5 OR S4 OR S3 OR S2 OR S1) | 997 |
| S10 | AB ((disc OR disk OR vertebra* OR intervertebra*) N2 (herniat* OR protrusion* OR lesion* OR degenerat* OR displace*)) | 406 |
| S9 | TI ((disc OR disk OR vertebra* OR intervertebra*) N2 (herniat* OR protrusion* OR lesion* OR degenerat* OR displace*)) | 115 |
| S8 | AB lss | 401 |
| S7 | TI lss | 17 |
| S6 | AB ((spin* OR lumb* OR thoracic) N3 (stenosis OR spondyl*)) | 234 |
| S5 | TI ((spin* OR lumb* OR thoracic) N3 (stenosis OR spondyl*)) | 74 |
| S4 | AB ((posterior N2 (surger* OR fusion* OR pedicle* OR discectom* OR diskectom* OR procedure* OR revision* OR 'post operative' OR 'postoperative' OR correction* OR endoscop* OR microsurg* OR microendoscop* OR microdiscectom* OR microdiskectom* OR fixation* OR replacement* OR arthroplast* OR arthrodesis OR 'minimally invasive'))) AND (spine OR spinal) | 40 |
| S3 | TI ((posterior N2 (surger* OR fusion* OR pedicle* OR discectom* OR diskectom* OR procedure* OR revision* OR 'post operative' OR 'postoperative' OR correction* OR endoscop* OR microsurg* OR microendoscop* OR microdiscectom* OR microdiskectom* OR fixation* OR replacement* OR arthroplast* OR arthrodesis OR 'minimally invasive'))) AND (spine OR spinal) | 6 |
| S2 | AB ((thoracolumbar OR lumbar OR thoracic) N2 (surger* OR fusion* OR pedicle* OR discectom* OR diskectom* OR procedure* OR revision* OR 'post operative' OR 'postoperative' OR correction* OR endoscop* OR microsurg* OR microendoscop* OR microdiscectom* OR microdiskectom* OR fixation* OR replacement* OR arthroplast* OR arthrodesis OR 'minimally invasive')) | 410 |
| S1 | TI ((thoracolumbar OR lumbar OR thoracic) N2 (surger* OR fusion* OR pedicle* OR discectom* OR diskectom* OR procedure* OR revision* OR 'post operative' OR 'postoperative' OR correction* OR endoscop* OR microsurg* OR microendoscop* OR microdiscectom* OR microdiskectom* OR fixation* OR replacement* OR arthroplast* OR arthrodesis OR 'minimally invasive')) | 170 |

CENTRAL @ Cochrane Library

September 2, 2025

| ID | Search | Hits |
| --- | --- | --- |
| #1 | ((thoracolumbar or lumbar or thoracic) NEAR/2 (surger* or fusion* or pedicle* or discectom* or diskectom* or procedure* or revision* or 'post operative' or 'postoperative' or correction* or endoscop* or microsurg* or microendoscop* or microdiscectom* or microdiskectom* or fixation* or replacement* or arthroplast* or arthrodesis or 'minimally invasive')):ab,ti | 7816 |
| #2 | ((posterior NEAR/2 (surger* or fusion* or pedicle* or discectom* or diskectom* or procedure* or revision* or 'post operative' or 'postoperative' or correction* or endoscop* or microsurg* or microendoscop* or microdiscectom* or microdiskectom* or fixation* or replacement* or arthroplast* or arthrodesis or 'minimally invasive')) and (spine or spinal)):ab,ti | 862 |
| #3 | ((spin* or lumb* or thoracic) NEAR/3 (stenosis or spondyl*)):ab,ti | 2271 |
| #4 | lss:ab,ti | 458 |
| #5 | ((disc or disk or vertebra* or intervertebra*) NEAR/2 (herniat* or protrusion* or lesion* or degenerat* or displace*)):ab,ti | 4079 |
| #6 | MeSH descriptor: [Spinal Diseases] this term only | 604 |
| #7 | MeSH descriptor: [Intervertebral Disc Degeneration] explode all trees | 602 |
| #8 | MeSH descriptor: [Intervertebral Disc Displacement] explode all trees | 1410 |
| #9 | MeSH descriptor: [Spinal Stenosis] explode all trees | 699 |
| #10 | MeSH descriptor: [undefined] explode all trees | 0 |
| #11 | MeSH descriptor: [Spondylolisthesis] explode all trees | 341 |
| #12 | #3 OR #4 OR #5 OR #6 OR #7 OR #8 OR #9 OR #10 OR #11 | 7427 |
| #13 | surg*:ab,ti | 299269 |
| #14 | fusion*:ab,ti | 8660 |
| #15 | pedicle*:ab,ti | 1659 |
| #16 | discectom*:ab,ti | 1680 |
| #17 | diskectom*:ab,ti | 75 |
| #18 | revision*:ab,ti | 5776 |
| #19 | post operative:ab,ti | 35653 |
| #20 | preoperative:ab,ti | 46795 |
| #21 | postoperative:ab,ti | 148712 |
| #22 | correction*:ab,ti | 19756 |
| #23 | decompression*:ab,ti | 3690 |
| #24 | microendoscop*:ab,ti | 83 |
| #25 | microsurg*:ab,ti | 1205 |
| #26 | microdiscectom*:ab,ti | 308 |
| #27 | microdiskectom*:ab,ti | 19 |
| #28 | replacement*:ab,ti | 32957 |
| #29 | arthroplast*:ab,ti | 14830 |
| #30 | arthrodesis:ab,ti | 516 |
| #31 | minimally invasive:ab,ti | 9244 |
| #32 | MeSH descriptor: [Surgical Procedures, Operative] explode all trees | 174993 |
| #33 | #13 OR #14 OR #15 OR #16 OR #17 OR #18 OR #19 OR #20 OR #21 OR #22 OR #23 OR #24 OR #25 OR #26 OR #27 OR #28 OR #29 OR #30 OR #31 OR #32 | 475628 |
| #34 | #12 AND #33 | 4637 |
| #35 | #1 OR #2 OR #34 | 11230 |
| #36 | ((foster* or promot* or enhanc* or engag* or intervention* or counselling or motivat* or reinforc* or support* or 'cognitive behavio*' or therap* or program* or psych*) NEAR/3 (behavio* or health* or physical or lifestyle or change or recover*)):ab,ti | 237490 |
| #37 | walk*:ab,ti | 46341 |
| #38 | rehabilitat*:ab,ti | 55336 |
| #39 | prehabilitat*:ab,ti | 963 |
| #40 | flexib*:ab,ti | 19937 |
| #41 | kinesiophob*:ab,ti | 1497 |
| #42 | physical therap*:ab,ti | 52882 |
| #43 | physiotherap*:ab,ti | 20750 |
| #44 | magnetic field therapy:ab,ti | 1035 |
| #45 | magnetotherapy:ab,ti | 107 |
| #46 | transcutaneous electrical nerve stimulation:ab,ti | 3638 |
| #47 | tens:ab,ti | 3397 |
| #48 | chiropract*:ab,ti | 1236 |
| #49 | exerci*:ab,ti | 141327 |
| #50 | hydrotherap*:ab,ti | 500 |
| #51 | massage therap*:ab,ti | 3962 |
| #52 | lifestyle change*:ab,ti | 15433 |
| #53 | lifestyle modif*:ab,ti | 6762 |
| #54 | aerobic activit*:ab,ti | 7950 |
| #55 | pedomet*:ab,ti | 2169 |
| #56 | MeSH descriptor: [Physical Therapy Modalities] explode all trees | 41711 |
| #57 | MeSH descriptor: [Exercise] explode all trees | 40660 |
| #58 | MeSH descriptor: [Rehabilitation] explode all trees | 57296 |
| #59 | #36 OR #37 OR #38 OR #39 OR #40 OR #41 OR #42 OR #43 OR #44 OR #45 OR #46 OR #47 OR #48 OR #49 OR #50 OR #51 OR #52 OR #53 OR #54 OR #55 OR #56 OR #57 OR #58 | 460103 |
| #60 | #35 AND #59 with Cochrane Library publication date Between Jul 2024 and Sep 2025, in Trials | 245 |
