# Supplementary material for: Effects of rehabilitation and behavior change interventions on physical capacity and physical activity behavior following lumbar surgery for degenerative disease: A systematic review and meta-analysis
Source: PLoS One. 2026 Apr 20;21(4):e0347420. doi: 10.1371/journal.pone.0347420 (PMC13094952; doi:10.1371/journal.pone.0347420)
Supplement: S2 Table — (DOCX) [file pone.0347420.s004.docx]

S2 Table. Reasons for study exclusion (ordered by study reference).

| Study | Reason for exclusion |
| --- | --- |
| Abbott, A. Physiotherapeutic rehabilitation and lumbar fusion surgery. Karolinska Institutet. Thesis. 2010. https://hdl.handle.net/10616/38375 | Wrong publication type |
| Akkaya H, Ayhan H. The effect of in-bed turning and mobilization training given to patients who undergo lumbar disk surgery on postoperative first mobility level: a single-blind, randomized controlled trial. Int J Orthop Trauma Nurs. 2023;50:101022. doi:10.1016/j.ijotn.2023.101022 | Wrong intervention |
| Alaranta H, Hurme M, Einola S, Kallio V, Knuts LR, Törmä T. Rehabilitation after surgery for lumbar disc herniation: results of a randomized clinical trial. Int J Rehabil Res. 1986;9(3):247-57. doi:10.1097/00004356-198609000-00004. | Wrong outcomes |
| Aldemir K, Gürkan A. The effect of pedometer-supported walking and telemonitoring after disc hernia surgery on pain and disability levels and quality of life. Int J Nurs Pract. 2021;27(2):e12917. doi:10.1111/ijn.12917 | Wrong outcomes |
| Archer KR, Master H, Coronado RA, Pennings JS, Cole K, Hymel A, Priest A, Oleisky E, Whitaker S, Sullivan A, et al. Physical activity intervention to improve surgical spine outcomes (PASS trial). Spine J. 2024;24(9 Suppl):S38-9. doi:10.1016/j.spinee.2024.06.515. | Wrong publication type |
| Barbosa L, Frazão C. Impact of demonstration in a realistic simulation environment as a postoperative education in patients’ experience. Einstein (Sao Paulo). 2020;18:eAO4831. doi:10.31744/einstein_journal/2020AO4831 | Wrong patient population |
| Byungho JK, Taeyeong K, Junghoon A, Heecheol C, Dongyun K, Bumchul Y. Manipulative rehabilitation applied soon after lumbar disc surgery improves late post-operative functional disability: a preliminary 2-year follow-up study. J Back Musculoskelet Rehabil. 2017;30(5):999‐1004. doi:10.3233/BMR-169546 | Wrong patient population |
| Cai J, Wang G, Zhao H. Effects of rehabilitation nursing on minimally invasive surgery and the application of long-term activity function in patients with intervertebral disc herniation. Int J Clin Exp Med. 2019;12(6):7418‐25. | Wrong outcomes |
| Coronado R, Ehde D, Pennings J, et al. Psychosocial Mechanisms of Cognitive-Behavioral-Based Physical Therapy Outcomes After Spine Surgery: preliminary Findings From Mediation Analyses. Phys Ther. 2020;100(10):1793‐804. doi:10.1093/ptj/pzaa112 | Wrong outcomes |
| Coronado R, Master H, Bley J, et al. 136. Influence of goal attainment scaling on cognitive behavioral based physical therapy outcomes after lumbar spine surgery. Spine J. 2021;21(9):S68. doi:10.1016/j.spinee.2021.05.164 | Wrong publication type |
| Coronado R, Master H, Bley J, et al. Patient-Centered Goals after Lumbar Spine Surgery: a Secondary Analysis of Cognitive-Behavioral-Based Physical Therapy Outcomes from a Randomized Controlled Trial. Phys Ther. 2022;102(9). doi:10.1093/ptj/pzac091 | Wrong outcomes |
| Danazumi MS, Nuhu JM, Ibrahim SU, et al. Effects of spinal manipulation or mobilization as an adjunct to neurodynamic mobilization for lumbar disc herniation with radiculopathy: a randomized clinical trial. J Man Manip Ther. 2023;31(6):408-20. doi:10.1080/10669817.2023.2192975 | Wrong patient population |
| Duculan R, Cammisa F, Sama A, et al. 190. Sustained improvement in functional status in an RCT to increase lifestyle physical activity after lumbar fusion. Spine J. 2023;23(9):S97‐S98. doi:10.1016/j.spinee.2023.06.213 | Wrong publication type |
| Duculan R, Rigaud M, Cammisa F, et al. 195. Fostering physical activity after complex lumbar spine surgery: long-term results of a randomized trial. Spine J. 2020;20(9):S96‐S97. doi:10.1016/j.spinee.2020.05.606 | Wrong publication type |
| Elsayyad MM, Abdel-Aal NM, Helal ME. Effect of Adding Neural Mobilization Versus Myofascial Release to Stabilization Exercises after Lumbar Spine Fusion: A Randomized Controlled Trial. Arch Phys Med Rehabil. 2021;102(2):251-60. doi:10.1016/j.apmr.2020.07.009 | Wrong outcomes |
| Fors M, Enthoven P, Abbott A, Öberg B. Effects of pre-surgery physiotherapy on walking ability and lower extremity strength in patients with degenerative lumbar spine disorder: secondary outcomes of the PREPARE randomised controlled trial. BMC Musculoskelet Disord. 2019;20(1):468. doi:10.1186/s12891-019-2850-3 | Wrong outcomes |
| Fritz JM, Rhon DI, Garland EL, et al. The Effectiveness of a Mindfulness-Based Intervention Integrated with Physical Therapy (MIND-PT) for Postsurgical Rehabilitation After Lumbar Surgery: A Protocol for a Randomized Controlled Trial as Part of the Back Pain Consortium (BACPAC) Research Program. Pain Med. 2023;24(S1):115-25. | Wrong study design |
| Grzegorz S, Andzelina WN, Agnieszka G, Lukasz G, Andrzej M. Change in psychophysical performance in patients after lumbar discectomy depending on the postoperative rehabilitation program. J Clin Med. 2024;2024:90. | Wrong publication type |
| Guo X, Hou X, Ding S, Chang S. Rehabilitation nursing for patient rehabilitation after minimally invasive spine surgery. Int J Clin Exp Med. 2019;12(3):2450‐5. | Wrong outcomes |
| Guo Y, Hu X, Li J, Yang L. Effects of a WeChat-based individualized post-discharge rehabilitation program on patients with lumbar fusion surgery. J Back Musculoskelet Rehabil. 2022;35(3):545‐57. doi:10.3233/BMR-200280 | Wrong study design |
| He Q, Zhao J, Fan M, Wang F. Effect of continuous nursing based on wechat platform on postoperative rehabilitation of patients with lumbar disc herniation. Jpn J Nurs Sci. 2021;18(2):e12382. doi:10.1111/jjns.12382 | Wrong outcomes |
| He W, Wang Q, Hu J, et al. A randomized trial on the application of a nurse-led early rehabilitation program after minimally invasive lumbar internal fixation. Ann Palliat Med. 2021;10(9):9820‐9. doi:10.21037/apm-21-2294 | Wrong outcomes |
| Heard JC, D’Antonio ND, Lambrechts MJ, et al. Does physical therapy impact clinical outcomes after lumbar decompression surgery? J Craniovertebral Junction Spine. 2023;14(3):230-5. doi:10.4103/jcvjs.jcvjs_61_23 | Wrong study design |
| Hou J, Yang R, Yang Y, et al. The Effectiveness and Safety of Utilizing Mobile Phone-Based Programs for Rehabilitation After Lumbar Spinal Surgery: Multicenter, Prospective Randomized Controlled Trial. JMIR MHealth UHealth. 2019;7(2):e10201. doi:10.2196/10201 | Wrong outcomes |
| Huysmans E, Goudman L, Coppieters I, et al. Effect of perioperative pain neuroscience education in people undergoing surgery for lumbar radiculopathy: a multicentre randomised controlled trial. Br J Anaesth. 2023;131(3):572‐85. doi:10.1016/j.bja.2023.05.007 | Wrong patient population |
| Jentoft E, Kvåle A, Assmus J, Moen V. Effect of information and exercise programmes after lumbar disc surgery: a randomized controlled trial. Physiother Res Int. 2020;25(4):e1864. doi:10.1002/pri.1864 | Wrong outcomes |
| JPRN-UMIN000057882. The effect of advanced multistage exercise load-based physical therapy after lumbar spinal canal stenosis surgery. 2025 | Wrong publication type |
| Kacar S, Alptekin K, Atilla Alkan S, Oncu J. Comparison of daily living return efficiency of patients with pressure biofeedback and normal exercise program after lumbar disc herniation surgery. Minerva Med. 2020;179(9):512‐9. doi:10.23736/S0393-3660.19.04154-8 | Wrong outcomes |
| Kjellby-Wendt G, Styf J. Early active training after lumbar discectomy. A prospective, randomized, and controlled study. Spine. 1998;23(21):2345-51. doi:10.1097/00007632-199811010-00019 | Wrong outcomes |
| Kulikov AG, Gaydukova TY, Lvova NV, Baymuratova DV. Rehabilitation of patients after spinal surgery: effectiveness of nonthermal techniques of peloidotherapy. *Vopr Kurortol Fizioter Lech Fiz Kult.* 2024;102(4):23-9. doi:10.17116/kurort202410104123 | Wrong language |
| La Rocca G, Orlando V, Galieri G, Mazzucchi E, Pignotti F, Cusumano D, Bazzu P, Olivi A, Sabatino G. Mindfulness vs. physiotherapy vs. medical therapy: uncovering the best postoperative recovery method for low back surgery patients during the COVID-19 pandemic—a single institution's experience. J Pers Med. 2024;14(9):917. doi:10.3390/jpm14090917 | Wrong outcomes |
| LeBlanc L, Moldovan ID, Sabri E, Phan P, Agbi C, Mohammed S, AlKherayf F. Comparing the effects of early versus late exercise intervention on pain and neurodynamic mobility following unilateral lumbar microdiscectomy: a pilot study. Spine (Phila Pa 1976). 2021;46(18):E998‐E1005. doi:10.1097/BRS.0000000000004018. | Wrong study design |
| LeBlanc L, Moldovan I, Ochoa-Sanchez R, et al. Early and late exercise intervention after lumbar microdiscectomy reduces low back pain, fear avoidance, and improve neurodynamic mobility. Can J neurol Sci. 2023;50:S82. doi:10.1017/cjn.2023.187 | Wrong publication type |
| Liang J, Wang L, Song J, et al. The impact of nursing interventions on the rehabilitation outcome of patients after lumbar spine surgery. BMC Musculoskelet Disord. 2024;25(1). doi:10.1186/s12891-024-07419-9 | Wrong outcomes |
| Lombardo S, Hilde G, Smastuen M, Grotle M. Effect of Godelieve Denys-Struyf (GDS) muscle and articulation chain treatment on clinical variables of patients with chronic low back pain and lumbar disc degeneration: a pilot feasibility randomized controlled trial. Pilot Feasibility Stud. 2023;9(1):44. doi:10.1186/s40814-023-01268-4 | Wrong patient population |
| Louw A, Diener I, Landers M, Zimney K, Puentedura E. Three-year follow-up of a randomized controlled trial comparing preoperative neuroscience education for patients undergoing surgery for lumbar radiculopathy. J Spine Surg. 2016;2(4):289‐98. doi:10.21037/jss.2016.12.04 | Wrong patient population |
| Lyu Z, Bai J, Chen S, Liu J, Yu W. Efficacy of lumbar kinetic chain training for staged rehabilitation after percutaneous endoscopic lumbar discectomy. BMC Musculoskelet Disord. 2021;22(1). doi:10.1186/s12891-021-04674-y | Wrong outcomes |
| Mancuso CA, Duculan R, Lafage MC, Cammisa FP, Sama AA, Hughes AP, Lebl DR, Girardi FP. Behavioral intervention to foster healthy lifestyle physical activity after complex lumbar surgery: a randomized controlled trial. *J Bone Joint Surg Am.* 2025. doi:10.2106/JBJS.24.01180 | Wrong patient population |
| Mancuso C, Rigaud M, Duculan R, et al. Fostering physical activity after complex lumbar spine surgery: a randomized trial. Spine J. 2019;28:2761. doi:10.1007/s00586-019-06101-2 | Wrong publication type |
| Marchand AA, Houle M, O’Shaughnessy J, Châtillon C, Descarreaux M. Physical Predictors of Favorable Postoperative Outcomes in Patients Undergoing Laminectomy or Laminotomy for Central Lumbar Spinal Stenosis: secondary Analysis of a Randomized Controlled Trial. Front Neurol. 2022;13:848665. doi:10.3389/fneur.2022.848665 | Wrong study design |
| Marchand AA, Suitner M, O'Shaughnessy J, Châtillon CÉ, Cantin V, Descarreaux M. Feasibility of conducting an active exercise prehabilitation program in patients awaiting spinal stenosis surgery: a randomized pilot study. *Sci Rep.* 2019;9(1):12257. doi:10.1038/s41598-019-48736-7 | Trial results reported elsewhere |
| NCT04144452. Therapeutic Exercises and Educational Sessions After Microdiscectomy for Disc Herniation. Published online 2019. https://www.cochranelibrary.com/central/doi/10.1002/central/CN-02001498/full | Wrong study design |
| Oestergaard L, Christensen F, Bünger C, et al. Does adding case management to standard rehabilitation affect functional ability, pain, or the rate of return to work after lumbar spinal fusion? A randomized controlled trial with two-year follow-up. Clin Rehabil. 2020;34(3):357‐68. doi:10.1177/0269215519897106 | Wrong outcomes |
| Ostelo RWJG, de Vet HCW, Berfelo MW, et al. Effectiveness of behavioral graded activity after first-time lumbar disc surgery: short term results of a randomized controlled trial. Eur Spine J. 2003;12(6):637-44. doi:10.1007/s00586-003-0560-9 | Wrong outcomes |
| Ostelo RWJG, de Vet HCW, Vlaeyen JWS, et al. Behavioral graded activity following first-time lumbar disc surgery: 1-year results of a randomized clinical trial. Spine. 2003;28(16):1757-1765. doi:10.1097/01.brs.0000083317.62258.e6 | Wrong outcomes |
| Paulsen RT, Carreon LY, Andersen M. Patient-reported Outcomes after Surgery for Lumbar Disc Herniation, a Randomized Controlled Trial Comparing the Effects of Referral to Municipal Physical Rehabilitation Versus No Referral. Spine. 2020;45(1):3-9. doi:10.1097/BRS.0000000000003221 | Wrong outcomes |
| Paulsen RT, Carreon LY, Andersen M. Recovery after surgery for lumbar disk herniation, a randomized clinical trial comparing the effect of supervised rehabilitation versus home exercises. Eur Spine J. 2019;28:2770. doi:10.1007/s00586-019-06101-2 | Wrong publication type |
| Paulsen RT, Rasmussen J, Carreon LY, Andersen M. Return to work after surgery for lumbar disc herniation, secondary analyses from a randomized controlled trial comparing supervised rehabilitation versus home exercises. Spine. 2020;20(1):41‐7. doi:10.1016/j.spinee.2019.09.019 | Wrong outcomes |
| Paulsen RT, Sorensen J, Carreon LY, Andersen M. Cost-effectiveness of postoperative rehabilitation after surgery for lumbar disc herniation: an analysis based on a randomized controlled trial. J Neurosurg Spine. 2020;32(5):733‐40. doi:10.3171/2019.11.SPINE19100 | Wrong outcomes |
| Reyes A, Aguilera M, Torres P, Reyes-Ferrada W, Peñailillo L. Effects of neural mobilization in patients after lumbar microdiscectomy due to intervertebral disc lesion. J Bodyw Mov Ther. 2021;25:100‐7. doi:10.1016/j.jbmt.2020.10.023 | Wrong outcomes |
| Rolving N, Nielsen CV, Christensen FB, Holm R, Bünger CE, Oestergaard LG. Does a Preoperative Cognitive-Behavioral Intervention Affect Disability, Pain Behavior, Pain, and Return to Work the First Year After Lumbar Spinal Fusion Surgery? Spine. 2015;40(9):593‐600. doi:10.1097/BRS.0000000000000843 | Wrong outcomes |
| Rolving N, Nielsen CV, Christensen FB, Holm R, Bünger CE, Oestergaard LG. Preoperative cognitive-behavioural intervention improves in-hospital mobilisation and analgesic use for lumbar spinal fusion patients. BMC Musculoskelet Disord. 2016;17(1):217. doi:10.1186/s12891-016-1078-8 | Wrong outcomes |
| Rushton A, Heneghan NR, Calvert M, Heap A, White L, Goodwin PC. Physiotherapy Post Lumbar Discectomy: Prospective Feasibility and Pilot Randomised Controlled Trial. PloS One. 2015;10(11):e0142013. doi:10.1371/journal.pone.0142013 | Wrong outcomes |
| Sadeghpour Ezbarami S, Zarei F, Haghani S. Evaluation of a mobile-application educational intervention on the knowledge, attitude, and practice of patients in postoperative care for lumbar disk herniation surgery: A randomized control trial. SAGE Open Med. 2023;11. doi: 10.1177/20503121231203684 | Wrong outcomes |
| Saha B, Goktas S. The Effect of Computer-Based Training on Self-care and Daily Living Activities in Patients With Lumbar Discectomy Surgery: a Randomized Controlled Study. Comput Inform Nurs. 2021;40(3):147‐53. doi:10.1097/CIN.0000000000000829 | Wrong outcomes |
| Salik Sengul Y, Kaya N, Yalcinkaya G, Kirmizi M, Kalemci O. The effects of the addition of motor imagery to home exercises on pain, disability and psychosocial parameters in patients undergoing lumbar spinal surgery: a randomized controlled trial. Explore (NY) 2021;17(4):334‐9. doi:10.1016/j.explore.2020.02.001 | Wrong outcomes |
| Saraçoglu I, Kaya I, Cingöz I, Emre Aydin H. Preoperative pain neurophysiology education for lumbar radiculopathy: a randomized-controlled trial. Turk J Phys Med Rehabil. 2021;67(3):328‐35. doi:10.5606/tftrd.2021.5495 | Wrong patient population |
| Scarone P, Smeets A, van Kuijk S, van Santbrink H, Peters M, Koetsier E. A randomized controlled TRIal of cognitive BEhavioral therapy for high Catastrophizing in patients undergoing lumbar fusion surgery: the TRIBECA study. BMC Musculoskelet Disord. 2020;21(1):810. doi:10.1186/s12891-020-03826-w | Wrong study design |
| Sharma N, Li H, Aoyagi K, et al. Tailored Yoga Intervention for Postlumbar Spine Surgical Pain Management: a Feasibility Study. J Integr Complement Med. 2024;30(8):753-61. doi:10.1089/jicm.2023.0096 | Wrong outcomes |
| Shaygan M, Zamani M, Jaberi A, Eghbal K, Dehghani A. The impact of physical and psychological pain management training on pain intensity, anxiety and disability in patients undergoing lumbar surgeries. Spine J. 2023;23(5):656‐64. doi:10.1016/j.spinee.2023.01.016 | Wrong outcomes |
| Skrobot W, Liedtke E, Krasowska K, et al. Early Rehabilitation Program and Vitamin D Supplementation Improves Sensitivity of Balance and the Postural Control in Patients after Posterior Lumbar Interbody Fusion: A Randomized Trial. Nutrients. 2019;11(9):2202. doi:10.3390/nu11092202 | Wrong intervention |
| Soffin E, Beckman J, Tseng A, et al. Enhanced Recovery after Lumbar Spine Fusion: a Randomized Controlled Trial to Assess the Quality of Patient Recovery. Anesthesiology. 2020;133(2):350‐63. doi:10.1097/ALN.0000000000003346 | Wrong patient population |
| Soffin E, Beckman J, Tseng A, et al. Enhanced Recovery after Lumbar Spine Fusion: a Randomized Controlled Trial to Assess the Quality of Patient Recovery. Anesthesiology. 2020;133(2):350‐63. doi:10.1097/ALN.0000000000003346 | Wrong patient population |
| Strøm J, Nielsen C, Jørgensen L, Andersen N, Laursen M. A web-based platform to accommodate symptoms of anxiety and depression by featuring social interaction and animated information in patients undergoing lumbar spine fusion: a randomized clinical trial. Spine J. 2019;19(5):827‐39. doi:10.1016/j.spinee.2018.11.011 | Wrong outcomes |
| Sun Z, Qi Y. Application of enhanced recovery after surgery care protocol in the perioperative care of patients undergoing lumbar fusion and internal fixation. J Orthop Surg Res. 2022;17(1):240. doi:10.1186/s13018-022-03099-0 | Wrong outcomes |
| Thornes E, Stendal Robinson H, Moosmayer S, Ekeland A, Vøllestad NK. Low-impact exercise program for patients with symptomatic lumbar spinal stenosis awaiting surgery: a controlled pilot study. Eur J Physiother. 2020;22(2):97-105. doi:10.1080/21679169.2018.1554000 | Wrong setting |
| Uysal E, Cine H, Cetin E. The necessity and timing of exercise after lumbar disc herniation surgery. Eur Rev Med Pharmacol Sci. 2023;27(20):9521‐9. doi:10.26355/eurrev_202310_34125 | Wrong outcomes |
| Van Bogaert W, Huysmans E, Coppieters I, et al. The mediating role of pain cognitions and pain sensitivity in the treatment effect of perioperative pain neuroscience education in people undergoing surgery for lumbar radiculopathy. J Pain. 2024;25(8):104521. doi:10.1016/j.jpain.2024.03.017 | Wrong patient population |
| Wang P, Wang Q, Kong C, et al. Enhanced recovery after surgery (ERAS) program for elderly patients with short-level lumbar fusion. J Orthop Surg. 2020;15(1):299. doi:10.1186/s13018-020-01814-3 | Wrong study design |
| Wang S, Yu H, Zheng L, et al. Randomized controlled trial of overall functional exercise process in perioperative of percutaneous transforaminal endoscopic discectomy. Medicine (Baltimore). 2022;101(52):e32544. doi:10.1097/MD.0000000000032544 | Wrong outcomes |
| Yang P, Feng D, Lou F, et al. Application of rehabilitation nursing in patients accepting minimally invasive spine surgery and its effects on pain and adverse emotions. Int J Clin Exp Med. 2020;13(7):5160-6 | Wrong patient population |
| Yin Y, Cao A. EFFECT OF RESOURCEFULNESS EDUCATION ON POSTOPERATIVE REHABILITATION OF PATIENTS WITH LUMBAR DISC HERNIATION. Acta Medica Mediterr. 2022;38(4):2711-5. doi:10.19193/0393-6384_2022_4_406 | Wrong outcomes |
| Yolgösteren E, Külekcioglu S. The effectiveness of balneotherapy and thermal aquatic exercise in postoperative persistent lumbar pain syndrome. Int J Biometeorol. 2021;65(12):2137‐45. doi:10.1007/s00484-021-02176-z | Wrong patient population |
| Zhang H, Wang Z, Li K. Clinical application of enhanced recovery after surgery in lumbar disk herniation patients undergoing dynamic stabilization and discectomy. J Back Musculoskelet Rehabil. 2022;35(1):47-53. doi:10.3233/BMR-200238 | Wrong study design |
| Zhang R, Zhang SJ, Wang XJ. Postoperative functional exercise for patients who underwent percutaneous transforaminal endoscopic discectomy for lumbar disc herniation. Eur Rev Med Pharmacol Sci. 2021;22:15-22. doi:10.26355/eurrev_201807_15354. | Wrong study design |
| Zhang Y, Liao M, Zhou X, Shi X, Cui P. Study on the optimal starting time for lumbodorsal muscles exercises of patients undergoing posterior lumbar decompression and instrumentation. Honghua Xue Ye Xue Za Zhi. 2024;40(4):272‐8. doi:10.3760/cma.j.cn211501-20230905-00471 | Wrong language |
| Zhen L, Jinzhu B. A prospective study on the application of staged lumbar motion chain rehabilitation based on mckenzie’s technique after lumbar percutaneous transforaminal endoscopic discectomy. Chin J Tissue Eng Res. 2020;25(9):1398‐403. doi:10.3969/j.issn.2095-4344.4008 | Wrong language |
| Zheng T, Han J, Zhang J. Influences of Enhanced Recovery After Surgery on Rehabilitation Effect and Postoperative Pain in Patients with Oblique Lumbar Interbody Fusion. Altern Ther Health Med. 2024;30(1):198‐204 | Wrong outcomes |
| Zhong Y, Ding Y, Fu B, et al. The effectiveness of postoperative exercise based on gait analysis compared with conventional exercise in patients with lumbar spinal stenosis: a randomized clinical trial. J Back Musculoskelet Rehabil. 2023;36(6):1399‐409. doi:10.3233/BMR-220409 | Wrong outcomes |
