# Supplementary material for: Effects of rehabilitation and behavior change interventions on physical capacity and physical activity behavior following lumbar surgery for degenerative disease: A systematic review and meta-analysis
Source: PLoS One. 2026 Apr 20;21(4):e0347420. doi: 10.1371/journal.pone.0347420 (PMC13094952; doi:10.1371/journal.pone.0347420)
Supplement: S3 File — (DOCX) [file pone.0347420.s005.docx]

**S3 File. Contrast tables**

**Contrast Table 1. Exercise vs. usual/minimal care**

| **Study** | **Sample population** | **Intervention treatment** | **Comparison treatment** | **Outcomes** | **Main findings** |
| --- | --- | --- | --- | --- | --- |
| Exercise vs. usual/minimal care | | | | | |
| Abdi (2023) | 90 patients (G1=30, G2= 30, G3=30) with lumbar disc herniation  Mean age (G1=44.2, G2=44.9, G3=42.9)  Female (G1=46.4%, G2=51.7%, G3=50%) | G1: 24 sessions of unsupervised home-based exercise based on pelvic tilt, single knee to chest, double knee to chest, partial sit-up, hamstring stretch, hip flexor stretch, and squat, starting 6 weeks after surgery  G2: 24 sessions of unsupervised home-based exercise based on lying prone, prone on elbows, standing extension, lying flexion, sitting flexion, and standing flexion, starting 6 weeks after surgery | G3: conventional postoperative rehabilitation including early walking and physiotherapy | Trunk flexion endurance (endurance test), and trunk extension endurance (modified Sorensen test), immediately after the end of the intervention (3.5 months after surgery) | The G1 obtained better results in the trunk flexion endurance than G2 and G3  The G2 obtained better results in the trunk extension endurance than G1 and G3 |
| Chen (2015) | 60 patients (G1=29, G2=31) with lumbar stenosis  Mean age (G1=51.8, G2=52.1)  Female (G1=45%, G2=55%) | G1: Preoperative education and postoperative exercises including mobilization, core stability, strengthening, and daily supervised exercise during hospitalization | G2: Usual post-operative care instructions | Walking speed (15-meter walk test), and lower extremity endurance (5-times sit-to-stand test), at discharge, 1, 3, and 6 months after discharge | No between-group differences in any outcome |
| Dolan (2000) | 20 patients (G1=9, G2=11) with prolapsed lumbar disc  Mean age (G1=39.2, G2=42.7)  Female (G1=0%, G2=27%) | G1: Usual postoperative advice including exercise and return to activity during the first 6 weeks after surgery. Additionally, 8 sessions over 4 weeks of supervised aerobic, stretching, and strength and endurance exercises, starting 6 weeks after surgery | G2: Usual postoperative advice regarding exercise and return to activity during the first 6 weeks after surgery | Trunk extension endurance (Sorensen test), immediately, 4 and 10.5 months after the end of intervention (2.5, 6.5, and 13 months after surgery) | No between-group comparisons reported |
| Filiz (2005) | 60 patients (G1=20, G2=20, G3=20) with single level lumbar disc herniation  Mean age (G1=38.2, G2=41.3, G3=40.2)  Female (G1=50%, G2=40%, G3=55%) | G1: 24 sessions of supervised intensive lumbar stabilization exercises, and 4 sessions of postoperative back education, starting 1 month after surgery  G2: 1 session learning ‘classical’ McKenzie and Williams exercises, 24 sessions of self-directed ‘classical’ McKenzie and Williams exercises, and 4 sessions of postoperative back education, starting 1 month after surgery | G3: advice to be as active as possible | Trunk flexion and extension endurance (Ito lumbar trunk muscle endurance test), immediately after the end of the intervention (3 months after surgery) | Improved trunk flexion and extension endurance favoring G1 and G2 over G3 |
| Ilves (2017) | 98 patients (G1=48, G2=50) with spondylolisthesis  Mean age (G1=59, G2=58)  Female (G1=71%, G2=76%) | G1: 12 months of progressive self-directed aerobic and back-specific exercises with fear-avoidance counseling, and advice to increase daily steps, starting 3 months after surgery | G2: Usual care comprising 1 information session 3 months after surgery with instructions for light self-directed exercise 3 times per week | Physical activity (international physical activity questionnaire), immediately after the end of the intervention (15 months after surgery) | No between-group differences  No adverse events related to the intervention |
| Ilves (2022) | 98 patients (G1=48, G2=50) with spondylolisthesis  Mean age (G1=59, G2=58)  Female (G1=71%, G2=76%) | G1: 12 months of progressive self-directed aerobic and back-specific exercises with fear-avoidance counseling, and advice to increase daily steps, starting 3 months after surgery | G2: Usual care comprising 1 information session 3 months after surgery with instructions for light self-directed exercise 3 times per week | Trunk flexion and extension strength (isometric dynamometer), trunk extension endurance (Sorensen test), immediately after the end of the intervention (15 months from surgery) | No between-group differences in any outcomes  No adverse events related to the intervention |
| Janssens (2016) | 25 patients (G1=12, G2=13) with single-level disc herniation  Mean age (G1=46, G2=46)  Female (G1=58%, G2=54%) | G1: 8-15 sessions of supervised, individualized education, ergonomic advice, mobilization, and motor control and neurodynamic exercises, starting 2 weeks after surgery | G2: 12 weeks of usual care including basic ergonomic instruction and advice to stay active, starting 2 weeks after surgery | Lower extremity endurance (5-times sit-to-stand test), 3.5 months after the end of the intervention (6 months after surgery) | No between-group differences |
| Ju (2012) | 14 patients (G1=7, G2=7) with lumbar disc herniation  Mean age (G1=45.2, G2=46.2)  Females (N/A) | G1: 36 sessions of supervised lumbar extension strengthening and progressive resistance exercises, starting approximately 15 days after surgery | G2: 12 weeks of rest | Trunk extension strength (isometric dynamometer), immediately after the end of the intervention (3.5 months after surgery) | No between-group comparisons reported |
| Kernc (2018) | 27 patients (G1=14, G2=13) with one level degenerative isthmic spondylolisthesis or degenerative disc disease (with or without spinal stenosis)  Mean age (G1=60.3, G2=61.1)  Females (G1=64%, G2=31%) | G1: 18 sessions of supervised trunk muscle strength training, including isometric exercises for extension, flexion, and lateral flexion, interferential therapy, strength machine exercises, leg adduction, hip extension, abdominal activation, and static stretching, starting 3 weeks after surgery | G2: usual postoperative care not including exercise or physiotherapy for the first 3 months after surgery | Walking capacity (6-min walking test), lower extremity endurance (30-second chair stand test), and trunk flexion, extension, right and left flexion strength (isometric dynamometer), immediately and 15 months after the end of the intervention (3 and 18 months after surgery) | The G1 obtained statistically better results in walking capacity and trunk strength immediately |
| Kulig (2009) | 98 patients (G1=51, G2=47) with disc protrusion  Mean age (G1=39.2, G2=41.4)  Female (G1=43%, G2=49%) | G1: 36 sessions of supervised back extension strength, resistance training exercises, therapeutic mat and upright exercises, endurance training, and 1 session of back care education, starting 4-6 weeks after surgery | G2: 1 session of back care education 4-6 weeks after surgery | Lower extremity endurance (5-times sit-to-stand test), walking speed (50-foot walk test), walking capacity (5-minute walk test), and trunk extension endurance (modified Sorensen test), immediately after the end of intervention (4-4.5 months after surgery) | Improved walking capacity and trunk extension endurance favoring the G1 compared to G2  No adverse events related to interventions |
| Lindbäck (2018) | 197 patients (G1=99, G2=98) with disc herniation, spinal stenosis, spondylolisthesis, or degenerative disc disease  Mean age (G1=58, G2=61)  Female (G1=54%, G2=52%) | G1: 18 sessions of pre-surgery physiotherapy; a supervised exercise program; and a behavior approach to increased physical activity level and decreased fear-avoidance behavior.  Additionally, postoperative rehabilitation including a self-directed exercise program based on a home exercise program and daily walking | G2: usual care including information and advice to continue physical activity, and postoperative rehabilitation including a self-directed exercise program based on a home-exercise program and daily walking | Physical activity (self-reported questionnaire), 3 months and 1 year after surgery | Patients in the physiotherapy group were more physically active at 3 months and 1 year than patients in the control group  No adverse events were reported |
| Nielsen (2010) | 73 patients (G1=35, G2=38) with degenerative lumbar disease  Median age (G1=48, G2=52)  Female (G1=61%, G2=59%) | G1: 6-8 weeks of self-directed preoperative back and abdominal muscle strengthening and aerobic exercises with patient-controlled epidural analgesia, and protein supplementation, followed by 10 sessions of postoperative rehabilitation and mobilization | G2: Usual preoperative advice, pain management, and inpatient postoperative rehabilitation. The postoperative program included mobilizations and performed the general routine rehabilitation for inpatients for 8 days | Lower extremity endurance (5-times sit-to-stand test), the day before surgery, and 1, 3, and 6 months after the end of the intervention and after surgery | No between-group comparisons reported |
| Yílmaz (2003) | 42 patients (G1=14, G2=14, G3=14) with lumbar disc herniation  Mean age G1=46, G2=41, G3=43)  Female (G1=43%, G2=57%, G3=43%) | G1: 24 sessions of supervised dynamic lumbar stabilization exercise including flexibility and range of motion exercises and individual dynamic lumbar stabilization exercises, starting approximately 1 month after surgery  G2: 24 sessions of self-directed exercises including lumbar flexion and extension, pelvic tilt, and abdominal strengthening exercises, starting approximately 1 month after surgery | G3: no exercise | Trunk flexion and extension endurance (Ito lumbar trunk muscle endurance test), immediately after the end of intervention (3 months after surgery) | Improved trunk flexion and extension endurance favoring G1 and G2 over G3 immediately |

**Contrast Table 2. Supervised exercise vs. self-directed exercise**

| **Study** | **Sample population** | **Intervention treatment** | **Comparison treatment** | **Outcomes** | **Main findings** |
| --- | --- | --- | --- | --- | --- |
| Supervised vs. self-directed exercise | | | | | |
| Choi (2005) | 75 patients (G1=35, G2=40) with single-level disc herniation  Mean age (G1=51.1, G2=42.0)  Female (G1=43%, G2=55%) | G1: 4 weeks of self-directed lumbar conditioning exercises, and 12 weeks of supervised lumbar extensor and limb strengthening exercises, starting 6 weeks after surgery | G2: 16 weeks of self-directed lumbar conditioning exercises, starting 2 weeks after surgery | Trunk extension strength (isometric dynamometer), immediately after the end of the intervention (4.5 months after surgery) | Improved lumbar extension strength favoring the G1 over G2 |
| Filiz (2005) | 60 patients (G1=20, G2=20) with single-level lumbar disc herniation  Mean age (G1=38.2, G2=41.3)  Female (G1=50%, G2=40%) | G1: 24 sessions of supervised intensive lumbar stabilization exercises, and 4 sessions of postoperative back education, starting 1 month after surgery | G2: 1 session learning ‘classical’ McKenzie and Williams exercises, 24 sessions of self-directed ‘classical’ McKenzie and Williams exercises, and 4 sessions of postoperative back education, starting 1 month after surgery | Trunk flexion and extension endurance (Ito lumbar trunk muscle endurance test), immediately after the end of the intervention (3 months after surgery) | Improved trunk flexion and extension endurance favoring G1 over G2 |
| Greenwood (2019) | 52 patients (G1=25, G2=27) with degenerative/congenital conditions  Mean age (G1=55.9, G2=52.6)  Female (G1=54%, G2=59%) | G1: 10 sessions of supervised group education and exercise program including cardiovascular, range of motion, limb, and spine strengthening exercises, peer support, and usual hospital-based postoperative care and advice, starting 3 months after surgery | G2: referral to local physiotherapist comprised of 6 sessions of physiotherapy including individualized exercise-based self-management postoperatively, and usual hospital-based postoperative care and advice | Walking speed (50-yard walk test), and lower extremity endurance (sit-to-stand test), 2 weeks and 6.5 months after the end of the intervention (6 and 12 months after surgery) | No between-group difference in any outcome |
| Huang (2023) | 28 patients (G1=14, G2=14) with lumbar degenerative disease  Mean age (G1=49.6, G2=54.3)  Female (G1=42.8%, G2=50%) | G1: 2 sessions of supervised aquatic exercise training, and 18 sessions of unsupervised home exercise based on general stability exercises, starting 4 weeks after surgery | G2: 30 sessions of unsupervised home exercise based on lumbar stability exercise, starting 4 weeks after surgery | Trunk flexion and extension strength (isometric dynamometer), immediately after the end of the intervention (2.5 months after surgery) | The G1 obtained significant differences from the G2 in trunk extension strength |
| Johannsen (1994) | 27 patients (G1=11, G2=16) with lumbar disc herniation  Median age (G1=39, G2=36)  Female (G1=36%, G2=25%) | G1: 24 sessions of supervised exercise based on the lower back, higher back, buttock, and abdominal strengthening, endurance, and stretching exercises, starting 4-6 weeks after surgery | G2: 1 session of instruction to perform a self-directed 10 minutes of jogging, back, abdomen, hip abductors, adductors, and quadriceps strengthening and stretching exercises program twice per week, starting 4-6 weeks after surgery | Trunk flexion and extension strength (isokinetic dynamometer), immediately and 3 months after the end of the intervention (4 and 7 months after surgery) | The G2 obtained significant differences from the G1 in trunk extension strength immediately |
| Lindbäck (2018) | 197 patients (G1=99, G2=98) with disc herniation, spinal stenosis, spondylolisthesis, or degenerative disc disease  Mean age (G1=58, G2=61)  Female (G1=54%, G2=52%) | G1: 18 sessions of pre-surgery physiotherapy; a supervised exercise program; and a behavior approach to increased physical activity level and decreased fear-avoidance behavior.  Additionally, postoperative rehabilitation including a self-directed exercise program based on a home exercise program and daily walking | G2: usual care including information and advice to continue physical activity, and postoperative rehabilitation including a self-directed exercise program based on a home-exercise program and daily walking | Physical activity (self-reported questionnaire), 3 months and 1 year after surgery | Patients in the G1 were more physically active at 3 months and 1 year than patients in the G2  No adverse events were reported |
| Son (2025) | 40 patients (G1=20, G2=20) with lumbar disc hernation, stenosis, or spondylolisthesis Mean age (G1=59.5, G2=66.41)  Female (G1=65%, G2=60%) | G1: 24 sessions over 12 weeks of supervised exercise including stretching, lower limb strength exercises, balance exercises, cardiorespiratory endurance exercise. In addition, one initial education session | G1: 24 sessions over 12 weeks of self-directed exercise including stretching, lower limb strength exercises, balance exercises, cardiorespiratory endurance exercise. In addition, one initial education session | Trunk flexion and extension strength (dynamometer), trunk flexion and extension endurance (sitting isometric endurance test), walking capacity (6-min walk test), balance (one-leg stance test), immediately after the end of the intervention | G1 obtained significantly better results in walking capacity and balance than G2 |
| Yílmaz (2003) | 42 patients (G1=14, G2=14) with lumbar disc herniation  Mean age G1=46, G2=41)  Female (G1=43%, G2=57% | G1: 24 sessions of supervised dynamic lumbar stabilization exercise including flexibility and range of motion exercises and individual dynamic lumbar stabilization exercises, starting approximately 1 month after surgery | G2: 24 sessions of self-directed exercises including lumbar flexion and extension, pelvic tilt, and abdominal strengthening exercises, starting approximately 1 month after surgery | Trunk flexion and extension endurance (Ito lumbar trunk muscle endurance test), immediately after the end of intervention (3 months after surgery) | Improved trunk flexion and extension endurance favoring G1 over G2 immediately |

**Contrast Table 3. Psychologically informed rehabilitation vs.usual/minimal care**

| **Study** | **Sample population** | **Intervention treatment** | **Comparison treatment** | **Outcomes (time)** | **Main findings** |
| --- | --- | --- | --- | --- | --- |
| Psychologically informed rehabilitation vs. usual/minimal care | | | | | |
| Archer (2016) | 86 patients (G1=43, G2=43) with lumbar degenerative condition  Mean age (G1=56.9, G2=58.4)  Female (G1=58%, G2=53%) | G1: 6 sessions (1 in-person and 5 remote) of cognitive-behavioral-based physical therapy, starting 6 weeks after surgery | G2: 6 sessions (1 in-person and 5 remote) of usual postoperative education, starting 6 weeks after surgery | Lower extremity endurance (5-times sit-to-stand test), and walking speed (10-meter walk test), immediately and 3 months after the end of the intervention (3 and 6 months after surgery) | Improved the lower extremity endurance immediately favoring G1 |
| Kemani (2024) | 118 patients (G1=59, G2=59) with lumbar degenerative disease  Mean age (N/A)  Females (N/A) | G1: 4 sessions of prehabilitation, starting 8 to 12 weeks before surgery. Additionally, there was a half-hour follow-up session via phone two weeks after the surgery. These sessions, based on cognitive-behavioral techniques, focused on encouraging physical activity and addressing psychological risk factors before the surgery. | G2: conventional care based on a single session of information about the postoperative mobilization routine, an introduction to a core exercise program to be initiated the day after surgery, and encouragement to stay active and start the recommended exercises before surgery | Physical activity (steps per day, time spent in sedentary, MVPA, and light PA using an accelerometer), walking capacity (5-min walk test), walking speed (50-foot walk test), 1 week before surgery, 3 and 8 weeks after surgery, and 3, 6, 12, 24 months after surgery | No between-group differences in any outcome  No adverse events were reported |
| Lindbäck (2018) | 197 patients (G1=99, G2=98) with disc herniation, spinal stenosis, spondylolisthesis, or degenerative disc disease  Mean age (G1=58, G2=61)  Female (G1=54%, G2=52%) | G1: 18 sessions of pre-surgery physiotherapy; a supervised exercise program; and a behavior approach to increased physical activity level and decreased fear-avoidance behavior.  Additionally, postoperative rehabilitation including a self-directed exercise program based on a home exercise program and daily walking | G2: usual care including information and advice to continue physical activity, and postoperative rehabilitation including a self-directed exercise program based on a home-exercise program and daily walking | Physical activity (self-reported questionnaire), 3 months and 1 year after surgery | Patients in the G1 were more physically active at 3 months and 1 year than patients in the G2  No adverse events were reported |
| Lotzke (2019) | 118 patients (G1=59, G2=59) with disc herniation, foraminal stenosis, or isthmic spondylolisthesis Mean age (G1=44.8, G2=46.7)  Female (G1=56%, G2=51%) | G1: 4 sessions of person-centered prehabilitation, starting 8-12 weeks before surgery and 1 telephone session 2 weeks after surgery. Sessions included cognitive-behavioral approaches to address psychological risk factors and improve self-efficacy. | G2: 1 preoperative session of usual care including information about post-operative mobilization, core exercises, and advice to stay active after surgery | Physical activity (MVPA, light PA, and sedentary time using an accelerometer), walking capacity (5-minute walk test), walking speed (50-foot walk test), 1 week before and 3 and 6 months after surgery | No between-group differences in any outcome  No adverse events were reported |
| Master (2024) | 16 patients (G1=8, G2=8) with lumbar degenerative disease  Mean age (G1=65.4, G2= 63)  Female (G1=50%, G2=50%) | G1: 8 weekly sessions including motivational interviewing and the use of a wearable watch with supervised daily step goal tracking, starting 2 weeks after surgery | G2: postoperative usual care based on lifting restrictions, advice to stay active, and oral analgesics as needed | Physical activity (number of daily steps, and MVPA using an accelerometer), 2 weeks and 3.5 months after the end of the intervention (3 and 6 months after surgery) | No between-group comparisons reported  No adverse events were reported |
| Tegner (2024) | 144 patients (G1= 74, G2= 70) with lumbar disc-degeneration, spondylosis or spondylolisthesis  Mean age (G1= 54.9, G2= 59.4)  Female (G1= 52.1%, G2= 64.7%) | G1: Usual care starting 1-2 weeks before surgery and continued post-surgery. Additionally, 9 sessions of a cognitive treatment approach that included reinforcement of healthy behaviors, identifying negative emotions and beliefs related to pain, and creating and using more adaptive ones, starting 2 days after surgery | G2: usual care starting 1-2 weeks before surgery and continued after surgery. Preoperative usual care was based on information concerning anesthesia, surgery, medication, and an introduction to mobilization techniques. Postoperative usual care was based on information, mobilization, and instructions to gradually increase movement | Physical activity (sedentary time using an accelerometer), 2 weeks and 9.5 months after the end of the intervention (3 and 12 months after surgery) | G1 showed significantly better results in sedentary time compared to G2 at 9.5 months |

**Contrast Table 4. Physical activity advice vs. usual/minimal care**

| **Study** | **Sample population** | **Intervention treatment** | **Comparison treatment** | **Outcomes (time)** | **Main findings** |
| --- | --- | --- | --- | --- | --- |
| Physical activity advice vs. usual/minimal care | | | | | |
| Ilves (2017) | 98 patients (G1=48, G2=50) with spondylolisthesis  Mean age (G1=59, G2=58)  Female (G1=71%, G2=76%) | G1: 12 months of self-directed aerobic and back-specific exercises with fear-avoidance counseling, and advice to increase daily steps, starting 3 months after surgery | G2: Usual care comprising 1 information session 3 months after surgery with instructions for self-directed exercise 3 times per week | Physical activity (international physical activity questionnaire), immediately after the end of the intervention (15 months after surgery) | No between-group differences  No adverse events related to the intervention |
| Ilves (2022) | 98 patients (G1=48, G2=50) with spondylolisthesis  Mean age (G1=59, G2=58)  Female (G1=71%, G2=76%) | G1: 12 months of self-directed aerobic and back-specific exercises with fear-avoidance counseling, and advice to increase daily steps, starting 3 months after surgery | G2: Usual care comprising 1 information session 3 months after surgery with instructions for self-directed exercise 3 times per week | Trunk flexion and extension strength (isometric dynamometer), trunk extension endurance (Sorensen test), immediately after the end of the intervention (15 months from surgery) | No between-group differences in any outcomes  No adverse events related to the intervention |
| Kemani (2024) | 118 patients (G1=59, G2=59) with lumbar degenerative disease  Mean age (N/A)  Females (N/A) | G1: 4 sessions of prehabilitation, starting 8 to 12 weeks before surgery. Additionally, there was a half-hour follow-up session via phone two weeks after the surgery. These sessions, based on cognitive-behavioral techniques, focused on encouraging physical activity and addressing psychological risk factors before the surgery | G2: conventional care based on a single session of information about the postoperative mobilization routine, an introduction to a core exercise program to be initiated the day after surgery, and encouragement to stay active and start the recommended exercises before surgery | Physical activity (steps per day, time spent in sedentary, MVPA, and light PA using an accelerometer), walking capacity (5-min walk test), walking speed (50-foot walk test), 1 week before surgery, 3 and 8 weeks after surgery, and 3, 6, 12, 24 months after surgery | No between-group differences in any outcome  No adverse events were reported |
| Kjellby-Wendt (2002)  FOLLOW-UP study from 1998 | 52 patients (G1=26, G2=26) with lumbar disc herniation  Mean age (G1=41, G2=39)  Female (G1=31%, G2=23%) | G1: 4 supervised sessions and daily self-directed exercises, starting 1 day after surgery including advice about coping and remaining active, lumbar and leg range of motion exercises, strengthening trunk extensor exercises, spinal stabilization exercises, and intensive cardiovascular exercises | G2: 3 supervised sessions and daily self-directed exercises of less active rehabilitation, starting 1 day after surgery including abdominal and leg strengthening exercises, and spine range of motion exercises | Physical activity (self-reported questionnaire), 5-7 years after the end of the intervention and after surgery | No between-group comparisons reported |
| Lindbäck (2018) | 197 patients (G1=99, G2=98) with disc herniation, spinal stenosis, spondylolisthesis, or degenerative disc disease  Mean age (G1=58, G2=61)  Female (G1=54%, G2=52%) | G1: 18 sessions of pre-surgery physiotherapy; a supervised exercise program; and a behavior approach to increased physical activity level and decreased fear-avoidance behavior.  Additionally, postoperative rehabilitation including a self-directed exercise program based on a home exercise program and daily walking | G2: usual care including information and advice to continue physical activity, and postoperative rehabilitation including a self-directed exercise program based on a home-exercise program and daily walking | Physical activity (self-reported questionnaire), 3 months and 1 year after surgery | Patients in the G1 were more physically active at 3 months and 1 year than patients in the G2  No adverse events were reported |
| Lotzke (2019) | 118 patients (G1=59, G2=59) with disc herniation, foraminal stenosis, or isthmic spondylolisthesis Mean age (G1=44.8, G2=46.7)  Female (G1=56%, G2=51%) | G1: 4 sessions of person-centered prehabilitation, starting 8-12 weeks before surgery and 1 telephone session 2 weeks after surgery. Sessions included cognitive-behavioral approaches to address psychological risk factors and improve self-efficacy. | G2: 1 preoperative session of usual care including information about post-operative mobilization, core exercises, and advice to stay active after surgery | Physical activity (MVPA, light PA, and sedentary time using an accelerometer), walking capacity (5-minute walk test), walking speed (50-foot walk test), 1 week before and 3 and 6 months after surgery | No between-group differences in any outcome  No adverse events were reported |
| Master (2024) | 16 patients (G1=8, G2=8) with lumbar degenerative disease  Mean age (G1=65.4, G2= 63)  Female (G1=50%, G2=50%) | G1: 8 weekly sessions including motivational interviewing and the use of a wearable watch with supervised daily step goal tracking, starting 2 weeks after surgery | G2: postoperative usual care based on lifting restrictions, advice to stay active, and oral analgesics as needed | Physical activity (number of daily steps, and MVPA using an accelerometer), 2 weeks and 3.5 months after the end of the intervention (3 and 6 months after surgery) | No between-group comparisons reported  No adverse events were reported |

**Contrast table 5. Prehabilitation vs. usual/minimal care**

| **Study** | **Sample population** | **Intervention treatment** | **Comparison treatment** | **Outcomes (time)** | **Main findings** |
| --- | --- | --- | --- | --- | --- |
| Prehabilitation vs. usual/minimal care | | | | | |
| Chen (2015) | 60 patients (G1=29, G2=31) with lumbar stenosis  Mean age (G1=51.8, G2=52.1)  Female (G1=45%, G2=55%) | G1: Preoperative education and postoperative exercises including mobilization, core stability, strengthening, and daily supervised exercise during hospitalization | G2: Usual post-operative care instructions | Walking speed (15-meter walk test), and lower extremity endurance (5-times sit-to-stand test), at discharge, 1, 3, and 6 months after discharge | No between-group differences in any outcome |
| Kemani (2024) | 118 patients (G1=59, G2=59) with lumbar degenerative disease  Mean age (N/A)  Females (N/A) | G1: 4 sessions of prehabilitation, starting 8 to 12 weeks before surgery. Additionally, there was a half-hour follow-up session via phone two weeks after the surgery. These sessions, based on cognitive-behavioral techniques, focused on encouraging physical activity and addressing psychological risk factors before the surgery. | G2: conventional care based on a single session of information about the postoperative mobilization routine, an introduction to a core exercise program to be initiated the day after surgery, and encouragement to stay active and start the recommended exercises before surgery. | Physical activity (steps per day, time spent in sedentary, MVPA, and light PA using an accelerometer), walking capacity (5-min walk test), walking speed (50-foot walk test), 1 week before surgery, 3 and 8 weeks after surgery, and 3, 6, 12, 24 months after surgery | No between-group differences in any outcome  No adverse events were reported |
| Lindbäck (2018) | 197 patients (G1=99, G2=98) with disc herniation, spinal stenosis, spondylolisthesis, or degenerative disc disease  Mean age (G1=58, G2=61)  Female (G1=54%, G2=52%) | G1: 18 sessions of pre-surgery physiotherapy; a supervised exercise program; and a behavior approach to increased physical activity level and decreased fear-avoidance behavior.  Additionally, postoperative rehabilitation including a self-directed exercise program based on a home exercise program and daily walking | G2: usual care including information and advice to continue physical activity, and postoperative rehabilitation including a self-directed exercise program based on a home-exercise program and daily walking | Physical activity (self-reported questionnaire), 3 months and 1 year after surgery | Patients in the G1 were more physically active at 3 months and 1 year than patients in the G2  No adverse events were reported |
| Lotzke (2019) | 118 patients (G1=59, G2=59) with disc herniation, foraminal stenosis, or isthmic spondylolisthesis Mean age (G1=44.8, G2=46.7)  Female (G1=56%, G2=51%) | G1: 4 sessions of person-centered prehabilitation, starting 8-12 weeks before surgery and 1 telephone session 2 weeks after surgery. Sessions included cognitive-behavioral approaches to address psychological risk factors and improve self-efficacy | G2: 1 preoperative session of usual care including information about post-operative mobilization, core exercises, and advice to stay active after surgery | Physical activity (MVPA, light PA, and sedentary time using an accelerometer), walking capacity (5-minute walk test), walking speed (50-foot walk test), 1 week before and 3 and 6 months after surgery | No between-group differences in any outcome  No adverse events were reported |
| Marchand (2021) | 68 patients (G1=35, G2=33) with lumbar spinal stenosis  Mean age (G1=66.2, G2=71.6)  Female (G1=40%, G2=42%) | G1: 18 sessions of supervised warm-up (cycling or walking), trunk, hip, and lower extremity muscle strengthening exercises, and standardized written information on postural recommendations, delivered before surgery | G2: Usual preoperative management and advice, and standardized written information on postural recommendations | Trunk extension endurance (modified Sorensen test), trunk flexion and extension strength (isometric dynamometer), lower extremity endurance (30-seconds sit-to-stand test), lower extremity strength (isometric dynamometer), completed pre-surgery (after intervention), and 6 weeks after surgery | G1 had greater trunk flexion strength, trunk extension endurance, and lower extremity endurance after the intervention than G2  No adverse events were reported |
| Nielsen (2010) | 73 patients (G1=35, G2=38) with degenerative lumbar disease  Median age (G1=48, G2=52)  Female (G1=61%, G2=59%) | G1: 6-8 weeks of self-directed preoperative back and abdominal muscle strengthening and aerobic exercises with patient-controlled epidural analgesia, and protein supplementation, followed by 10 sessions of postoperative rehabilitation and mobilization | G2: Usual preoperative advice, pain management, and inpatient postoperative rehabilitation. The postoperative program included mobilizations and performed the general routine rehabilitation for inpatients for 8 days | Lower extremity endurance (5-times sit-to-stand test), the day before surgery, and 1, 3, and 6 months after the end of the intervention and after surgery | No between-group comparisons reported |
| Tegner (2024) | 144 patients (G1= 74, G2= 70) with lumbar disc-degeneration, spondylosis or spondylolisthesis  Mean age (G1= 54.9, G2= 59.4)  Female (G1= 52.1%, G2= 64.7%) | G1: Usual care starting 1-2 weeks before surgery and continued post-surgery. Additionally, 9 sessions of a cognitive treatment approach that included reinforcement of healthy behaviors, identifying negative emotions and beliefs related to pain, and creating and using more adaptive ones, starting 2 days after surgery | G2: usual care starting 1-2 weeks before surgery and continued after surgery. Preoperative usual care was based on information concerning anesthesia, surgery, medication, and an introduction to mobilization techniques. Postoperative usual care was based on information, mobilization, and instructions to gradually increase movement | Physical activity (sedentary time using an accelerometer), 2 weeks and 9.5 months after the end of the intervention (3 and 12 months after surgery) | G1 showed significantly better results in sedentary time compared to G2 at 9.5 months |
| Abbreviations: Disc, discectomy; G1, group 1; G2, group 2; G3, group 3; PA, physical activity; MVPA, moderate to vigorous physical activity; N/A, not available | | | | | |
