# Supplementary material for: Effects of rehabilitation and behavior change interventions on physical capacity and physical activity behavior following lumbar surgery for degenerative disease: A systematic review and meta-analysis
Source: PLoS One. 2026 Apr 20;21(4):e0347420. doi: 10.1371/journal.pone.0347420 (PMC13094952; doi:10.1371/journal.pone.0347420)
Supplement: S4 File — (DOCX) [file pone.0347420.s006.docx]

**S4 File. Risk of Bias Tables**

Article: Abdi (2023)

| **Domain** | **Support for judgment** | **Review authors’ judgement** |
| --- | --- | --- |
| *Selection bias* | | |
| **Random sequence generation** | Quote: “Then they were randomized into three groups using random numbers in a random software.” | Low risk |
| **Allocation concealment** | Comment: Insufficient information. | Unclear risk |
| *Performance bias* | | |
| **Blinding of participants and personnel** | Quote: “Given the nature of the interventions, it was not possible to blind patients but they were asked not to discuss their exercise program with the assessors.” | High risk |
| *Detection bias* | | |
| **Blinding of outcome assessment** | Quote: “The researcher (ZS) involved in all assessments and measurements were blinded to the group allocation.” | Low risk |
| *Attrition bias* | | |
| **Incomplete outcome data** | Quote: “Two subjects from the Williams group and one from the McKenzie group were excluded because of the loss to follow-up. Finally, data analysis was performed on 87 subjects.” | Low risk |
| *Reporting bias* | | |
| **Selective reporting** | Comment Pre-registered clinical trial. Outcome measures: VAS, ODI, mBST, TFET and return to work. | Low risk |
| *Other bias* | | |
| **Other sources of bias** | Comment: No other bias to be assessed. | Low risk |

Article: Archer (2016)

| **Domain** | **Support for judgment** | **Review authors’ judgement** |
| --- | --- | --- |
| *Selection bias* | | |
| **Random sequence generation** | Quote: “A computer-generated scheme randomized patients to either CBPT or education in a 1:1 ratio in blocks of assignments.” | Low risk |
| **Allocation concealment** | Comment: Insufficient information. | Unclear risk |
| *Performance bias* | | |
| **Blinding of participants and personnel** | Quote: “patients were blinded to group assignment. Participants were informed that they would be randomly assigned to 1 of 2 different educational treatments” Comment: The therapists were aware of the intervention they were delivering, which indicates a lack of blinding. | High risk |
| *Detection bias* | | |
| **Blinding of outcome assessment** | Quote: “The investigators, participating surgeons, research personnel conducting the assessments, and patients were blinded to group assignment.” | Low risk |
| *Attrition bias* | | |
| **Incomplete outcome data** | Comment: Dropout rate was low, well reported and similar in both groups. | Low risk |
| *Reporting bias* | | |
| **Selective reporting** | Comment: trail registered with ClinicalTrails.gov: NCT01331611 | Low risk |
| *Other bias* | | |
| **Other sources of bias** | Comment: The study appears to be free from other sources of bias. | Low risk |

Article: Chen (2015)

| **Domain** | **Support for judgment** | **Review authors’ judgement** |
| --- | --- | --- |
| *Selection bias* | | |
| **Random sequence generation** | Quote: “The patients were randomly allocated to either the perioperative group (PG) or the control group (CG) by a health professional who did not take part in the trial and only had patients fill in a baseline questionnaire.”  Comment: Insufficient information | Unclear risk |
| **Allocation concealment** | Comment: insufficient information. | Unclear risk |
| *Performance bias* | | |
| **Blinding of participants and personnel** | Quote: “The two patient groups and their healthcare staff were kept separated during the study period; neither were they allowed to discuss the intervention, nor were the healthcare personnel treating the CG aware of the procedures for the PG.” Comment: The therapists were aware of the intervention they were delivering, which indicates a lack of blinding. | High risk |
| *Detection bias* | | |
| **Blinding of outcome assessment** | Quote: “An independent investigator blinded to group allocation assessed outcome measurements.” | Low risk |
| *Attrition bias* | | |
| **Incomplete outcome data** | Comment: High attrition rate with vague reasons. Intention-to-treat analysis: mean was used to input a dropout patient’s missing data. | High risk |
| *Reporting bias* | | |
| **Selective reporting** | Comment: Insufficient information | Unclear risk |
| *Other bias* | | |
| **Other sources of bias** | Comment: No sample size calculation. | Unclear risk |

Article: Choi (2005)

| **Domain** | **Support for judgment** | **Review authors’ judgement** |
| --- | --- | --- |
| *Selection bias* | | |
| **Random sequence generation** | Quote: “Patients were randomized into two groups, a control group and an exercise group.”  Comment: insufficient information | Unclear risk |
| **Allocation concealment** | Comment: insufficient information. | Unclear risk |
| *Performance bias* | | |
| **Blinding of participants and personnel** | Comment: Unable to blind participants and personnel | High risk |
| *Detection bias* | | |
| **Blinding of outcome assessment** | Comment: insufficient information. | Unclear risk |
| *Attrition bias* | | |
| **Incomplete outcome data** | Quote: “Among the exercise group, five patients dropped out. Two of them experienced increased pain, whereas the other three cited personal reasons. These five thus were excluded from the analyses.” Comment: no dropouts within the control group | High risk |
| *Reporting bias* | | |
| **Selective reporting** | Comment: insufficient information. | Unclear risk |
| *Other bias* | | |
| **Other sources of bias** | Comment: No sample size calculation. | Unclear risk |

Article: Dolan (2000)

| **Domain** | **Support for judgment** | **Review authors’ judgement** |
| --- | --- | --- |
| *Selection bias* | | |
| **Random sequence generation** | Quote: “all patients were blindly randomized into an EXERCISE or CONTROL group” Comment: insufficient information | Unclear risk |
| **Allocation concealment** | Comment: Insufficient information | Unclear risk |
| *Performance bias* | | |
| **Blinding of participants and personnel** | Comment: Unable to blind participants and personnel | High risk |
| *Detection bias* | | |
| **Blinding of outcome assessment** | Comment: insufficient information | Unclear risk |
| *Attrition bias* | | |
| **Incomplete outcome data** | Quote: “One patient withdrew from the study at 6-weeks, before the exercise program, and he was therefore excluded from all analyse”  Comment: no other dropouts or missing data reported | Unclear risk |
| *Reporting bias* | | |
| **Selective reporting** | Comment: insufficient information | Unclear risk |
| *Other bias* | | |
| **Other sources of bias** | Comment: Pilot study (Preliminary results) | Unclear risk |

Article: Filiz (2005)

| **Domain** | **Support for judgment** | **Review authors’ judgement** |
| --- | --- | --- |
| *Selection bias* | | |
| **Random sequence generation** | Comment: pick out of a box randomly | Low risk |
| **Allocation concealment** | Quote: “60 sheets of opaque paper, which were folded with the treatment and taped from the corners (in order to prevent the drawer being able to see the method) and put it in a box.” | Low risk |
| *Performance bias* | | |
| **Blinding of participants and personnel** | Comment: Single blinded study, unable to blind participants and personnel | High risk |
| *Detection bias* | | |
| **Blinding of outcome assessment** | Quote: “The physician who did the evaluation (before and after treatment) was blinded to the treatment.” | Low risk |
| *Attrition bias* | | |
| **Incomplete outcome data** | Comment: No patients were lost of follow up. | Low risk |
| *Reporting bias* | | |
| **Selective reporting** | Comment: Insufficient information | Unclear risk |
| *Other bias* | | |
| **Other sources of bias** | Comment: no sample size calculation, small sample sizes. | Unclear risk |

Article: Greenwood (2019)

| **Domain** | **Support for judgment** | **Review authors’ judgement** |
| --- | --- | --- |
| *Selection bias* | | |
| **Random sequence generation** | Quote: “Block randomisation was employed” | Low risk |
| **Allocation concealment** | Quote: “Codes were generated at the remote host University site and placed into sequentially numbered opaque envelopes to preserve blinding of allocation.” | Low risk |
| *Performance bias* | | |
| **Blinding of participants and personnel** | Quote: “The nature of the intervention prevents the blinding of participants, who were informed of their allocation.” | High risk |
| *Detection bias* | | |
| **Blinding of outcome assessment** | Quote: “Data collection and study physiotherapists were not blinded to allocation. As the majority of outcome measures were self-reported, this potential source of bias was unlikely to have significantly impacted the results.” | High risk |
| *Attrition bias* | | |
| **Incomplete outcome data** | Quote: “Missing data were treated as per protocol, a case wise deletion of the relevant variable and time point, which is acceptable when the data are missing at random.” | High risk |
| *Reporting bias* | | |
| **Selective reporting** | Comment: Study registered ISRCTN60891364 | Low risk |
| *Other bias* | | |
| **Other sources of bias** | Comment: Feasibility study: small sample size | Unclear risk |

Article: Häkkinen (2005)

| **Domain** | **Support for judgment** | **Review authors’ judgement** |
| --- | --- | --- |
| *Selection bias* | | |
| **Random sequence generation** | Quote: “126 patients were randomly assigned” Comment: insufficient information | Unclear risk |
| **Allocation concealment** | Comment: insufficient information | Unclear risk |
| *Performance bias* | | |
| **Blinding of participants and personnel** | Comment: Unable to blind participants and personnel. | High risk |
| *Detection bias* | | |
| **Blinding of outcome assessment** | Quote: “The tests of physical function were performed in the exercise laboratory by a second experienced physiotherapist blinded to the patient’s group assignment.” | Low risk |
| *Attrition bias* | | |
| **Incomplete outcome data** | Quote: “Clinical outcome variables were analyzed by using the intention-  to-treat principle.” Comment: similar dropout rate in both groups with reasons provided. | Low risk |
| *Reporting bias* | | |
| **Selective reporting** | Comment: insufficient information | Unclear risk |
| *Other bias* | | |
| **Other sources of bias** | Comment: no sample size calculation | Unclear risk |

Article: Hebert (2013)

| **Domain** | **Support for judgment** | **Review authors’ judgement** |
| --- | --- | --- |
| *Selection bias* | | |
| **Random sequence generation** | Quote: “A random number generator was used to create a permuted block randomisation list with variable block sizes of 4–6.” | Low risk |
| **Allocation concealment** | Quote: “Sequentially numbered, opaque envelopes containing the participant’s group assignment were prepared by research staff not affiliated with this trial.” | Low risk |
| *Performance bias* | | |
| **Blinding of participants and personnel** | Quote: “Group assignments were concealed from participants and outcome assessors.”  Comment: no information about the blinding of the therapists | High risk |
| *Detection bias* | | |
| **Blinding of outcome assessment** | Quote: “Group assignments were concealed from participants and outcome assessors.” | Low risk |
| *Attrition bias* | | |
| **Incomplete outcome data** | Quote: “Consistent with the intention-to-treat principle, the linear mixed models estimated values for missing data based on the available scores” | Low risk |
| *Reporting bias* | | |
| **Selective reporting** | Quote: “The trial was prospectively registered (ClinicalTrials.gov ID: NCT00894972).” | Low risk |
| *Other bias* | | |
| **Other sources of bias** | Comment: The study appears to be free from other sources of bias. | Low risk |

Article: Huang (2023)

| **Domain** | **Support for judgment** | **Review authors’ judgement** |
| --- | --- | --- |
| *Selection bias* | | |
| **Random sequence generation** | Quote: “The randomization procedure was performed by a third party using block randomization with a block size of 4.” | Low risk |
| **Allocation concealment** | Comment: No information about this process. | Unclear risk |
| *Performance bias* | | |
| **Blinding of participants and personnel** | Comment: Due to the nature of the intervention, the researchers and participants were not blinded. | High risk |
| *Detection bias* | | |
| **Blinding of outcome assessment** | Quote: “The measurement of secondary outcomes and evaluation of exercise intervention were performed by the first author (AH Huang), and the primary outcomes were evaluated by a physiotherapist with 5 years of working experience, who was blinded to the grouping of the participants.” | Low risk |
| *Attrition bias* | | |
| **Incomplete outcome data** | Quote: “All analyses were conducted using the intention-to-treat approach. The missing data from those patients who had left the study during the intervention or before the post-test would be replaced using the pre-test data.” | Low risk |
| *Reporting bias* | | |
| **Selective reporting** | Quote: “Primary outcomes were pain intensity and disability, and the secondary outcomes were mobility, muscle strength, lumbopelvic stability, and LM muscle thickness.” Pre-registered. | Low risk |
| *Other bias* | | |
| **Other sources of bias** | Comment: No other bias. | Low risk |

Article: Ilves (2017)

| **Domain** | **Support for judgment** | **Review authors’ judgement** |
| --- | --- | --- |
| *Selection bias* | | |
| **Random sequence generation** | Quote: “Allocation to LSF-EX or LSF-UC was performed randomly using computer-generated 4-block randomization lists compiled by a biostatistician.” | Low risk |
| **Allocation concealment** | Quote: “Concealed randomization was used, and was conducted by nurses who were not otherwise involved in the study.” | Low risk |
| *Performance bias* | | |
| **Blinding of participants and personnel** | Quote: “The nature of the study meant that it was not possible to blind caregivers and patients.” Quote: “However, owing to the nature of the study, the physiotherapists could not be blinded.” | High risk |
| *Detection bias* | | |
| **Blinding of outcome assessment** | Quote: “The study questionnaires were collected and saved by research assistants who were blinded to treatment.” | Low risk |
| *Attrition bias* | | |
| **Incomplete outcome data** | Quote: “The intention-to-treat method was used and all patients (n = 98) were thus included in the analysis in their original assigned groups.” | Low risk |
| *Reporting bias* | | |
| **Selective reporting** | Comment: trial registration: NCT00834015 | Low risk |
| *Other bias* | | |
| **Other sources of bias** | Comment: The study appears to be free from other sources of bias. | Low risk |

Article: Ilves (2022)

| **Domain** | **Support for judgment** | **Review authors’ judgement** |
| --- | --- | --- |
| *Selection bias* | | |
| **Random sequence generation** | Comment: using the concealed four-block- randomization method compiled by statistician. | Low risk |
| **Allocation concealment** | Comment: No information about this process | Unclear risk |
| *Performance bias* | | |
| **Blinding of participants and personnel** | Quote: “However, the 7 treating physiotherapists could not be blinded because of the nature of the study.” | High risk |
| *Detection bias* | | |
| **Blinding of outcome assessment** | Quote: “The assessors were blinded to the treatment.” | Low risk |
| *Attrition bias* | | |
| **Incomplete outcome data** | Quote: “The outcomes were analyzed using the intention to treat (ITT) principle.” | Low risk |
| *Reporting bias* | | |
| **Selective reporting** | Quote: “This study is a randomized controlled trial (NCT00834015) reporting secondary outcome measures, trunk muscle strength and spinal range of movement. The primary outcomes were published in 2017 [16].” | Low risk |
| *Other bias* | | |
| **Other sources of bias** | Comment: No other concerns | Low risk |

Article: Janssens (2016)

| **Domain** | **Support for judgment** | **Review authors’ judgement** |
| --- | --- | --- |
| *Selection bias* | | |
| **Random sequence generation** | Quote: “First, they were randomly allocated (blinded by computer algorithm) for surgical approach” “Subsequently, the same patients were reallocated into a physiotherapy group and a usual care group.” | Low risk |
| **Allocation concealment** | Comment: insufficient information | Unclear risk |
| *Performance bias* | | |
| **Blinding of participants and personnel** | Comment: Unable to blind participants and personnel | High risk |
| *Detection bias* | | |
| **Blinding of outcome assessment** | Comment: insufficient information | Unclear risk |
| *Attrition bias* | | |
| **Incomplete outcome data** | Comment: low attrition rate but reasons for dropouts not reported and no intention-to-treat analysis | Unclear risk |
| *Reporting bias* | | |
| **Selective reporting** | Comment: trial registration: NCT01505595 | Low risk |
| *Other bias* | | |
| **Other sources of bias** | Comment: No sample size calculation. Small size. | Unclear risk |

Article: Johannsen (1994)

| **Domain** | **Support for judgment** | **Review authors’ judgement** |
| --- | --- | --- |
| *Selection bias* | | |
| **Random sequence generation** | Quote: “All patients included were randomised by minimisation, and stratified for sex, age < 40 years >, +/-preoperative hospitalisation and +/- postoperative complications, into two training models.” | Low risk |
| **Allocation concealment** | Comment: insufficient information | Unclear risk |
| *Performance bias* | | |
| **Blinding of participants and personnel** | Comment: Unable to blind participants and personnel | High risk |
| *Detection bias* | | |
| **Blinding of outcome assessment** | Comment: insufficient information | Unclear risk |
| *Attrition bias* | | |
| **Incomplete outcome data** | Quote: “Nine patients dropped out of the supervised endurance training group and 4 patients dropped out of the home training group.: Comment: High dropout rate with no intention-to-treat principle. More patients with pain dropped out of the supervised exercise group. | High risk |
| *Reporting bias* | | |
| **Selective reporting** | Comment: insufficient information | Unclear risk |
| *Other bias* | | |
| **Other sources of bias** | Comment: Sample size less than calculated required sample size due to dropouts. | Unclear risk |

Article: Ju (2012)

| **Domain** | **Support for judgment** | **Review authors’ judgement** |
| --- | --- | --- |
| *Selection bias* | | |
| **Random sequence generation** | Quote: “were randomly allocated to two group” Comment: insufficient information | Unclear risk |
| **Allocation concealment** | Comment: insufficient information | Unclear risk |
| *Performance bias* | | |
| **Blinding of participants and personnel** | Comment: Unable to blind participants and personnel | High risk |
| *Detection bias* | | |
| **Blinding of outcome assessment** | Comment: insufficient information | Unclear risk |
| *Attrition bias* | | |
| **Incomplete outcome data** | Comment: insufficient information | Unclear risk |
| *Reporting bias* | | |
| **Selective reporting** | Comment: insufficient information | Unclear risk |
| *Other bias* | | |
| **Other sources of bias** | Comment: no sample size calculation with small sample size | Unclear risk |

Article: Kemani (2024)

| **Domain** | **Support for judgment** | **Review authors’ judgement** |
| --- | --- | --- |
| *Selection bias* | | |
| **Random sequence generation** | Quote: “Allocation sequence was determined by a computerised random list with a 1:1 allocation.” | Low risk |
| **Allocation concealment** | Quotes: “An independent observer consecutively randomly assigned participants to the intervention group or to the conventional care, by drawing sealer and numbered envelopes containing the allocation condition.” | Low risk |
| *Performance bias* | | |
| **Blinding of participants and personnel** | Comment: was not possible to blind the participants or the physical therapists regarding the allocated condition. | High risk |
| *Detection bias* | | |
| **Blinding of outcome assessment** | Quote: “The independent observers involved in the assessment of the outcome measures were blinded to treatment allocation.” | Low risk |
| *Attrition bias* | | |
| **Incomplete outcome data** | Quote: “At the 12 - month follow -up, data was available for 50 participants in the intervention group and 53 in the control group. At the 24-month follow-up, data was available for 46 participants in the intervention group and 52 in the control group. Please find more detailed information on participants lost to follow-up in the Supplemental Figure.” Small number of dropouts with reasons given. | Low risk |
| *Reporting bias* | | |
| **Selective reporting** | Quote: “The study is registered with Current Controlled Trials (ISRCTN17115599) and is reported according to the Consolidated Standards of Reporting Trials (CONSORT).” | Low risk |
| *Other bias* | | |
| **Other sources of bias** | Comment: No other bias. | Low risk |

Article: Kernc (2018)

| **Domain** | **Support for judgment** | **Review authors’ judgement** |
| --- | --- | --- |
| *Selection bias* | | |
| **Random sequence generation** | Quote: “The method of concealed random allocation without blocking was used to form groups.” | Low risk |
| **Allocation concealment** | Comment: insufficient information | Unclear risk |
| *Performance bias* | | |
| **Blinding of participants and personnel** | Comment: Unable to blind participants and personnel | High risk |
| *Detection bias* | | |
| **Blinding of outcome assessment** | Comment: insufficient information | Unclear risk |
| *Attrition bias* | | |
| **Incomplete outcome data** | Quote: “Five subjects were lost during the training period and an additional seven at latest follow-up.”  Comment: no reasons for dropouts, no intention-to-treat analysis. | High risk |
| *Reporting bias* | | |
| **Selective reporting** | Quote: “The study is registered at the US National Institutes of Health (ClinicalTrials.gov) NCT03349580. The date of registration: November 21, 2017 - Retrospectively registered.” | Unclear risk |
| *Other bias* | | |
| **Other sources of bias** | Quote: “For the training period, the recommended power was achieved but not for the latest follow-up.” | Unclear risk |

Article: Kjellby-wendt (2002)

| **Domain** | **Support for judgment** | **Review authors’ judgement** |
| --- | --- | --- |
| *Selection bias* | | |
| **Random sequence generation** | Quote: “They were allocated according a table of random numbers.” | Low risk |
| **Allocation concealment** | Comment: insufficient information | Unclear risk |
| *Performance bias* | | |
| **Blinding of participants and personnel** | Comment: Unable to blind participants and personnel | High risk |
| *Detection bias* | | |
| **Blinding of outcome assessment** | Comment: insufficient information, outcome was subjective questionnaire. Participants were not blinded. | High risk |
| *Attrition bias* | | |
| **Incomplete outcome data** | Quote: “Forty-nine patients (82%) answered a questionnaire 5–7 years postoperatively. | Low risk |
| *Reporting bias* | | |
| **Selective reporting** | Comment: insufficient information | Unclear risk |
| *Other bias* | | |
| **Other sources of bias** | Comment: no sample size calculation, small sample size. | Unclear risk |

Article: Kulig (2009)

| **Domain** | **Support for judgment** | **Review authors’ judgement** |
| --- | --- | --- |
| *Selection bias* | | |
| **Random sequence generation** | Quote: “The participants were randomly allocated using blocked randomization to 1 of 2 groups” | Low risk |
| **Allocation concealment** | Comment: insufficient information | Unclear risk |
| *Performance bias* | | |
| **Blinding of participants and personnel** | Comment: Unable to blind participants and personnel | High risk |
| *Detection bias* | | |
| **Blinding of outcome assessment** | Quote: “All outcome measurements were obtained by evaluators who were blinded to the participants’ group allocation.” | Low risk |
| *Attrition bias* | | |
| **Incomplete outcome data** | Quote: “Due to a high and disproportionate dropout rate for the follow-up assessments, the analyses were conducted on preintervention and postintervention data only. Consequently, the common method of imputation of missing data became unsuitable, so analyses instead were performed only on those participants (n=77) with follow-up (ie, evaluable) data.” | High risk |
| *Reporting bias* | | |
| **Selective reporting** | Comment: The study protocol is available and all pre-specified outcomes have been reported on. | Low risk |
| *Other bias* | | |
| **Other sources of bias** | Comment: Lack of adherence to group assignment. (Third group of “usual physical therapy group” was created) Only N=14 completed education only group. Sample size much less than sample size calculation. | High risk |

Article: Lindbäck (2018)

| **Domain** | **Support for judgment** | **Review authors’ judgement** |
| --- | --- | --- |
| *Selection bias* | | |
| **Random sequence generation** | Quote: “Block randomization was used.” | Low risk |
| **Allocation concealment** | Quote: “For each randomization block, sealed opaque envelopes were prepared with a 1:1 ratio of allocation to the two groups.” | Low risk |
| *Performance bias* | | |
| **Blinding of participants and personnel** | Quote: “…whereas patients and the treating physiotherapist were not.” Comment: unable to blind. | High risk |
| *Detection bias* | | |
| **Blinding of outcome assessment** | Quote: “The physiotherapists performing the physical examination were blinded to the randomization,” | Low risk |
| *Attrition bias* | | |
| **Incomplete outcome data** | Comment: missing data have been imputed using appropriate methods. | Low risk |
| *Reporting bias* | | |
| **Selective reporting** | Quote: “The trial protocol has been  published on ClinicalTrials.gov (NCT02454400).” Comment: Study protocol is also available | Low risk |
| *Other bias* | | |
| **Other sources of bias** | Comment: The study appears to be free from other sources of bias. | Low risk |

Article: Lotzke (2019)

| **Domain** | **Support for judgment** | **Review authors’ judgement** |
| --- | --- | --- |
| *Selection bias* | | |
| **Random sequence generation** | Quote: “The allocation sequence was determined by a computerized random list with a 1:1 allocation” | Low risk |
| **Allocation concealment** | Quote: “numbered sealed envelopes containing allocation sheets of information wrapped in colored paper.” | Low risk |
| *Performance bias* | | |
| **Blinding of participants and personnel** | Quote: “Neither the participants nor the physiotherapists could be blinded” | High risk |
| *Detection bias* | | |
| **Blinding of outcome assessment** | Quote: “the independent observers responsible for the outcome measures were blinded to treatment allocation.” | Low risk |
| *Attrition bias* | | |
| **Incomplete outcome data** | Quote: “All participants were included in the ITT analysis.”  Comment: low attrition rate | Low risk |
| *Reporting bias* | | |
| **Selective reporting** | Comment: The study protocol is available and all pre-specified outcomes have been reported on. | Low risk |
| *Other bias* | | |
| **Other sources of bias** | Comment: The study appears to be free from other sources of bias. | Low risk |

Article: Lu (2025)

| **Domain** | **Support for judgment** | **Review authors’ judgement** |
| --- | --- | --- |
| *Selection bias* | | |
| **Random sequence generation** | Quote 1: “The random assignments were determined using random numbers generated by SPSS computer software”  Quote 2: “Participants were randomly assigned to either the FT group or the RH group using envelopes with generated random numbers” | Low risk |
| **Allocation concealment** | Quote: “Random allocation was achieved through sequentially numbered, sealed, and opaque envelopes” | Low risk |
| *Performance bias* | | |
| **Blinding of participants and personnel** | Quote: “The statistician and all personnel involved in the result assessment were blinded throughout the experiment. Due to the characteristics of the intervention, it was impossible to conceal the participants' assigned groups from them” | High risk |
| *Detection bias* | | |
| **Blinding of outcome assessment** | Quote: “The statistician and all personnel involved in the result assessment were blinded throughout the experiment” | Low risk |
| *Attrition bias* | | |
| **Incomplete outcome data** | Quote: “The intent-to-treat (ITT) analysis population, which included all participants who were randomized, served as the primary population for the efficacy analysis.”  Comment: Low risk of bias of attrition bias is only for walking distance outcome. | Low risk |
| *Reporting bias* | | |
| **Selective reporting** | Comment: The study protocol is available, but not all the outcomes are reported in the published version. | High risk |
| *Other bias* | | |
| **Other sources of bias** | Comment: The calculated minimum sample size required was 58 cases, accounting for an anticipated 20% dropout rate. The study randomized 52 participants, already below the necessary sample size, and experienced a much higher than anticipated dropout rate. Registration information shows that 44 per group (N=88) would be recruited. | High risk |

Article: Marchand (2021)

| **Domain** | **Support for judgment** | **Review authors’ judgement** |
| --- | --- | --- |
| *Selection bias* | | |
| **Random sequence generation** | Quote: “Randomisation and minimization were performed after the baseline assessment using a computer random number generator, prepared by a research assistant not involved in the  study process.” | Low risk |
| **Allocation concealment** | Quote: “Allocation concealment was ensured using sequentially numbered, opaque and sealed envelopes. The envelopes were opened in front of the participants by the main investigator after enrollment.” | Low risk |
| *Performance bias* | | |
| **Blinding of participants and personnel** | Quote: “Participants were not blinded to intervention allocation.” | High risk |
| *Detection bias* | | |
| **Blinding of outcome assessment** | Quote: “The principal investigator was not blind to participants’ group allocation while conducting the assessments.” | High risk |
| *Attrition bias* | | |
| **Incomplete outcome data** | Quote: “Analyses of primary and secondary outcomes were conducted according to the intention-to-treat principle with participants analyzed according to randomly assigned treatment group irrespective of compliance.” | Low risk |
| *Reporting bias* | | |
| **Selective reporting** | Comment: In the trial registry, only disability and pain were listed as outcomes. However, additional outcomes (trunk endurance, trunk flexion, lower extremity endurance, and lower extremity strength) were reported in the publication but were not preregistered. | High risk |
| *Other bias* | | |
| **Other sources of bias** | Comment: Trial powered at n=36, but less than that recruited. Unlikely to be important source of bias. | Low risk |

Article: Master (2024)

| **Domain** | **Support for judgment** | **Review authors’ judgement** |
| --- | --- | --- |
| *Selection bias* | | |
| **Random sequence generation** | Quote: “Randomization occurred electronically by research personnel 2 weeks after surgery during an in-person postoperative clinic visit. Patients were frequency-matched in a 1:1 ratio in blocks of assignments stratified by type of surgery (ie, fusion or no fusion). Block sizes of 2 and 4 were determined randomly with the patient as the unit of randomization.” | Low risk |
| **Allocation concealment** | Quote: “Randomization occurred electronically by research personnel 2 weeks after surgery during an in-person postoperative clinic visit. Patients were frequency-matched in a 1:1 ratio in blocks of assignments stratified by type of surgery (ie, fusion or no fusion). Block sizes of 2 and 4 were determined randomly with the patient as the unit of randomization.” | Low risk |
| *Performance bias* | | |
| **Blinding of participants and personnel** | Comment: Due to the nature of the intervention, researchers and patients could not be blinded. | High risk |
| *Detection bias* | | |
| **Blinding of outcome assessment** | Quote: “Outcome assessors who collected physical activity data were blinded to group assignment.” | Low risk |
| *Attrition bias* | | |
| **Incomplete outcome data** | Comment: Low level of dropouts, with reasons given. Figure 1. | Low risk |
| *Reporting bias* | | |
| **Selective reporting** | Quote: “This was a single-center feasibility randomized controlled trial (NCT04591249).” | Low risk |
| *Other bias* | | |
| **Other sources of bias** | Quote: “The funders played no role in the design, conduct, or reporting of this study.” | Low risk |

Article: Nie (2025)

| **Domain** | **Support for judgment** | **Review authors’ judgement** |
| --- | --- | --- |
| *Selection bias* | | |
| **Random sequence generation** | Quote: “According to the RANDBETWEEN (1,2) functions of Excel, the patients included in this study were prospectively assigned to conventional (singular number) or sequential exercise (even number) groups, which was performed by the third author who was able to see the whole sequence but did not participate in any of the other procedures conducted in this study.” | Low risk |
| **Allocation concealment** | Comment: No information about allocation concealment | Unclear risk |
| *Performance bias* | | |
| **Blinding of participants and personnel** | Comment: Due to the nature of the intervention, patients could not be blinded | High risk |
| *Detection bias* | | |
| **Blinding of outcome assessment** | Quote: “Both the QST and pressure biofeedback detection were performed by the same clinician (the second author), who was blinded to the group to which the patients were randomized, and another clinician (the first author), who was also blinded to the group allocation performed the imaging measurements and assisted the patients in completing the pain-related questionnaire” | Low risk |
| *Attrition bias* | | |
| **Incomplete outcome data** | Quote: “Lumbar magnetic resonance imaging (MRI) was performed for all patients before operation, and 75.4%  (298/395) of the patients further underwent lumbar MRI  3 months after operation”  Comment: no intention-to-treat analysis | High risk |
| *Reporting bias* | | |
| **Selective reporting** | Comment: Not all outcomes included with registry information even though it was retrospectively registered. QST outcome selectively applied to some participants. | High risk |
| *Other bias* | | |
| **Other sources of bias** | Comment: No a priori sample size calculation was reported. Although the total sample size (n=390) appears reasonably large, the absence of a sample size justification prevents ruling out risk of bias related to insufficient power | Unclear risk |

Article: Nielsen (2010)

| **Domain** | **Support for judgment** | **Review authors’ judgement** |
| --- | --- | --- |
| *Selection bias* | | |
| **Random sequence generation** | Quote: “The allocation was based on computer randomization in blocks of 10 patients, to either integrated programme or standard programme.” | Low risk |
| **Allocation concealment** | Quote: “Information on intervention or routine procedure was enclosed in sealed opaque envelopes with consecutive numbers.” | Low risk |
| *Performance bias* | | |
| **Blinding of participants and personnel** | Quote: “it would be impossible to blind patients.” | High risk |
| *Detection bias* | | |
| **Blinding of outcome assessment** | Quote: “The physiotherapist and a nurse especially trained in pain score, intervention and follow-up performed all assessments. They were not member of the clinical staff and did not participate in the daily patient care. They were not blinded, however, because we could not guarantee that the patients would not disclose their allocation group” | High risk |
| *Attrition bias* | | |
| **Incomplete outcome data** | Comment: no patient lost to follow up that received intervention. | Low risk |
| *Reporting bias* | | |
| **Selective reporting** | Quote: “the study has been registered in the international protocol registration system www.ClinicalTrails.gov, ID NCT 00459966.” | Low risk |
| *Other bias* | | |
| **Other sources of bias** | Comment: The study appears to be free from other sources of bias. | Low risk |

Article: Oestergaard (2013)

| **Domain** | **Support for judgment** | **Review authors’ judgement** |
| --- | --- | --- |
| *Selection bias* | | |
| **Random sequence generation** | Quote: “The patients were block-randomized at the spine centre from which they were allocated.” | Low risk |
| **Allocation concealment** | Quote: “They were randomly assigned to the 6w-group or the 12w-group by use of sealed envelopes.” | Low risk |
| *Performance bias* | | |
| **Blinding of participants and personnel** | Quote: “The conditions of the study allowed no blinding of the therapists, surgeons, or the patients.” | High risk |
| *Detection bias* | | |
| **Blinding of outcome assessment** | Quote: “The conditions of the study allowed no blinding of the therapists, surgeons, or the patients.” | High risk |
| *Attrition bias* | | |
| **Incomplete outcome data** | Comment: Reasons for dropout or missing data described and similar in both groups. | Low risk |
| *Reporting bias* | | |
| **Selective reporting** | Comment: insufficient information | Unclear risk |
| *Other bias* | | |
| **Other sources of bias** | Comment: The study appears to be free from other sources of bias. | Low risk |

Article: Sobanski (2025)

| **Domain** | **Support for judgment** | **Review authors’ judgement** |
| --- | --- | --- |
| *Selection bias* | | |
| **Random sequence generation** | Quote: “Randomisation was performed using the software MATLAB (MathWorks, Inc. 2018, Massachusetts) with RARtool interface” | Low risk |
| **Allocation concealment** | Quote: “The person responsible for randomization also managed the list of patients divided into groups and informed the physiotherapist in charge of training which patients were assigned to the experimental group  and which to the control group.”  Comment: The same individual generated the random sequence, managed the allocation list, and informed the physiotherapist of group assignments. No description of a secure concealment mechanism (e.g., sealed opaque envelopes, central allocation) was provided, raising the possibility that group assignments could have been foreseen or influenced. | Unclear risk |
| *Performance bias* | | |
| **Blinding of participants and personnel** | Comment: Due to the nature of the intervention, patients could not be blinded | High risk |
| *Detection bias* | | |
| **Blinding of outcome assessment** | Quote: “Initial and final assessments  were conducted by a physiotherapist who was blinded to the subjects’ group assignments and had no involvement in their training” | Low risk |
| *Attrition bias* | | |
| **Incomplete outcome data** | Comment: All the participants in the initial analysis completed the study | Low risk |
| *Reporting bias* | | |
| **Selective reporting** | Comment: No registration or protocol available | Unclear risk |
| *Other bias* | | |
| **Other sources of bias** | Comment: The study appears to be free from other sources of bias | Low risk |

Article: Son (2025)

| **Domain** | **Support for judgment** | **Review authors’ judgement** |
| --- | --- | --- |
| *Selection bias* | | |
| **Random sequence generation** | Quote: “Patients were randomly assigned to either the exercise group or control group”  Comment: insufficient information about the randomization process | Unclear risk |
| **Allocation concealment** | Comment: Insufficient information about the allocation concealment process | Unclear risk |
| *Performance bias* | | |
| **Blinding of participants and personnel** | Quote: “Due to the nature of the face-to-face exercise program, blinding of participants and investigators was not feasible” | High risk |
| *Detection bias* | | |
| **Blinding of outcome assessment** | Quote: “Due to the nature of the face-to-face exercise program, blinding of participants and investigators was not feasible” | High risk |
| *Attrition bias* | | |
| **Incomplete outcome data** | Quote: “Of the 40 participants initially registered, 37 were included in the final cohort”  Comment: low dropout with reasons | Low risk |
| *Reporting bias* | | |
| **Selective reporting** | Comment: SF-36 registered not reported. Conflicting declarations of primary/secondary outcomes between registry and report. | High risk |
| *Other bias* | | |
| **Other sources of bias** | Comment: The study appears to be free from other sources of bias | Low risk |

Article: Takenaka (2025)

| **Domain** | **Support for judgment** | **Review authors’ judgement** |
| --- | --- | --- |
| *Selection bias* | | |
| **Random sequence generation** | Quote: “A total of 32 patients (mean age: 69.3 y, comprising 17 women) were randomly assigned to either the intervention group (n =15) or the control group (n =17) using a computer-generated random number table” | Low risk |
| **Allocation concealment** | Comment: Insufficient information about the procedure | Unclear risk |
| *Performance bias* | | |
| **Blinding of participants and personnel** | Comment: Due to the nature of the intervention, patients could not be blinded | High risk |
| *Detection bias* | | |
| **Blinding of outcome assessment** | Quote: “The surgeon (M.K.), postoperative rehabilitation personnel, and postoperative evaluators were blinded to group assignments” | Low risk |
| *Attrition bias* | | |
| **Incomplete outcome data** | Comment: important number of drop-outs in the prehabilitation group. | High risk |
| *Reporting bias* | | |
| **Selective reporting** | Comment: Insufficient information | Unclear risk |
| *Other bias* | | |
| **Other sources of bias** | Comment: The study appears to be free from other sources of bias | Low risk |

Article: Tegner (2024)

| **Domain** | **Support for judgment** | **Review authors’ judgement** |
| --- | --- | --- |
| *Selection bias* | | |
| **Random sequence generation** | Quote: “computer-generated randomization list based on permuted random blocks of variable size was generated before enrollment of participants.” | Low risk |
| **Allocation concealment** | Quote: “Individual allocations were held in sealed opaque envelopes until the intervention-physiotherapists had delivered the envelope to the included participants.” | Low risk |
| *Performance bias* | | |
| **Blinding of participants and personnel** | Quote: “Neither the participants nor the intervention-physiotherapists were blinded to allocation sequence after allocation” | High risk |
| *Detection bias* | | |
| **Blinding of outcome assessment** | Quote: “Outcome assessors were 3 physiotherapists not otherwise involved in the study and were blinded to participants’ randomization and the content of GAPE.” | Low risk |
| *Attrition bias* | | |
| **Incomplete outcome data** | Quote: “The primary analysis was performed using the intention-to-treat (ITT) population, defined as all randomized participants with com- plete baseline data on the primary outcome, named the modified ITT (mITT) population. Outcomes were analyzed as change from baseline using repeated measures mixed linear models, including participants as random effects, with fixed effect factors for group and week (including all timepoints to respect the ITT principle) and the corresponding interaction, while adjusting for baseline values and the strat- ification factors. Missing data were handled implicitly in the ITT analysis by the mixed linear models under the missing at random assumption.” | Low risk |
| *Reporting bias* | | |
| **Selective reporting** | Comment: “The protocol was registered a priori at www.clinicaltrials.gov (NCT04103970) and submitted to the Health Research Ethics Committee of the Capital Region of Denmark (H-19024440).” | Low risk |
| *Other bias* | | |
| **Other sources of bias** | Comment: No conflict of interest statement. | Unclear risk |

Article: Yílmaz (2003)

| **Domain** | **Support for judgment** | **Review authors’ judgement** |
| --- | --- | --- |
| *Selection bias* | | |
| **Random sequence generation** | Quote: “Patients were divided randomly into 3 treatment groups.” Comment: insufficient information | Unclear risk |
| **Allocation concealment** | Comment: insufficient information | Unclear risk |
| *Performance bias* | | |
| **Blinding of participants and personnel** | Comment: Unable to blind participants and personnel | High risk |
| *Detection bias* | | |
| **Blinding of outcome assessment** | Comment: insufficient information | Unclear risk |
| *Attrition bias* | | |
| **Incomplete outcome data** | Comment: insufficient information | Unclear risk |
| *Reporting bias* | | |
| **Selective reporting** | Comment: insufficient information | Unclear risk |
| *Other bias* | | |
| **Other sources of bias** | Comment: no sample size calculation, small sample sizes. | Unclear risk |
