# Supplementary material for: Effects of rehabilitation and behavior change interventions on physical capacity and physical activity behavior following lumbar surgery for degenerative disease: A systematic review and meta-analysis
Source: PLoS One. 2026 Apr 20;21(4):e0347420. doi: 10.1371/journal.pone.0347420 (PMC13094952; doi:10.1371/journal.pone.0347420)
Supplement: S5 File — (DOCX) [file pone.0347420.s007.docx]

**S5 File. GRADE TABLES**

**Effects of rehabilitation and behavior change interventions on physical capacity and physical activity behavior following lumbar surgery for degenerative disease: a systematic review and meta-analysis.**

**1. Question:** Exercise compared to Minimal/Usual care for trunk extension endurance – Immediate (Figure 4)

| **Certainty assessment** | | | | | | | **№ of patients** | | **Effect** | | **Certainty** | **Importance** |
| --- | --- | --- | --- | --- | --- | --- | --- | --- | --- | --- | --- | --- |
| **№ of studies** | **Study design** | **Risk of bias** | **Inconsistency** | **Indirectness** | **Imprecision** | **Other considerations** | **Exercise** | **Minimal/Usual care** | **Relative (95% CI)** | **Absolute (95% CI)** |  |  |
| **Trunk extension endurance - Immediate** | | | | | | | | | | | | |
| 4 | randomised trials | serious^a^ | not serious | not serious | Serious^b^ | none | 168 | 90 | - | SMD **1.54 SD higher** (0.93 higher to 2.16 higher) | ⨁⨁◯◯ Low^a,b^ |  |

**CI:** confidence interval; **SMD:** standardised mean difference

#### Explanations

a. > 50% of sample from studies not rated as low risk of bias

b. Sample size < 400

**2. Question:** Supervised exercise compared to Self-directed exercise for trunk extension endurance – Immediate (Figure 5)

| **Certainty assessment** | | | | | | | **№ of patients** | | **Effect** | | **Certainty** | **Importance** |
| --- | --- | --- | --- | --- | --- | --- | --- | --- | --- | --- | --- | --- |
| **№ of studies** | **Study design** | **Risk of bias** | **Inconsistency** | **Indirectness** | **Imprecision** | **Other considerations** | **Supervised exercise** | **Self-directed exercise** | **Relative (95% CI)** | **Absolute (95% CI)** |  |  |
| **Trunk extension endurance - Immediate** | | | | | | | | | | | | |
| 2 | randomised trials | serious^a^ | not serious | not serious | serious^b^ | none | 34 | 34 | - | SMD **1.28 SD higher** (0.75 higher to 1.81 higher) | ⨁⨁◯◯ Low^a,b^ |  |

**CI:** confidence interval; **SMD:** standardised mean difference

#### Explanations

a. > 50% of sample from studies not rated as low risk of bias

b. Sample size < 400

**3. Question:** Psychologically informed rehabilitation compared to Minimal/Usual care for physical activity – Immediate and Intermediate (Figure 6)

| **Certainty assessment** | | | | | | | **№ of patients** | | **Effect** | | **Certainty** | **Importance** |
| --- | --- | --- | --- | --- | --- | --- | --- | --- | --- | --- | --- | --- |
| **№ of studies** | **Study design** | **Risk of bias** | **Inconsistency** | **Indirectness** | **Imprecision** | **Other considerations** | **Directed exercise and manual therapy** | **self-directed/group exercise** | **Relative (95% CI)** | **Absolute (95% CI)** |  |  |
| **Physical activity – Immediate** | | | | | | | | | | | | |
| 2 | randomised trials | serious^a^ | not serious | not serious | serious^b^ | none | 76 | 77 | - | SMD **0.17 SD higher** (0.14 lower to 0.49 higher) | ⨁⨁◯◯ Low^a,b^ |  |
| **Physical activity – Intermediate** | | | | | | | | | | | | |
| 3 | randomised trials | serious^a^ | not serious | not serious | serious^b^ | none | 136 | 136 | - | SMD **0.26 SD higher** (0.02 higher to 0.49 higher) | ⨁⨁◯◯ Low^a,b^ |  |

**CI:** confidence interval; **SMD:** standardised mean difference

#### Explanations

a. > 50% of sample from studies not rated as low risk of bias

b. Sample size < 400

**4. Question:** Physical activity advice compared to Minimal/Usual care for physical activity – Intermediate (Figure 7)

| **Certainty assessment** | | | | | | | **№ of patients** | | **Effect** | | **Certainty** | **Importance** |
| --- | --- | --- | --- | --- | --- | --- | --- | --- | --- | --- | --- | --- |
| **№ of studies** | **Study design** | **Risk of bias** | **Inconsistency** | **Indirectness** | **Imprecision** | **Other considerations** | **Physical activity advice** | **Minimal/Usual care** | **Relative (95% CI)** | **Absolute (95% CI)** |  |  |
| **Physical activity - Intermediate** | | | | | | | | | | | | |
| 2 | randomised trials | not serious | not serious | not serious | serious^a^ | none | 66 | 67 | - | SMD **0.21 SD higher** (0.13 lower to 0.55 higher) | ⨁⨁⨁◯ Moderate^a^ |  |

**CI:** confidence interval; **SMD:** standardised mean difference

#### Explanations

a. Sample size < 400

**5. Question:** Prehabilitation compared to Minimal/Usual care for physical activity – Intermediate (Figure 8)

| **Certainty assessment** | | | | | | | **№ of patients** | | **Effect** | | **Certainty** | **Importance** |
| --- | --- | --- | --- | --- | --- | --- | --- | --- | --- | --- | --- | --- |
| **№ of studies** | **Study design** | **Risk of bias** | **Inconsistency** | **Indirectness** | **Imprecision** | **Other considerations** | **Prehabilitation** | **Minimal/Usual care** | **Relative (95% CI)** | **Absolute (95% CI)** |  |  |
| **Physical activity - Intermediate** | | | | | | | | | | | | |
| 2 | randomised trials | serious^a^ | not serious | not serious | serious^b^ | none | 129 | 128 | - | SMD **0.28 SD higher** (0.03 higher to 0.53 higher) | ⨁⨁◯◯ Low^a,b^ |  |

**CI:** confidence interval; **SMD:** standardised mean difference

#### Explanations

a. > 50% of sample from studies not rated as low risk of bias

b. Sample size < 400
